# Apelin analog treatment reverses severe pulmonary arterial hypertension and right ventricular heart failure

**DOI:** 10.1172/jci.insight.200684

**Published:** 2026-06-18

**Authors:** Jennie Vu, Pavel Zhabyeyev, Kemar J. Brown, Joshua Gorham, Daniel M. DeLaughter, Huachen Chen, Thilina U. Jayawardena, Ander Vergara, Maria Alexiou, Anjalee Wijewardane, Conrad Fischer, Charlotte Avet, Abby Ewasiuk, Faqi Wang, Mark C. Chappell, Yuri Kim, Michel Bouvier, John C. Vederas, Christine E. Seidman, Jonathan G. Seidman, Gavin Y. Oudit

**Affiliations:** 1 Department of Physiology and; 2 Department of Medicine, University of Alberta, Edmonton, Alberta, Canada.; 3 Department of Genetics, Harvard Medical School, Boston, Massachusetts, USA.; 4 Division of Cardiovascular Medicine, Massachusetts General Hospital, Boston, Massachusetts, USA.; 5 Department of Dentistry & Dental Hygiene, University of Alberta, Edmonton, Alberta, Canada.; 6 Department of Chemistry, University of Alberta, Edmonton, Alberta, Canada.; 7 Institute for Research in Immunology and Cancer, Université de Montréal, Montréal, Quebec, Canada.; 8 Department of Surgery/Hypertension and Vascular Research, Wake Forest School of Medicine, Winston-Salem, North Carolina, USA.; 9 Division of Cardiovascular Medicine, Brigham and Women’s Hospital, Boston, Massachusetts, USA.; 10 Mazankowski Alberta Heart Institute, Alberta Health Services, Edmonton, Alberta, Canada.

**Keywords:** Cardiology, Pulmonology, Vascular biology, Angiogenesis, Cardiovascular disease, Nitric oxide

## Abstract

Pulmonary arterial hypertension (PAH) is a progressive vascular syndrome characterized by aberrant signaling, severe pulmonary artery remodeling, and right ventricular (RV) failure, a major driver of morbidity and mortality. Dysregulation of the apelinergic pathway has been implicated in pulmonary vascular remodeling in PAH. Using a sugen-hypoxia rat model of PAH, we assessed the ability of a potentially novel apelin analog, resistant to native peptidase degradation, to reverse the pathological hallmarks of PAH and RV dysfunction. Apelin analog therapy corrected the vascular lesions in the lungs and nearly normalized pulmonary arterial pressures. Early cardiorenal syndrome, RV dilation, and dysfunction, as well as RV cardiomyocyte and fibroblast activation induced by pressure overload, were also reversed by apelin analog treatment. Single-nucleus RNA-seq of the lungs and RV revealed apelin-analog treatment activated several protective pathways, including rebalancing protective bone morphogenetic protein receptor type 2 (BMPR2) signaling to counteract excessive pathogenic TGF-β receptor 2 (TGFBR2) activity in PAH. These findings highlight the therapeutic potential of exogenous apelin in reversing pulmonary vascular and cardiac pathologies in PAH and support further investigation to evaluate the clinical benefits of apelin analog treatment in patients with PAH and RV failure.

## Abstract 2

Pulmonary arterial hypertension and right ventricular failure were successfully treated with a novel apelin analog with an extended half life.

## Graphical abstract

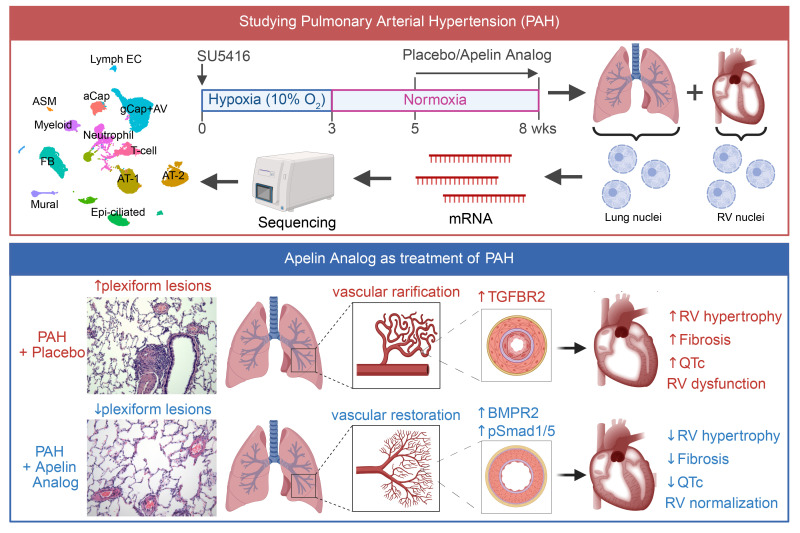

## Introduction

Pulmonary arterial hypertension (PAH) is a debilitating disease characterized by obliterative vascular remodeling of precapillary pulmonary vasculature concomitant with endothelial dysfunction and lumen-narrowing neointimal accumulations leading to high pulmonary arterial pressures (1, 2). The right ventricle (RV), burdened with chronic pressure overload, adversely remodels leading to pathological hypertrophy, chamber dilation, and progression to right-sided heart failure. RV failure is pathognomonic of late-stage PAH and is a major determinant of mortality (3, 4). Existing PAH therapies target the endothelin, prostacyclin, and guanylate cyclase/nitric oxide pathways (3). However, despite the temporary alleviation of high pulmonary pressures by these therapies, PAH remains a progressive disease with limited therapeutic options and high mortality. Recently, an important connection between disrupted apelin pathway signaling and the development of PAH was drawn by several groups. Apelin levels are reduced in the plasma of patients with PAH and are altered in the RV and pulmonary vasculature in both patients and animal models (5–7). Apelin transcripts in the RV are downregulated specifically in rats with decompensated RV failure, while they are preserved in well-adapted RV in animal models of PAH (6, 8).

Apelin is an endogenous peptide family with apelin-13 and apelin-17 being the dominant isoforms (9–11). We designed and synthesized a stable analog of apelin-17, designated CbzPEG6-NMeLeu-A2-17, that evades enzymatic breakdown by incorporating N-methyl leucine and modifying the carboxyl and amino termini of the peptide (12–14). Using the sugen-hypoxia (SU/Hx) experimental model (15, 16), which closely simulates the pathophysiological and clinical manifestations of human PAH, our study investigates the efficacy of a metabolically stable apelin-17 peptide analog in reversing the progression of PAH and alleviating disease burden. By leveraging single-nucleus RNA-seq (snRNA-seq), we illuminated cell type compositions, transcriptional cell states, and molecular pathways altered in lungs and RVs from untreated and apelin-treated rats with PAH. snRNA-seq allowed us to glean seminal, unbiased insights into mechanisms of apelinergic rescue of myocardial remodeling, electrical remodeling, metabolic perturbations, inflammation, and disrupted vascular quiescence. We identified CbzPEG6-NMeLeu-A2-17 as a promising, potentially novel therapeutic approach to reverse the fundamental pathophysiological basis of PAH.

## Results

### Apelin analog reverses vascular injury and right ventricular pressure overload in PAH.

**Figure 1:**
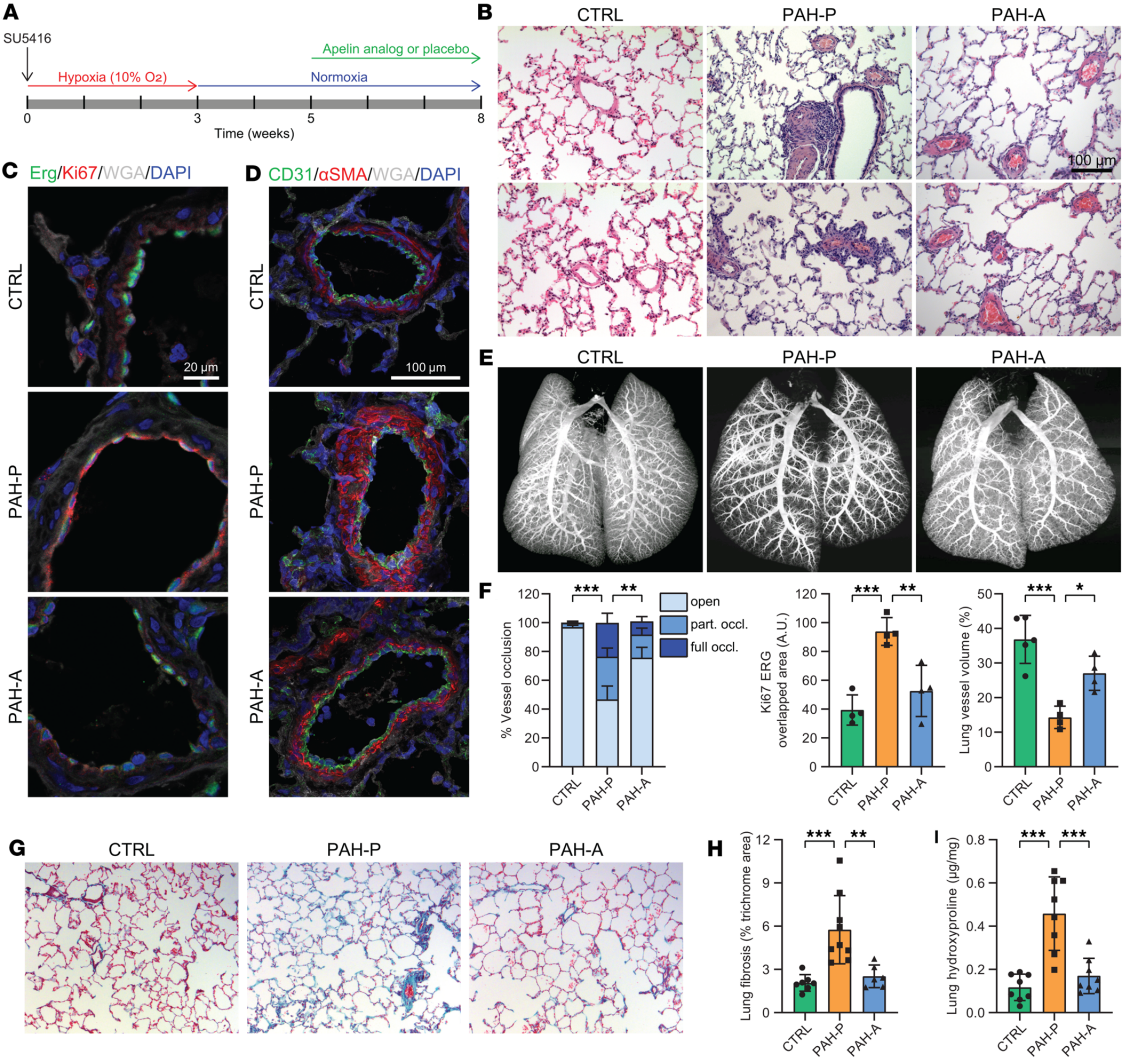
Apelin analog treatment reverses pulmonary vascular remodeling and fibrosis in pulmonary arterial hypertension. (**A**) Schematic of overall experimental design. (**B**) Representative images of H&E staining of the lung sections highlighting the vascular plexiform lesions in larger vessels (top middle panel) and smaller vessels (lower middle panel). Scale bar: 100 µm. (**C**) Representative images of immunofluorescent costaining of lung sections with Erg (green), Ki67 (red), wheat germ agglutinin (WGA; gray), and DAPI (blue). Scale bar: 20 µm. (**D**) Representative images of immunofluorescent costaining of lung sections with CD31 (green), α-smooth muscle actin (αSMA; red), WGA (gray), and DAPI (blue). Scale bar: 100 µm. (**E**) μCT of the lungs following ex vivo perfusion of barium contrast into the main pulmonary artery. (**F**) Quantification of vascular lesions (left), Ki67^+^ staining (middle), and lung vessel volume relative to the entire lung volume (right). (**G**) Representative images of trichrome staining of the lung tissue. (**H**) Quantification of pulmonary fibrosis based on trichrome staining. (**I**) Lung hydroxyproline content. CTRL, control rats; PAH-P, PAH rats treated with placebo and PAH-A, PAH rats treated with apelin analog. *n* = 6 tissue sections, *n* = 8–12 animals. Data are shown as mean ± SD. Comparisons are done with 1-way ANOVA Tukey’s post hoc analysis for multiple comparisons. *P < 0.05, **P < 0.01, ***P < 0.001.

PAH was induced by the SU/Hx protocol, and male rats with PAH were randomized to receive either a placebo (PAH-P) or the apelin analog, CbzPEG6-NMeLeu-A2-17 (PAH-A), as a therapeutic intervention to reverse PAH (Figure 1A). The model recapitulated characteristic histological features of PAH: (a) plexiform lesions defined morphologically (Figure 1B) (16, 17) with increased proliferation of endothelial cells indicated by Ki67 and Erg costaining (Figure 1C) and (b) endothelial-mesenchymal transition as suggested by expansion of the mesenchymal marker, α smooth muscle actin (αSMA) (Figure 1D). μCT of contrast perfused ex vivo lungs confirmed global loss of the perfusing pulmonary vasculature in PAH-P (Figure 1E). Quantification of these notable vascular changes showed that PAH-P animals had a higher fraction of fully and partially occluded vessels, greater levels of proliferating endothelial cells, and lower total intravascular volume relative to total lung volume (Figure 1F). We designed and synthesized a stable analog of apelin-17, CbzPEG6-NMeLeu-A2-17 (12–14), which is resistant to breakdown by kallikrein, neprilysin, and ACE2 (Supplemental Figure 1A; supplemental material available online with this article; https://doi.org/10.1172/jci.insight.200684DS1). These modifications maintained the specificity and activation profile of the peptide (Supplemental Figure 1B), while resulting in marked protection from in vitro proteolytic degradation with increased half-life in rat and human plasma (Supplemental Figure 1, C and D). We showed that, in control rats, treatment with the apelin analog for 3 weeks resulted in no metabolic alteration, changes in cardiac electrical activity, cardiac function, or pulmonary pressures (Supplemental Figure 2, A and B). Similarly, lung vessel structure and endothelial proliferation were not altered by the apelin analog treatment in control rats (Supplemental Figure 2, C and D).

**Figure 2:**
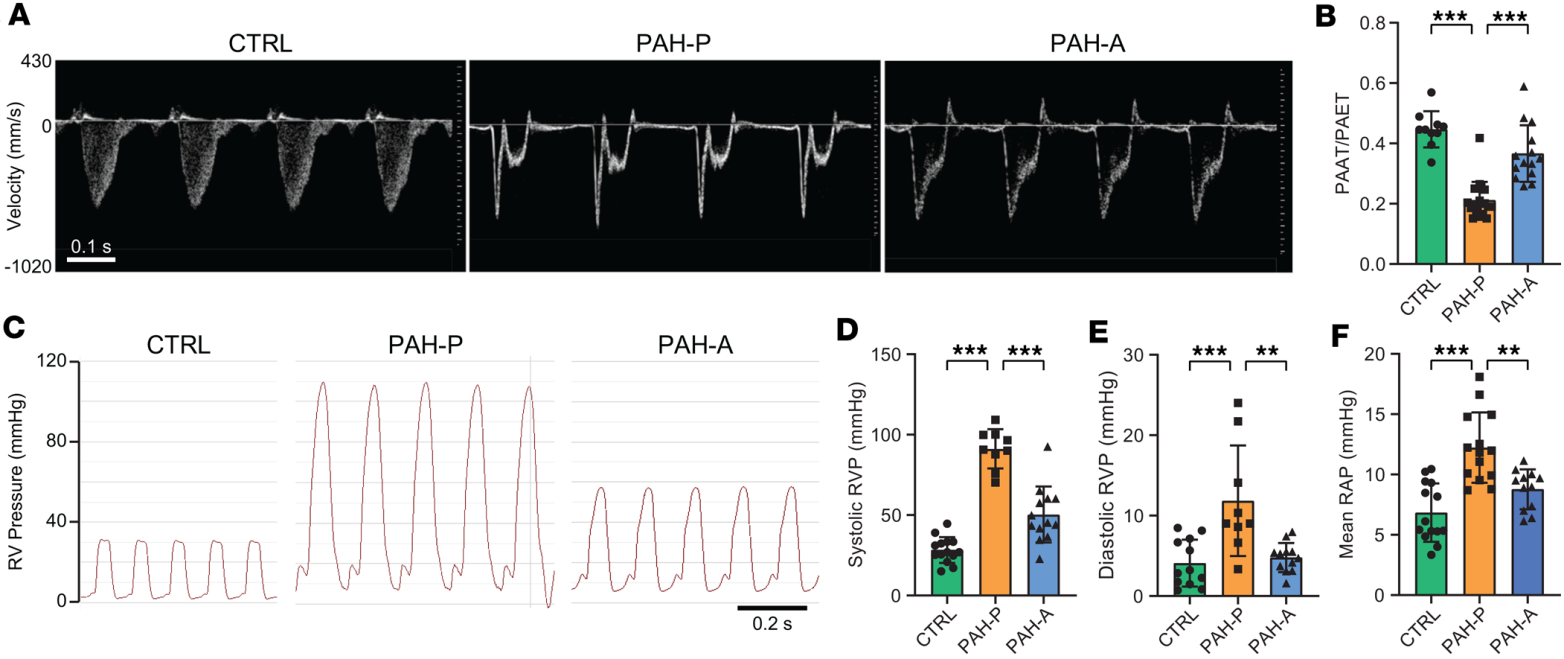
Apelin analog treatment reverses pulmonary hemodynamic abnormalities in pulmonary arterial hypertension. (**A**) Representative pulse-wave Doppler images of the pulmonary arterial flow. (**B**) Quantification of the ratio of pulmonary artery acceleration time (PAAT) to pulmonary artery ejection time (PAET). (**C**) Representative traces of invasive right ventricular (RV) pressure across 5 cardiac cycles. (**D**–**F**) Systolic RV pressures, diastolic RV pressures, and mean right atrial (RA) pressures. CTRL, control rats; PAH-P, PAH rats treated with placebo and PAH-A, PAH rats treated with apelin analog. *n* = 8–12 animals. Data are shown as mean ± SD. Comparisons are done with 1-way ANOVA Tukey’s post hoc analysis for multiple comparisons. **P < 0.01, ***P < 0.001.

In our rat model of PAH, apelin analog treatment reduced the instances of plexiform lesions (Figure 1B), decreased the fraction of occluded vessels (Figure 1, B and F), partially restored lung vessel volume (Figure 1, E and F), normalized endothelial proliferation (Figure 1, C and F), and moderated mesenchymal expansion (Figure 1D). Additionally, apelin analog treatment reduced diffuse pulmonary fibrosis seen in the untreated PAH group (Figure 1, G–I, and Supplemental Figure 3A). Vascular pathological remodeling and extracellular matrix (ECM) deposition were reversed by apelin analog treatment in the absence of systemic metabolic and body composition changes (Figure 1, C–I, and Supplemental Figure 3, B–D). The apelin analog showed no hepatotoxicity based on liver histology, morphometry, plasma bilirubin, and markers of hepatocellular injury (Supplemental Figure 4). Elevation of pulmonary and RV pressure is the central hemodynamic characteristic of PAH. Transthoracic echocardiography showed pronounced midsystolic notching in the pulse wave Doppler profile of pulmonary artery flow in the PAH-P group, indicative of downstream vascular stiffening (Figure 2A). The ratio of pulmonary artery acceleration time (PAAT) to pulmonary artery ejection time (PAET) was reduced in PAH-P and was accompanied by midsystolic notching (Figure 2B), denoting increased pulmonary vascular resistance and pressures. The PAAT/PAET ratio reached pathological levels with typical midsystolic notching by 5 weeks (Supplemental Figure 5, A and B) associated with dilated RV and reduced systolic function (Supplemental Figure 5, C–E) indicating that an established PAH phenotype of high pulmonary pressure was present when treatment with the analog started. Invasive hemodynamic measurements at week 8 showed marked elevation of the RV systolic pressure (RVSP) in the PAH-P group corroborating the findings of elevated pulmonary artery systolic pressures in those animals (Figure 2, C and D). Right ventricular end-diastolic pressure (diastolic RVP) and mean right atrial pressure (mean RAP) were also increased within PAH-P group (Figure 2, E and F). The apelin-analog treated group (PAH-A) had diminished midsystolic notching and restored PAAT/PAET ratio (Figure 2, A and B) and normalized RVPs and mean RAP (Figure 2, D–F) resembling control (CTRL) hearts. Our results demonstrate that treatment with apelin analog reversed the dominant vascular, histological, and hemodynamic changes generated in the SU/Hx model of PAH.

### Apelin analog protects RV structure, RV function, and renal function in PAH.

**Figure 3:**
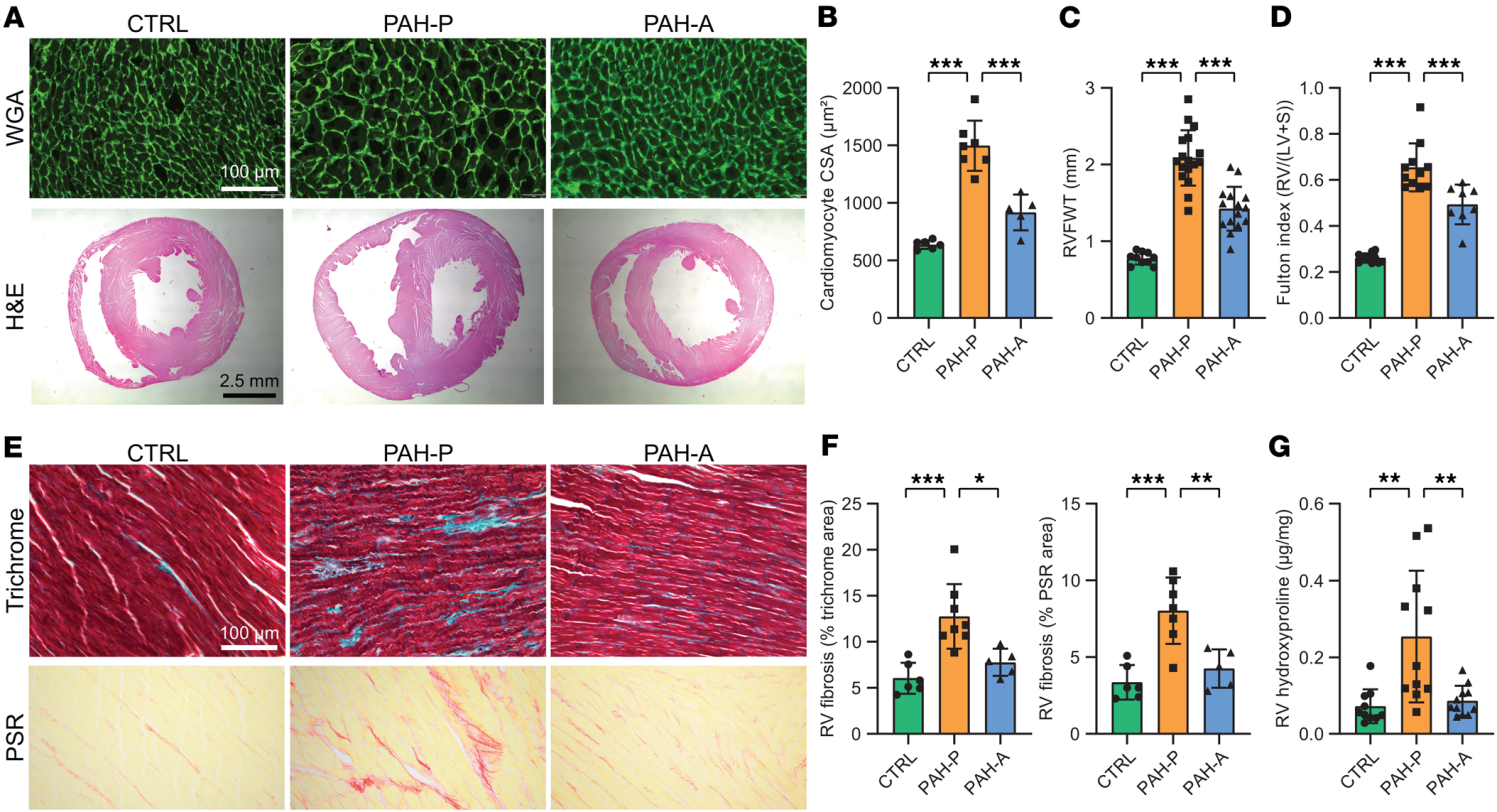
Apelin analog treatment reverses right ventricular hypertrophy and fibrosis in pulmonary arterial hypertension. (**A**) Representative images of wheat germ agglutinin (WGA) staining (top panels, scale bar: 100 µm) and H&E staining of the short-axis view of the whole heart (bottom panels, scale bar: 2.5 µm). (**B**) Cardiomyocyte cross-sectional area (CSA) from WGA staining quantification. (**C**) Right ventricular free wall thickness (RVFWT) based on echocardiographic assessment. (**D**) Fulton index (RV/[LV+S]: ratio of RV to LV weight including septum). (**E**) Representative images of trichrome (top panels) and picrosirius red (PSR) (bottom panels) staining. (**F**) Quantification of fibrosis based on Trichrome^+^ (left) and PSR^+^ area (right). (**G**) Hydroxyproline assay of RV collagen content. CTRL, control rats; PAH-P, PAH rats treated with placebo and PAH-A, PAH rats treated with apelin analog. RV, right ventricle; LV, left ventricle. *n* = 5 tissue sections, *n* = 12–18 animals. Data are shown as mean ± SD. Comparisons are done with 1-way ANOVA Tukey’s post hoc analysis for multiple comparisons. *P < 0.05, **P < 0.01, ***P < 0.001.

**Figure 4:**
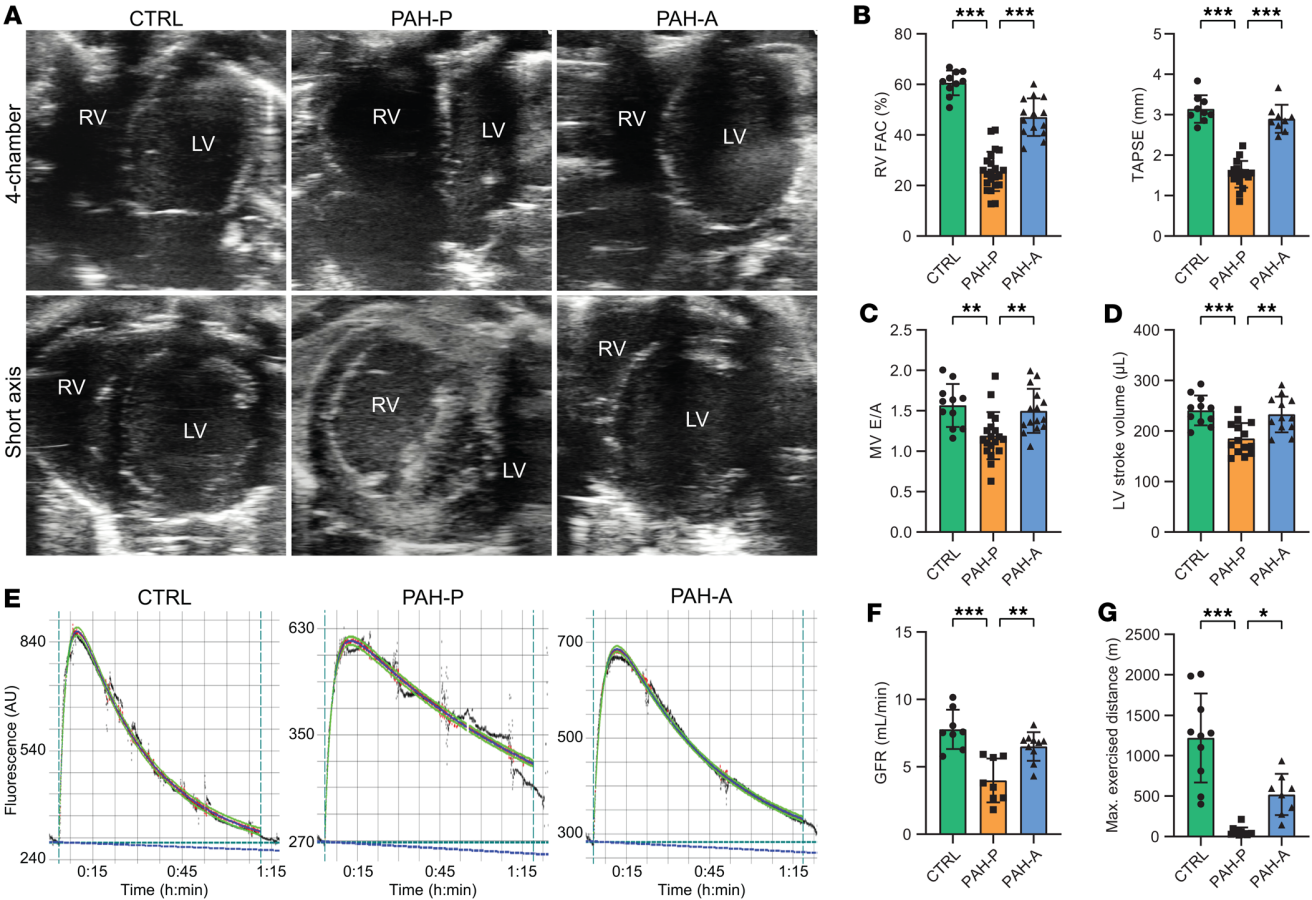
Apelin analog treatment restores right ventricular function, renal perfusion, and exercise capacity in pulmonary arterial hypertension. (**A**) Representative B-mode images of 4-chamber views (top panels) and short-axis views (bottom panels). (**B**) Right ventricle fractional area change (RVFAC) and tricuspid annular plane systolic excursion (TAPSE). (**C**) LV mitral valve early diastolic filling flow versus atrial diastolic filling flow, E/A ratio. (**D**) LV stroke volume. (**E**) Representative traces of transcutaneous fluorescence of fluorescein isothiocyanate bounded sinistrin (FITC-sinistrin) (administered intravenously). (**F**) Glomerular filtration rate (GFR) measured from transcutaneous fluorescence. (**G**) Maximal exercise capacity as maximal distance run on treadmill. CTRL, control rats; PAH-P, PAH rats treated with placebo and PAH-A, PAH rats treated with apelin analog. RV, right ventricle; LV, left ventricle. *n* = 5 tissue sections, *n* = 12–18 animals. Data are shown as mean ± SD. Comparisons are done with 1-way ANOVA Tukey’s post hoc analysis for multiple comparisons. *P < 0.05, **P < 0.01, ***P < 0.001.

The effects of apelin analog on PAH rat cardiac morphology were first assessed by histologic analyses. The PAH-P RV tissue stained with wheat germ agglutin (WGA) and H&E exhibited striking cellular hypertrophy (Figure 3, A and B), thickened RV free wall (Figure 3C), an increased Fulton index (Figure 3D), and a dilated RV chamber (Figure 3A). Masson’s trichrome and picrosirius red staining (PSR) (Figure 3, E and F) revealed diffuse RV fibrosis in the PAH-P group. Quantification of hydroxyproline content confirmed elevated collagen content in PAH-P hearts (Figure 3G). In contrast, the left ventricle (LV) was not hypertrophic and lacked myocardial fibrosis in response to PAH confirming a RV-specific pathology in the SU/Hx model (Supplemental Figure 6). Treatment with apelin analog largely reversed the PAH-associated pathological cellular and morphological changes in the RV (Figure 3A), moderately reduced hypertrophy (Figure 3, A–D), and effectively mitigated fibrosis (Figure 3, E–G). Echocardiography demonstrated pathological hallmarks of right ventricular pressure overload in PAH-P rats, including pronounced RV dilation, paradoxical septal movement, and ventricular interdependence (Figure 4A, Supplemental Videos 1 and 2, and Supplemental Table 1). RV fractional area change (RVFAC) and tricuspid annular plane systolic excursion (TAPSE), established indices of RV systolic function, were both diminished in PAH-P animals (Figure 4B). Notably, we observed reduced LV E/A ratio (Figure 4C), suggestive of impaired LV filling secondary to ventricular interdependence, concomitant with reduced LV stroke volume (Figure 4D) in PAH-P rats. The RV and LV systolic function were diminished by week 5, before exposure to placebo or apelin analog (Supplemental Figure 5). Apelin analog therapy preserved RV structure and function as evidenced by restored echocardiographic and hemodynamic parameters (Figure 4, A–D; Supplemental Videos 1 and 2; and Supplemental Table 1). RV dilation and reduced systolic function culminated in RV failure, venous congestion, and precipitation of cardiorenal syndrome. PAH-P rats exhibited compromised glomerular filtration rates (GFR), a pathognomonic feature of early cardiorenal syndrome, despite the absence of intrinsic structural glomerular damage and albuminuria (Figure 4, E and F, and Supplemental Figure 7). In response to apelin analog treatment, GFR was nearly normalized (Figure 4, E and F). The combination of PAH and RV dysfunction resulted in attenuated exercise capacity in PAH-P rats, which was partially restored with apelin analog intervention (Figure 4G). Collectively, these results demonstrate the beneficial effects of apelin analog in preserving RV structure and function with a substantive reversal of the cardiorenal syndrome and improvement in exercise capacity.

### Altered cell-based pathogenic pathways in the lungs are suppressed by apelin analog.

**Figure 5:**
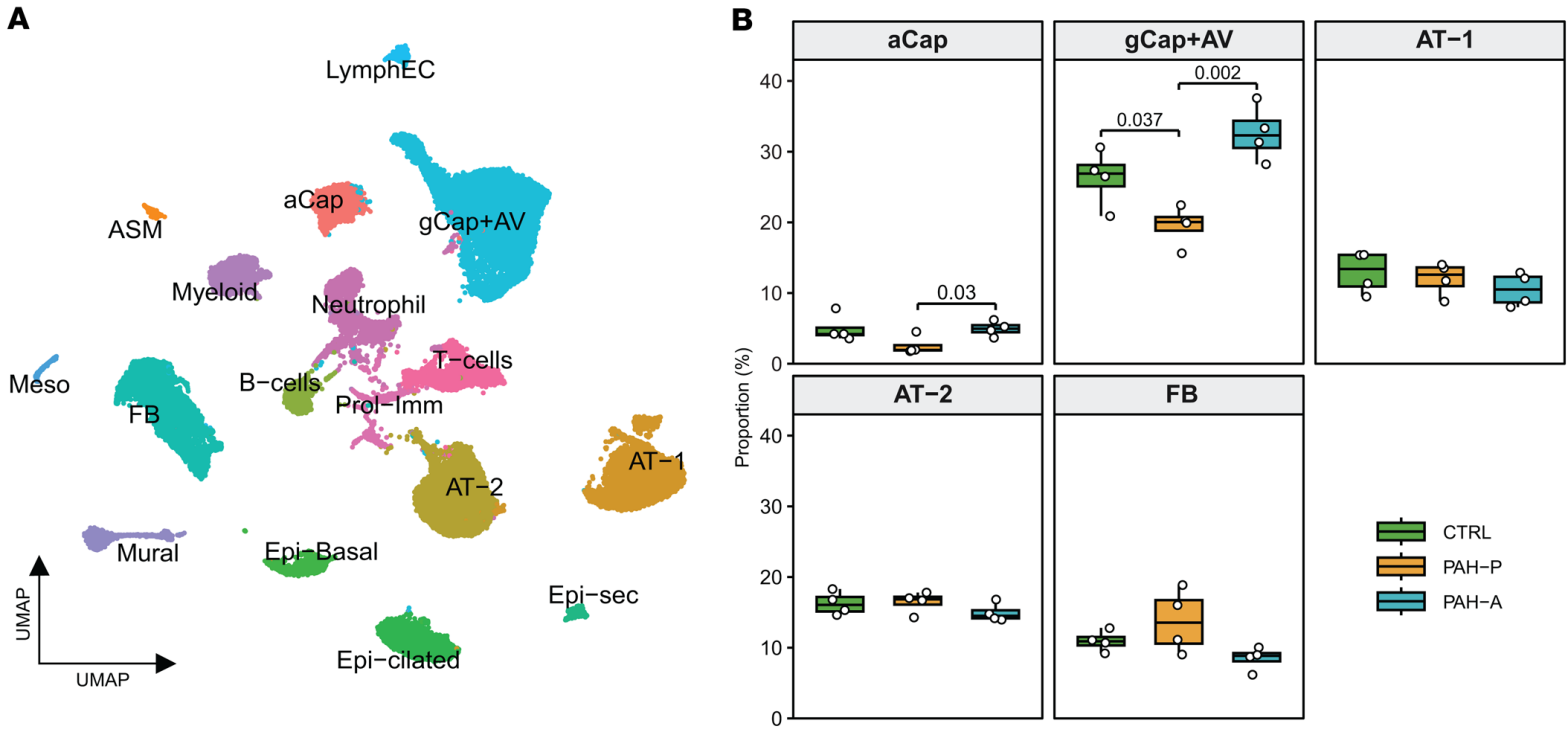
snRNA-seq reveals altered pulmonary cellular composition in pulmonary arterial hypertension that is restored by apelin analog treatment. (**A**) UMAP plot of nuclei clustered in 17 pulmonary cell types. Airway smooth muscle (ASM), myeloid immune cells (ME), mesothelial cells (Meso), endothelial aerocyte capillary cells (aCap), endothelial general capillary cells with arterial and venous cells (gCap+AV), fibroblasts (FB), lymphatic endothelial cells (LymphEC), proinflammatory immune cells (ProI-Imm), alveolar type 1 pneumocytes (AT-1), alveolar type 2 pneumocytes (AT-2), epithelial basal cells (Epi-Basal), epithelial ciliated cells (Epi-ciliated), epithelial secretory cells (Epi-sec), mural cells (Mural), B cells, T cells, and neutrophils. (**B**) Changes in relative abundance between treatment groups of major cell types aCap, gCap+AV, AT-1, AT-2, and FB shown as percentages of the entire population of lung nuclei. CTRL, control rats; PAH-P, PAH rats treated with placebo; PAH-A, PAH rats treated with apelin analog. snRNA-seq data: *n* = 4 animals per treatment condition. Data are interquartile ranges. Comparisons are done with 1-way ANOVA Tukey’s post hoc analysis for multiple comparisons.

We next performed snRNA-seq analysis of the right lower lobar lung samples as described previously (18–21). Nuclei that met quality control metrics (Supplemental Figure 8) from 4 CTRL, 4 PAH-P, and 4 PAH-A lungs were clustered on a Uniform Manifold Approximation and Projection (UMAP). Seventeen pulmonary cell types were identified by established cell markers (Figure 5A, Supplemental Figure 9A, and Supplemental Tables 2 and 3). These cell types included pulmonary endothelial cells (i.e., alveolar endothelial cells [EC], which include aerocytes [aCap], general capillary [gCap] cells, arterial and venous endothelial cells [AV cells]), and pulmonary mural cells (vascular smooth muscle and pericytes). In addition, airway smooth muscle cells (ASM), alveolar type 1 (AT-1) and alveolar type 2 (AT-2) pneumocytes, fibroblasts (FB), epithelial cells (mesothelial cells [Meso], Epi-Basal, Epi-ciliated, and Epi-sec), immune cells (myeloid, neutrophil, T cells, B cells, and proliferating immune [Prol-Imm] cells), and lymphatic endothelial cells (LymphEC) were detected. AT-1 pneumocytes, gCap+AV cells, and myeloid immune (ME) cells showed the largest number of genes with altered expression (Supplemental Table 3). The proportions of each of the 17 cell types were assessed in each disease state. The relative distribution of EC populations was the most perturbed in PAH (Figure 5B and Supplemental Table 4). The proportion of aCap cells was lower in the PAH-P group and normalized in PAH-A group while gCap+AV cell proportion was prominently reduced in PAH-P and augmented with apelin analog treatment (PAH-A) (Figure 5B). While constituting a relatively low number of cells, LymphEC were increased in the PAH-P group and were normalized by the apelin analog (Supplemental Figure 9B and Supplemental Table 4).

**Figure 6:**
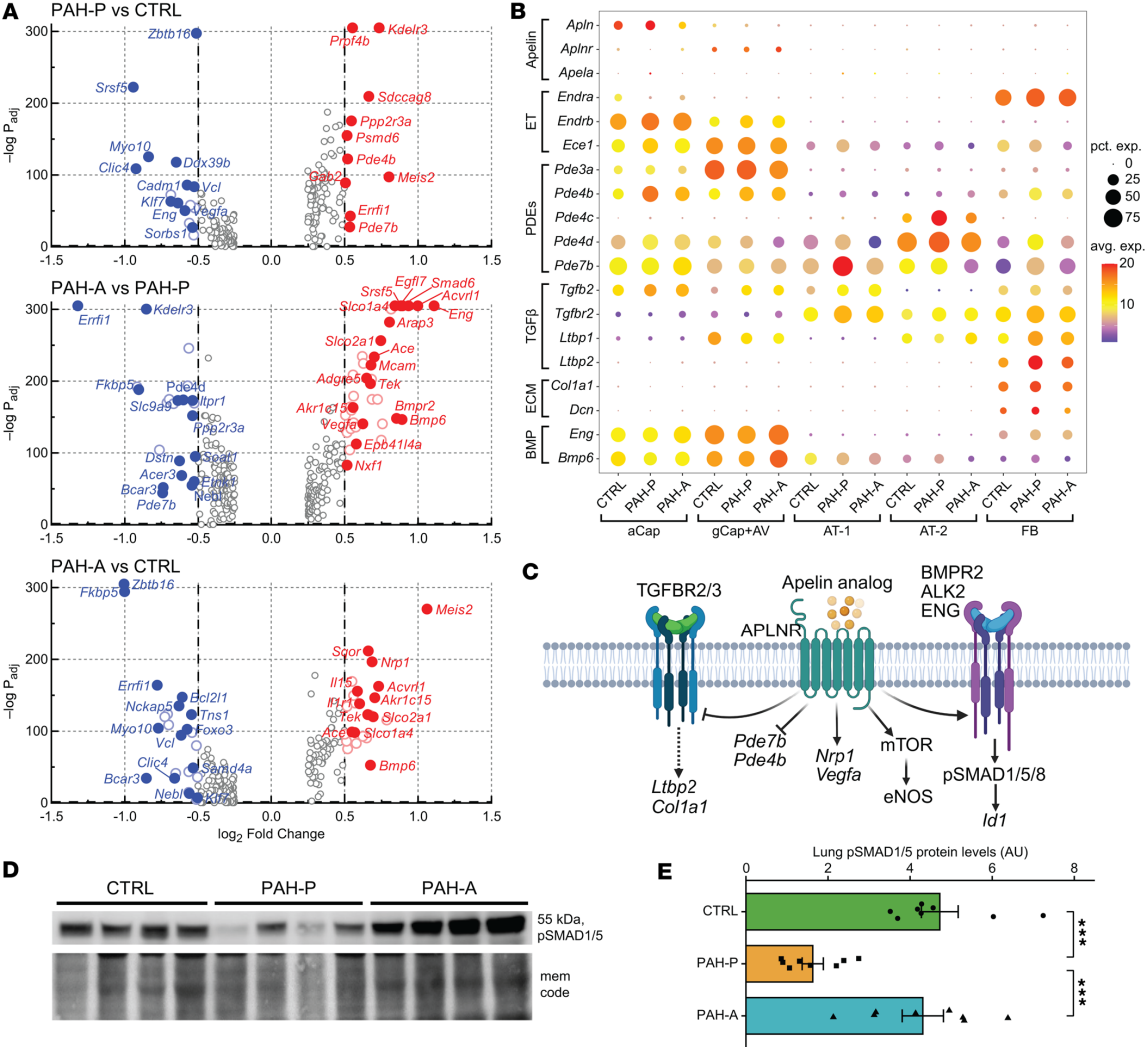
Apelin analog treatment reverses pulmonary transcriptomic remodeling and restores pSMAD1/5 signaling in pulmonary arterial hypertension. (**A**) Volcano plot of the lung pseudo-bulk transcripts for PAH-P versus CTRL, PAH-A versus PAH-P, and PAH-A versus CTRL (blue, downregulated genes; red, upregulated genes). (**B**) Dot plot of the selected genes showing average expression — purple (low), yellow (mid), and red (high) — and fraction of cell expressing (size of the dot) for major cell types (aCap, gCap+AV, AT-1, AT-2, and FB) in different treatment conditions. (**C**) Summary schematic depicting apelin mediated signaling in the lungs. (**D**) Representative Western blots for pSMAD1/5 in lung tissue. (**E**) Quantification of pSMAD1/5 levels from Western blots of lung tissue for CTRL, PAH-P, and PAH-A (*n* = 8 animals). CTRL, control rats; mem, mem code protein stain; ECM, extracellular matrix components; ET, endothelin pathway; PAH-P, PAH rats treated with placebo; PAH-A, PAH rats treated with apelin analog; PDEs, phosphodiesterases; TGF-β, transforming growth factor β pathway. snRNA-seq data: *n* = 4 animals per treatment condition. Data are shown as mean ± SD. Comparisons are done with 1-way ANOVA Tukey’s post hoc analysis for multiple comparisons. ***P < 0.001.

Pseudo-bulk analysis of the differentially expressed genes (DEGs) in all lung nuclei showed that PAH was associated with markers of increased fibrosis, inflammation, apoptosis, decreased angiogenesis, and disruption of the epithelial basal membrane (Figure 6A and Supplemental Table 5). For example, phosphodiesterases (*Pde4b*, *Pde7b*), which were upregulated in PAH-P versus CTRL are associated with endothelial-mesenchymal transition in the development of PAH (22), pulmonary fibrosis (23), and inflammation (24). Reduction in endoglin (*Eng*), signifying impaired bone morphogenetic protein receptor type 2 (BMPR2) signaling, is also consistent with PAH pathophysiology (25), while reduced angiogenesis in the PAH-P group was indicated by lowered expression of *Sorbs1* (26) and *Vegfa*. Cell adhesion molecule 1 (*Cadm1*), mediator of epithelial cell adhesion, and vinculin (*Vcl*), critical for tight junction integrity, were downregulated, suggesting disruption of epithelial basal membrane integrity (Figure 6A and Supplemental Table 5). Treatment with the apelin analog reversed these changes (Figure 6A and Supplemental Table 5). In particular, expression of *Errfi1* (ERBB receptor feedback inhibitor, known to induce apoptosis) was reduced, and expression of *Eng*, *Vegfa*, and *Cadm1* was increased in the PAH-A versus PAH-P (Figure 6A). Apelin treatment also upregulated expression of epidermal growth factor-like protein 7 (*Egfl7*), a marker of physiological angiogenesis and a number of other additional genes in the TGF-β pathway, which contribute to angiogenic homeostasis and alveolarization (activin A receptor like type 1 [*Acvrl1*]) (27) as well as facilitation of vascular quiescence (*Bmpr2* [*Bmp6*]) (Figure 6A and Supplemental Table 5) (28).

We investigated cell-specific changes in gene expression in 5 major cell types: aCap cells, gCap+AV, pneumocytes (AT-1 and AT-2), and FB (Figure 6B and Supplemental Table 3). Apelin (*Apln*) and apelin receptor (*Aplnr*) distribution was cell specific. *Apln* was mainly expressed in the aCap cells, whereas *Aplnr* was primarily expressed in the gCap+AV cells. The alternate *Aplnr* ligand, elabela (*Apela*), was virtually absent in all cell types throughout the lungs (Figure 6B and Supplemental Table 5). Western blot analysis confirmed preserved apelin levels in the PAH model, and importantly, use of the apelin analog did not downregulate expression of the *Aplnr* (Figure 6B) or lower apelin receptor protein level (Supplemental Figure 10A). We next assessed genes belonging to various pathways therapeutically linked to PAH (Figure 6B). In the endothelin pathway, endothelin receptor A (*Ednra*), while lowly expressed, was reduced in PAH-P aCaps (Figure 6B and Supplemental Table 3), and the endothelin receptor B (*Ednrb*) was upregulated in the PAH-P gCap+AV cells (Figure 6B and Supplemental Table 3). Endothelin converting enzyme (*Ece1*), which is mainly expressed in the endothelial cells (aCaps and gCap+AV), was upregulated in response to PAH and restored by treatment with the apelin analog (Figure 6B and Supplemental Table 3). For the phosphodiesterases (PDEs), *Pde4b* (aCap), *Pde4c* (AT-2), *Pde4d* (FB), and *Pde7b* (AT-1) — while upregulated in the PAH-P group — were reversed by the treatment with the apelin analog (Figure 6B and Supplemental Table 3). In contrast, the prostacyclin-related genes were relatively unaltered in our model (Supplemental Figure 10B and Supplemental Tables 2 and 4). In the context of tissue homeostasis and PAH pathophysiology, TGF-β signaling is generally pathogenic, whereas bone morphogenetic protein (BMP) signaling is protective (2, 3) (Figure 6C). In the PAH-P group, *Tgfb2* was increased in AT-1, *Tgfbr2* was increased in AT-1 and AT-2, and *Ltbp1* and *Ltbp2* levels were upregulated in FBs (Figure 6B and Supplemental Table 3). The activation of the TGF-β signaling increased expression of the ECM genes, *Col1a1* and *Dcn* (Figure 6B). The apelin analog treatment normalized *Ltbp2*, *Col1a1,* and *Dcn* levels (Figure 6B and Supplemental Table 3). Upregulation of *Tgfbr2*, *Pde7b*, *Col1a1*, and *Dcn* in FBs due to PAH suggests a transition toward a mesenchymal (fibrotic) phenotype, which was reversed by apelin analog treatment (Figure 6B and Supplemental Table 3). The BMP signaling was mainly affected in the endothelial cells (aCap and gCap+AV). PAH reduced *Eng* expression in gCap+AV cells, and the apelin analog treatment upregulated *Eng* and *Bmp6* in gCap+AV cells (Figure 6B). Examination of canonical BMPR2 signaling pathway (Figure 6C) showed that protein levels of phosphorylated SMAD1/5 complex were blunted in placebo-treated PAH lungs (Figure 6, D and E). Phosphorylated SMAD1/5 levels were recovered with apelin treatment (Figure 6, D and E), suggestive of a restoration of vascular homeostasis. Our results demonstrate that across pulmonary cell types, TGF-β signaling is upregulated in PAH, and apelin analog corrected key pathophysiological pathways, counteracted fibrotic changes, and normalized TGF-β signaling.

### Apelin analog activates vasculoprotective pathways in the lung endothelium.

**Figure 7:**
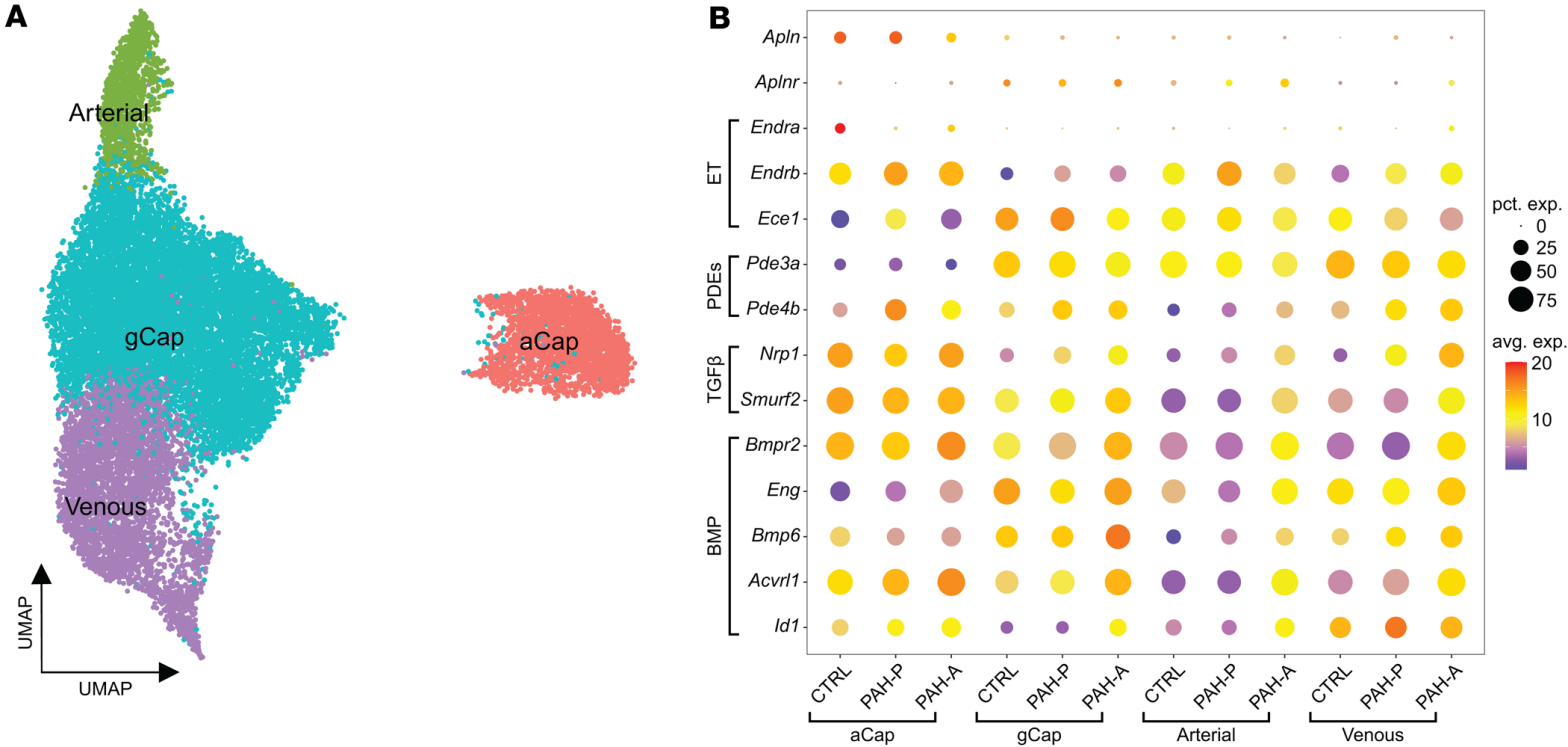
Apelin analog treatment alleviates transcriptomic remodeling across pulmonary endothelial cell subtypes in pulmonary arterial hypertension. (**A**) UMAP plot of endothelial cell subtypes: aCap (alveolar capillary endothelial cells), gCap (general capillary endothelial cells), Arterial (arterial endothelial cells), and Venous (venous endothelial cells). (**B**) Dot plot of the selected genes showing average expression — purple (low), yellow (mid), and red (high) — and fraction of cell expressing (size of the dot) for endothelial subtypes (aCap, gCap, Arterial, and Venous). ET, endothelin; PDEs, phosphodiesterases; TGF-β, transforming growth factor β pathway. snRNA-seq data: *n* = 4 animals per treatment condition.

**Figure 8:**
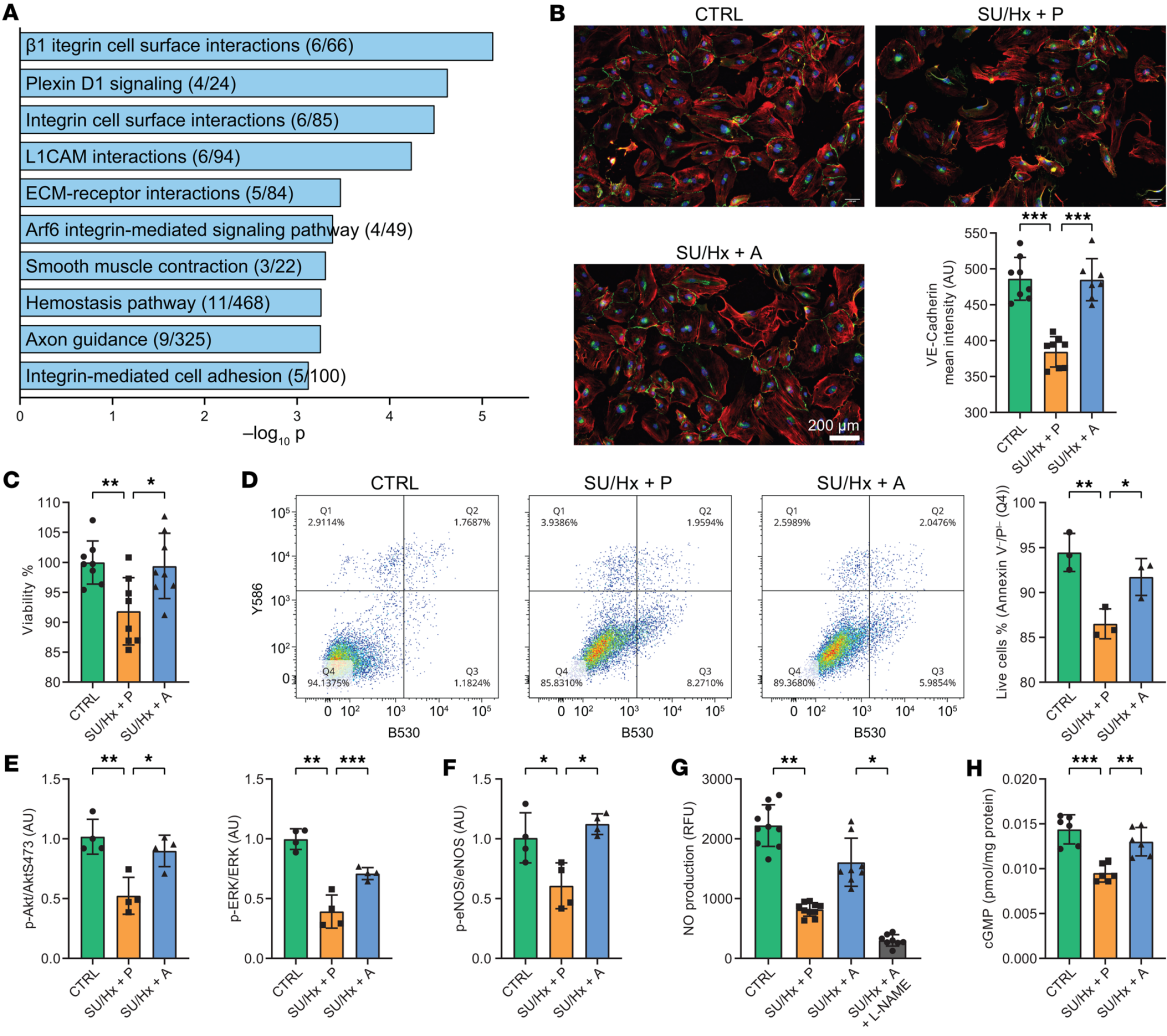
Apelin analog restores endothelial barrier integrity, viability, and nitric oxide–cGMP signaling in pulmonary arterial hypertension. (**A**) Bioplanet enrichment analyses of significantly altered pathways within endothelial cells due to PAH (PAH-P versus CTRL). (**B**) Representative images and quantification of immunofluorescent costaining of human pulmonary artery endothelial cells (HPAEC) with VE-cadherin (VE; green), F-actin (red), and DAPI (blue) (*n* = 8 replicates). Scale bar: 200 µm. (**C**) Viability assay for HPAEC across treatment groups (*n* = 8 replicates). (**D**) Annexin V (B586)/propidium iodide (Y586) dual-staining flow cytometry and quantification of live cells (Annexin V^–^/PI^–^) (*n* = 3 replicates). (**E**) p-Akt/Akt S473 and p-ERK/ERK ratio from Western blots (*n* = 4 replicates). (**F**) Quantification of p-eNOS/eNOS S1177 ratio from Western blots (*n* = 4 replicates). (**G**) Nitric oxide (NO) production in HPAECs across treatment groups (*n* = 8 replicates). (**H**) cGMP production in HPAECs across treatment groups (*n* = 8 replicates). CTRL, control rats or cells cultured in aerobic conditions; PAH-P, PAH rats treated with placebo; PAH-A, PAH rats treated with apelin analog. eNOS, endothelial nitric oxide synthase; p-eNOS, phospho eNOS; ERK, extracellular signal-regulated kinase; p-ERK, phospho-ERK; SU/Hx+P, cell cultured in sugen with hypoxia; SU/Hx+A, SU/Hx plus apelin-17 analog; RFU, relative fluorescent unit; L-NAME, NG-nitro-L-arginine methyl ester. snRNA-seq data: *n* = 4 animals per treatment condition. Data are shown as mean ± SD. Comparisons are done with 1-way ANOVA Tukey’s post hoc analysis for multiple comparisons. *P < 0.05, **P < 0.01, ***P < 0.001.

Since the lung endothelium is the major site of apelinergic activity and signaling, we performed an in-depth analysis of EC states. We resolved ECs (aCaps and gCap+AVs) into 4 EC states (aCap, gCap, arterial, and venous ECs) (Figure 7A and Supplemental Tables 2). Cell-specific gene expression showed a relatively high fraction of aCap cells (expressing *Apln*) while small fractions of all other cell types including gCap cells were expressing *Aplnr* (Figure 7B and Supplemental Table 5). Altered expression of genes from the endothelin pathway (*Ednra*, *Ednrb*, and *Ece1*) and PDEs (*Pde3a* and *Pde4b*) showed a similar pattern to the general endothelial cell types (Figure 6B and Figure 7B). Treatment with apelin analog upregulated the expression of negative regulators of TGFBR: neuropilin-1 (*Nrp1*) and SMAD specific E3 ubiquitin protein ligase 2 (*Smurf2*) (Figure 7B and Supplemental Table 6). Changes in the BMP pathways were extensive and cell-state dependent. Expression of *Bmpr2* and its coreceptor *Eng* were downregulated in PAH and normalized by apelin treatment in the gCap cells. However, in arterial and venous cells, the *Bmpr2* and *Eng* were not affected by PAH but were upregulated by apelin-analog treatment (Figure 7B). *Bmp6*, *Acvrl1*, and inhibitor of DNA binding 1 (*Id1*) exhibited the same pattern across all cell types: no change due to PAH and upregulation by apelin analog treatment (Figure 7B). An unbiased assessment of pathways performed by gene enrichment analysis using Bioplanet database (29) for ECs demonstrated alterations in integrin signaling, plexin D1 signaling, and the ECM pathway (Figure 8A and Supplemental Table 7) mainly within gCap endothelial cells (Supplemental Figure 11 and Supplemental Table 7). The ME showed upregulated changes related to the IL-2 and TNF-α signaling pathways (Supplemental Table 7).

To investigate the effects of PAH and apelin analog on the ECs at the cellular level, we established an in vitro SU/Hx model using human pulmonary artery endothelial cells (HPAECs) to simulate PAH. SU/Hx decreased cell adhesion as illustrated by VE-cadherin^+^ staining at cell borders (Figure 8B). We also performed viability assay to estimate the number of metabolically active and viable HPAECs, which suggested a reduction in response to SU/Hx (Figure 8C). To further assess the effect of SU/Hx condition on HPAEC viability, annexin V/propidium iodide (PI) dual-staining flow cytometry was performed, which showed a small but significant reduction in viable cells in response to SU/hypoxia largely prevented by the apelin analog (Figure 8D). The activation of prosurvival pathways, Akt and Erk1/2, was reduced by SU/hypoxia and restored by treatment with apelin analog (Figure 8E and Supplemental Figure 12A). Apelin itself has antiproliferative dose-dependent effects (Supplemental Figure 12B), suggesting that apelinergic protection from SU/Hx is achieved via reduction in cell death rather than via increased proliferation. Western blot analysis showed a marked increase in endothelial nitric oxide synthase (eNOS) phosphorylation (Figure 8F and Supplemental Figure 12A), resulting in increased nitric oxide (NO) production (Figure 8G) and generation of the second messenger, cGMP (Figure 8H), in response to apelin analog treatment. The increase in NO production was abolished by the eNOS specific inhibitor, L-NAME (Figure 8G). We next showed that SU/hypoxia impaired HPAEC migration and single-cell directional chemotaxis, assessed by scratch wound healing and transwell assays, respectively (Supplemental Figure 13, A and B). Apelin analog treatment partially rescued both collective migration and chemotaxis. Immunofluorescence staining at the wound edge revealed disruption of cortical F-actin stress fibers, loss of peripheral vinculin^+^ focal adhesions, and reduced pFAK (Y397) staining under SU/Hx, partially restored by apelin analog treatment (Supplemental Figure 13C). Our results demonstrate that the apelin analog normalizes vasculopathogenic signaling via BMP pathway activation in the PAH lung, preserves endothelial cells, and robustly stimulates eNOS activity and prosurvival signaling pathways, as well as cell migration in HPAECs.

### Apelin analog suppresses fibroblast and normalizes fibrosis-related changes in the lung.

**Figure 9:**
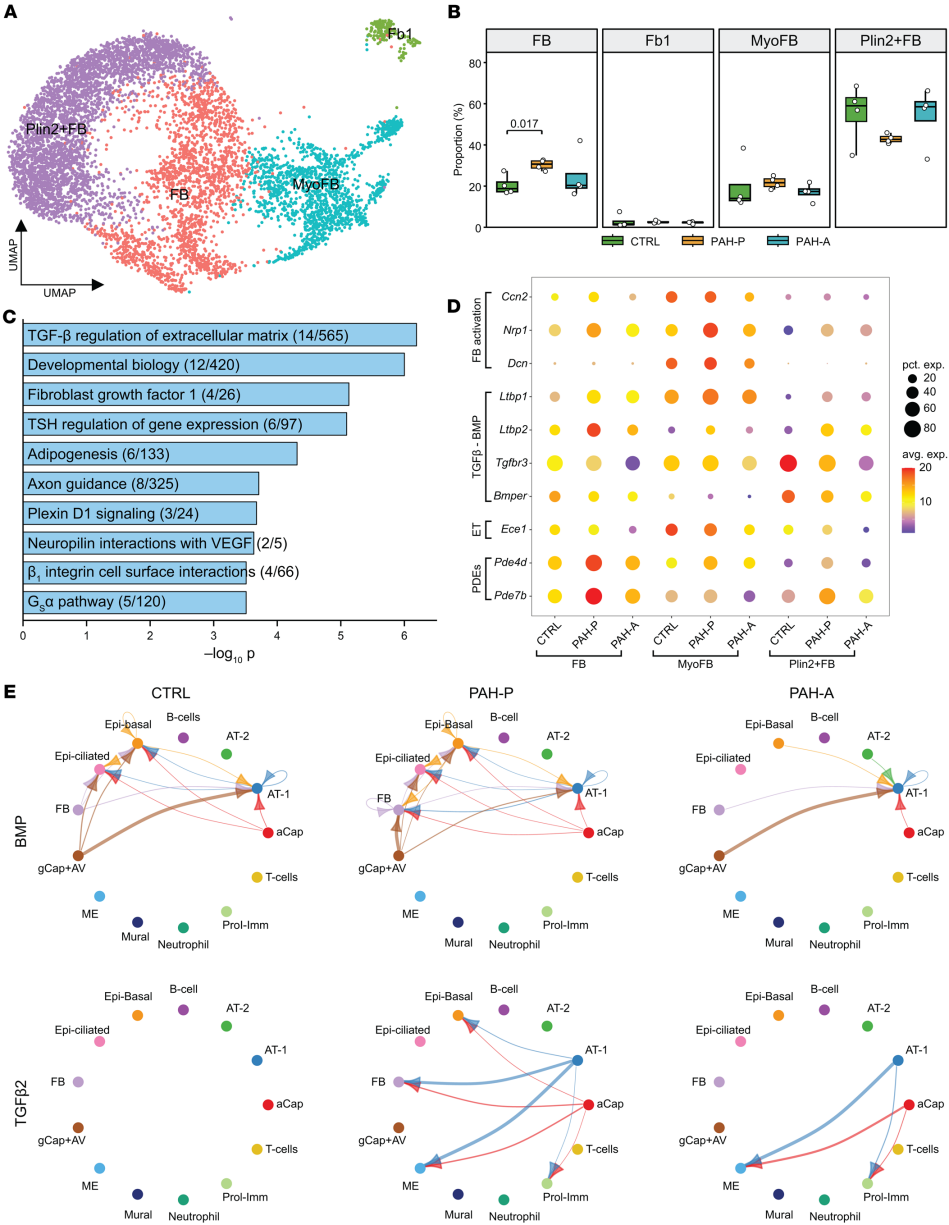
Apelin-analog treatment reverses fibroblast activation in pulmonary arterial hypertension and normalizes lung cellular communication. (**A**) UMAP plot of fibroblast cell states: canonical fibroblasts (FB), myofibroblasts (MyoFB), perilipin 2 positive lipofibroblasts (Plin2+FB), and fibroblast 1 (Fb1). (**B**) Changes in fibroblast cell-state relative abundance across treatment groups shown as percentages of the entire population of FB nuclei. (**C**) Bioplanet enrichment analyses of significantly altered pathways within fibroblast due to PAH (PAH-P versus CTRL). (**D**) Dot plot of the selected genes showing average expression — purple (low), yellow (mid), and red (high) — and fraction of cell expressing (size of the dot) for FB, MyoFB, and Plin2+FB cell states. (**E**) Communication between major pulmonary cell types *via* Bone Morphogenetic Protein (BMP) and TGF-β2. CTRL, control rats; PAH-P, PAH rats treated with placebo; PAH-A, PAH rats treated with apelin analog. FB act., FB activation; BMP, bone morphogenic protein; TGF-β, transforming growth factor β pathway; ET, endothelin; PDEs, phosphodiesterases. *n* = 4 biological replicates per treatment group. Data are interquartile ranges. Comparisons are done with 1-way ANOVA Tukey’s post hoc analysis for multiple comparisons.

Four pulmonary fibroblast cell states (FB, Fb1, MyoFB, and Plin2+FB) were annotated (Figure 9A and Supplemental Table 2). PAH was associated with an increase of the canonical FB cell state, a trend toward an increase in the activated MyoFB cell state, and a trend toward a decrease in Plin2+FB (Figure 9B and Supplemental Table 4), suggesting a population shift away from Plin2+FB toward canonical (FB) and activated myofibroblast (MyoFB) states characteristic of profibrotic pathology. The increase in the canonical FB was normalized while the Plin2+FB modestly recovered with apelin analog treatment (Figure 9B). Pathway analysis of pseudo-bulk FB DEGs in response to PAH (PAH-P versus CTRL) demonstrated enrichment of genes associated with TGF-β regulation of the ECM, FGF1 and Plexin D1 signaling, neuropilin-VEGF interactions, and hypoxia signaling (Figure 9C and Supplemental Table 7). This was corroborated by changes in the number of genes responsible for fibrotic changes: *Ccn2* (the connective tissue growth factor, which promotes transition of FB to MyoFB) (30), *Nrp1* (promotes a profibrotic phenotype in fibroblasts) (30), and *Dcn* (decorin, an ECM protein). These genes were upregulated in PAH-P group and nearly normalized with apelin analog treatment (PAH-A group) (Figure 9D and Supplemental Table 8). Expansion of the latent TGF-β binding proteins, *Ltbp1* and *Ltbp2* in FB and Plin2+FB states in response to PAH corroborated pulmonary expansion of ECM due to fibrosis. Expression levels of *Ltbp1* and *Ltbp2* were decreased by apelin analog (Figure 9D). Changes in the TGF-β and BMP pathways were limited to *Tgfbr3* and *Bmper*. The level of these transcripts, while unaffected by PAH, was downregulated in response to apelin analog treatment in FB and Plin2+FB cell states (Figure 9D). *Ece1* followed a similar pattern, whereas PDEs (*Pde4d* and *Pde7b*) were upregulated in PAH-P and normalized in the PAH-A group (Figure 9D). Taken together, PAH was associated with pulmonary fibrosis, and apelin treatment reduced fibrosis by reducing the expression of the coreceptor (*Tgfbr3*), repressor of BMPR signaling (*Bmper*), and *Ece1*.

Expression levels of receptors and ligands were examined using CellChat to infer cell-cell communications. The bone morphogenic protein signaling pathway (agonist/ligand: BMP6, receptors: BMPR1A+BMPR2) showed that gCap+AV cells mainly exert influence on FB in the PAH-P group to counteract fibrotic expansion. TGF-β signaling (agonist/ligand: TGF-β2, receptors: TGF-βR1+TGF-βR2) was quiescent in control; however, in the PAH-P, aCaps and AT-1 exerted profibrotic influence on FB and antiinflammatory influence on ME (Figure 9E). Reduction of the fibrosis with apelin analog treatment was accompanied by the absence of aCap-FB and AT-1-FB communications, but antiinflammatory influence on ME persisted (Figure 9E). Taken together, apelin analog treatment is associated with normalized fibrosis-related intercellular communication.

### snRNA-seq reveals rescue of molecular perturbations in PAH-induced RV failure.

**Figure 10:**
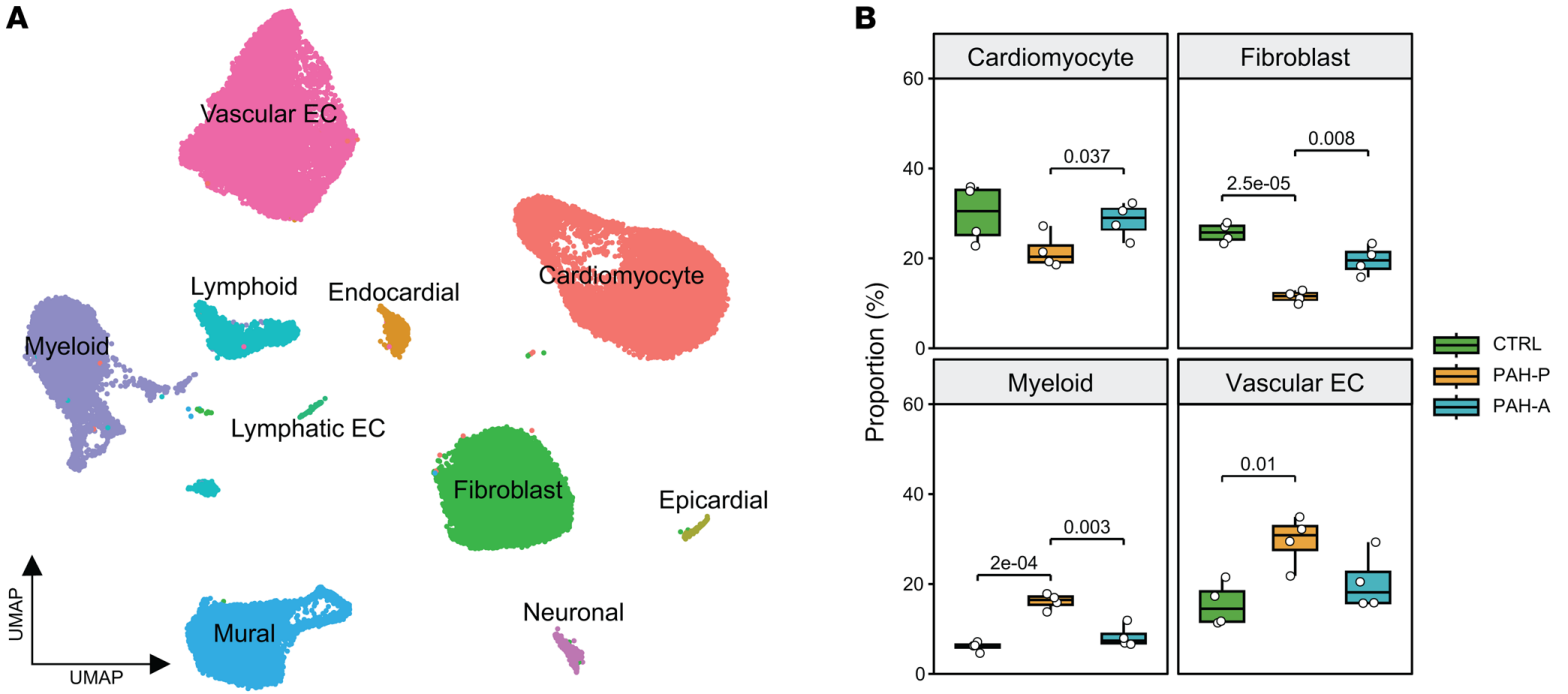
snRNA-seq reveals altered right ventricular cellular composition in pulmonary arterial hypertension. (**A**) Global UMAP plot of nuclei showing 11 cell types in the right ventricle (RV): ventricular cardiomyocytes (Cardiomyocyte-V), vascular endothelial cells (Vascular EC), endocardial cells (Endocardial), epicardial cells (Epicardial), fibroblasts, lymphatic endothelial cells (Lymphatic EC), lymphoid cells (Lymphoid), memory B cells, mural cells (Mural), myeloid cells (Myeloid), and neuronal cells (Neuronal). (**B**) Changes in relative abundance of major cell types (Cardiomyocyte-V, Fibroblast, Myeloid, and Vascular EC) across treatment groups shown as percentages of the entire population of RV nuclei. snRNA-seq data: *n* = 4 per treatment group. Data are interquartile ranges. Comparisons are done with 1-way ANOVA Tukey’s post hoc analysis for multiple comparisons.

**Figure 11:**
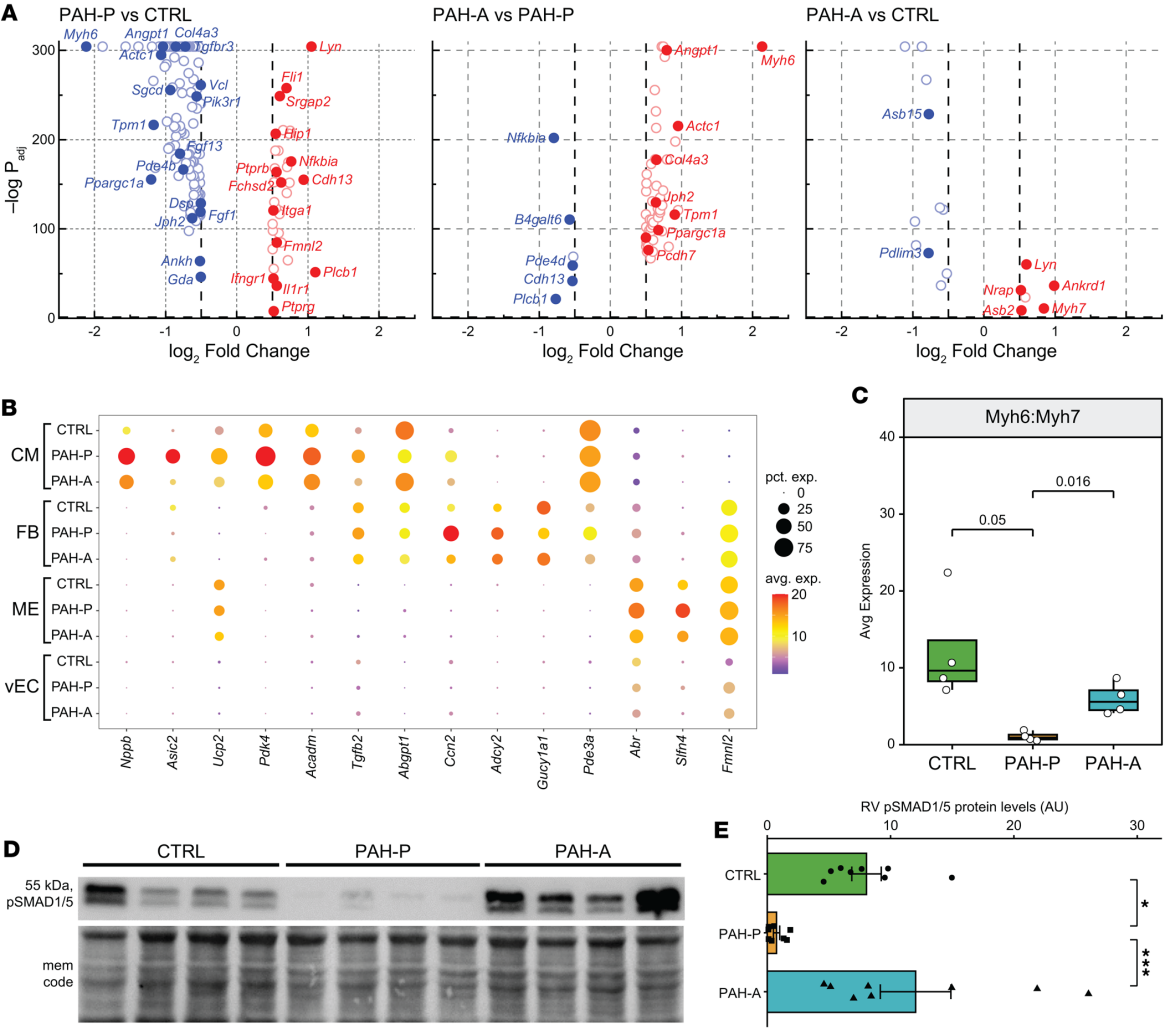
Apelin analog treatment reverses right ventricular transcriptomic remodeling and restores pSMAD1/5 signaling in pulmonary arterial hypertension. (**A**) Volcano plot of the RV pseudo-bulk transcripts for PAH-P versus CTRL, PAH-A versus PAH-P, and PAH-A versus CTRL (blue, downregulated genes; red, upregulated genes). (**B**) Dot plot of the selected genes showing average expression — purple (low), yellow (mid), and red (high) — and fraction of cell expressing (size of the dot) for major cell types (cardiomyocytes, CM; fibroblasts, FB; myeloid, ME; vascular endothelial cells, vEC) in different treatment conditions. (**C**) Ratio of cardiomyocyte pseudo-bulk expression of the myosin heavy chain 6 (Myh6 or MyHα) to myosin heavy chain 7 (Myh7 or MyHβ) (Myh6:Myh7) across treatment groups. Data are interquartile ranges. (**D**) Representative Western blots for pSMAD1/5 in RV tissue. (**E**) Quantification of pSMAD1/5 levels from Western blots of RV tissue (*n* = 8 animals; mean ±SD). CTRL, control rats; mem, mem code protein stain; PAH-P, PAH rats treated with placebo; PAH-A, PAH rats treated with apelin analog. snRNA-seq data: *n* = 4 per treatment group. Comparisons are done with 1-way ANOVA Tukey’s post hoc analysis for multiple comparisons. *P < 0.05, ***P < 0.001.

We next examined the nuclei isolated from transmural RV free wall in CTRL, PAH-P, and PAH-A rats as described previously (31, 32). Following snRNA-seq, preprocessing, and quality control filtering, the data were integrated using Harmony (Supplemental Figure 14A). Eight major cardiac cell types were identified: ventricular cardiomyocytes (CM); vascular endothelial cells (EC) including lymphatic EC, endocardial and epicardial cells; FB; pericytes and smooth muscle cells (mural cells); myeloid and lymphoid (immune cells); and neuronal cells (Figure 10A, Supplemental Figure 14B, and Supplemental Table 9). The CMs and FBs showed the largest number of genes with altered expression (Supplemental Table 10). The relative abundance analysis showed a lowered count of CMs and FBs while the myeloid cell and vascular EC fractions increased in PAH-P rats. These changes were reversed in the RVs from PAH rats treated with the apelin analog (Figure 10B and Supplemental Table 11). In the RV, *Apln* is predominantly expressed in CMs and vascular ECs, which was reduced with PAH and partially restored with apelin analog, whereas *Aplnr* was mainly expressed in vascular ECs and *Apela* expression is negligible in all major RV cell types (Supplemental Figure 15A). Western blot analysis confirmed decreased apelin levels in the RV in PAH-P group partly restored by apelin treatment with maintained apelin receptor levels (Supplemental Figure 15B). The examination of DEGs of pseudo-bulk RNA-seq of all RV nuclei revealed heightened inflammatory (*Nfkbia*, *Ifngr1*, *Il1r1*), migratory, and fibrotic mediators (*Plcb1*) in PAH-P compared with CTRL. PAH was also associated with downregulated genes involved in sarcomere structural support (*Tpm1*, *Actc1*); cytoskeleton-plasma membrane-ECM linkers (*Sgcd*, sarcoglycan δ; *Vcl*, vinculin); vasculoprotection (angiopoietin 1, *Angpt1*), which suppress inflammation and preserve endothelial integrity; and *Ppargc1a,* a stimulator of mitochondrial biogenesis (Figure 11A and Supplemental Table 12). Apelin treatment upregulated a stimulator of mitochondrial metabolism (*Ppargc1a*)*,* sarcomere structural support genes (*Tpm1*, *Actc1*), and the vasculoprotective factor *Angpt1* (Figure 11A), while also reducing inflammatory markers *Nfkbia*, migratory marker *Plcb1*, and vasoconstriction associated gene *Pde4d* (Figure 11A).

In CMs, heart failure markers *Nppb* (natriuretic peptide B) and *Asic2* (acid sensing ion channel subunit 2) were upregulated in PAH-P (Figure 11B and Supplemental Tables 10 and 12), while *Myh6*:*Myh7* expression ratio was reduced (Figure 11C). Metabolic inefficiency in CMs was evident from (a) uncoupling due to upregulation of *Ucp2*, uncoupling protein 2, and (b) shifting oxidation from glucose to fatty acid (increased expression of *Pdk4*, pyruvate dehydrogenase kinase 4, and *Acadm*, Acyl-CoA dehydrogenase medium chain) (Figure 11B). The increase in *Tgfb2* (transforming growth factor β2) expression and decrease in *Angpt1* expression (Figure 11B) suggest profibrotic and antiangiogenic signals in CM, respectively. Apelin analog treatment reversed most of the changes induced by PAH (Figure 11B). In FBs, PAH was profibrotic: (a) increased expression of profibrotic factor *Ccn2* (Figure 11B and Supplemental Table 12) and (b) shift from cGMP to cAMP signaling as evidenced by upregulation of (*Adcy2*, adenylate cyclase 2; cAMP production) and downregulation of *Gucy1a1,* (guanylate cyclase 1 soluble subunit α1; cGMP production). This shift was accompanied by increase in *Pde3a* expression. Apelin analog treatment reversed all these changes except for changes in *Adcy2* (Figure 11B). In ME, PAH displayed an antiinflammatory profile as illustrated by upregulation of negative regulator of inflammation (*Abr*, ABR activator of RhoGEF and GTPase) and inhibitor of T cell proliferation (*Slfn4*, schlafen 4) (Figure 11B). Apelin analog reversed both of those changes (Figure 11B). In contrast to the lungs, vascular ECs in the RV showed relatively minor changes in the context of PAH (Supplemental Table 10). Upregulation of formin like 2 (*Fmnl2*), a paracrine proangiogenic factor, correlated with EC expansion in the RV in response to PAH (Figure 10B and Figure 11B). Loss of BMPR2 signaling, a key pathogenic factor in PAH, reduces phosphorylation of the downstream protective effector, SMAD1/5/8 (33, 34). Western blots showed reduced phosphorylation of SMAD1/5 in response to PAH and treatment with apelin analog restored phosphorylated SMAD1/5 levels (Figure 11, D and E). Our data illustrate that PAH is associated with structural, molecular, and metabolic changes in the failing RV, including fibrosis, and mitochondrial uncoupling accompanied by a shift from glucose to fatty acid oxidation. Treatment with apelin analog largely rescued these changes.

### Remodeling of the RV cardiomyocytes in PAH is ameliorated by apelin analog.

**Figure 12:**
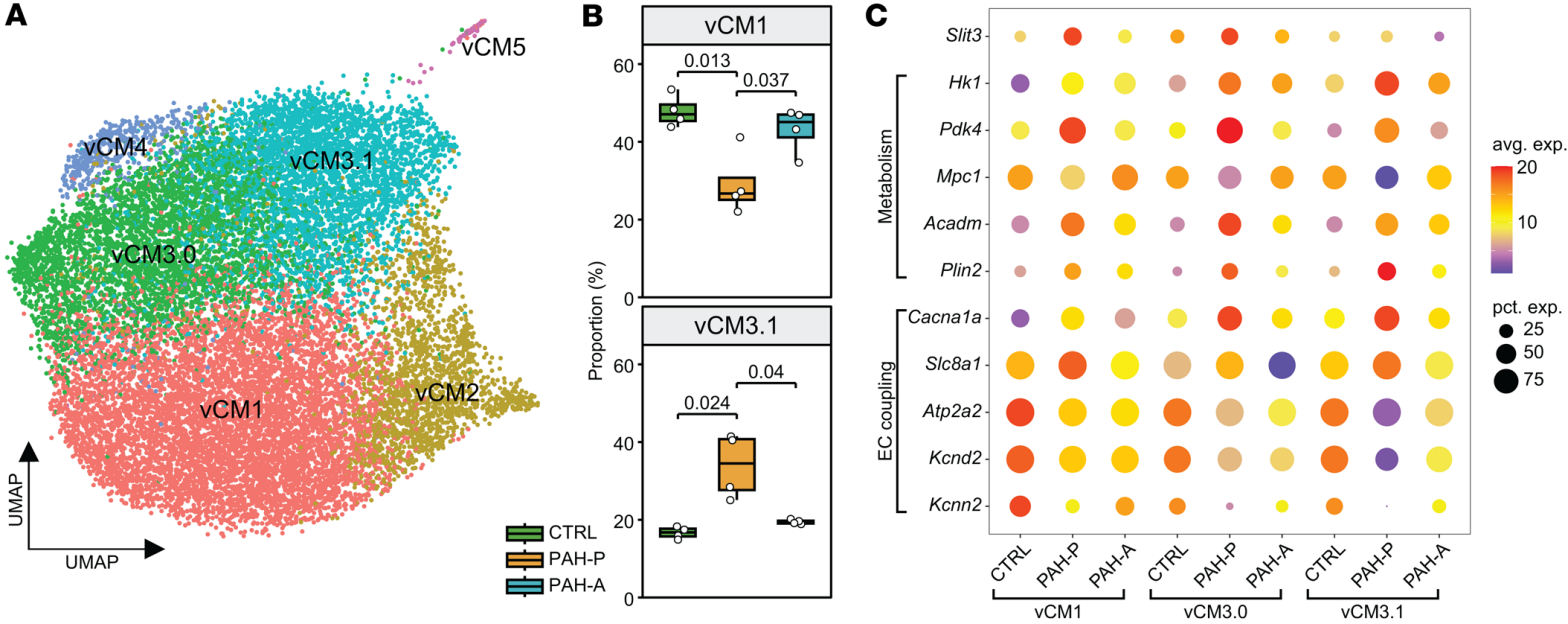
Apelin analog treatment reverses adverse cardiomyocyte state–specific remodeling in the right ventricle in pulmonary arterial hypertension. (**A**) UMAP plot of 6 RV cardiomyocyte cell states: vCM1, vCM2, vCM3.0, vCM3.1, vCM4, vCM5. (**B**) Changes in relative abundance of vCM1 and vCM3.1 cardiomyocyte cell state across treatment groups shown as percentages of the entire population of cardiomyocyte nuclei. (**C**) Dot plot of the selected genes showing average expression — purple (low), yellow (mid), and red (high) — and fraction of cell expressing (size of the dot) for vCM1, vCM3.0, and vCM3.1 cell states. EC coupling, excitation-contraction coupling; CTRL, control rats; PAH-P, PAH rats treated with placebo; PAH-A, PAH rats treated with apelin analog. Data are interquartile ranges. Comparisons are done with 1-way ANOVA Tukey’s post hoc analysis for multiple comparisons. snRNA-seq data: *n* = 4 animals per treatment condition.

Six cardiomyocyte states (vCM) were identified in the normal RV: vCM1-5 and vCM3.1 (Figure 12A and Supplemental Table 9) (31, 32). PAH RVs had significantly fewer vCM1 cells with corresponding increase in vCM3.1 fraction (Figure 12B and Supplemental Figure 16), which is involved in cell-cell or cell-ECM binding in response to mechanical stress (Supplemental Figure 17A). Other vCM states remained unchanged (Supplemental Figure 16). Pathway analysis of pseudo-bulk data for CMs in response to PAH demonstrated enrichment of transcripts related to dilated cardiomyopathy, cell-cell communication, cell-ECM interactions, and arrhythmogenic right ventricular cardiomyopathy (Supplemental Figure 17B and Supplemental Table 13). Consistent with high RV pressures in PAH (Figure 2, C and D), *Slit3*, a marker of pressure overload and hypertrophy (35), was increased in vCM1 and vCM3.0 (Figure 12C and Supplemental Table 14). PAH also led to remodeling of the cardiac metabolism typical of heart failure in vCM3.0 and vCM3.1 (Figure 12C and Supplemental Table 14): (a) increase in glycolysis (upregulation of *Hk1*, hexokinase 1); (b) reduction in pyruvate oxidation (upregulation of an inhibitor of pyruvate dehydrogenase, *Pdk4* (pyruvate dehydrogenase kinase 4) accompanied by the reduction in *Mpc1*, mitochondrial pyruvate carrier 1); (c) increase in fatty acid oxidation (upregulation of *Acadm*, acyl-CoA dehydrogenase medium chain, expression in vCM1, vCM3.0, and vCM3.1); and (d) increased lipid accumulation (upregulation of intracellular lipid storage protein, *Plin2*, perilipin 2). Apelin analog treatment mostly normalized changes induced by PAH in metabolism-related transcripts (Figure 12C). In addition to changes in metabolism, PAH also resulted in remodeling of excitation-contraction coupling similar to changes seen in heart failure. Expression of *Cacna1a* (Cav 2.1, P/Q voltage-gated Ca^2+^ channel) was increased in vCM1, vCM3.0, and vCM3.1. *Slc8a1* (type 1 Na^+^/Ca^2+^ exchanger) was increased predominantly in vCM3.0, while *Atp2a2* (sarcoplasmic/endoplasmic reticulum Ca^2+^ ATPase 2) expression was significantly reduced in vCM3.0 and vCM3.1. Expression of K^+^ channels, *Kcnd2* (Kv4.2) and *Kcnn2* (KCa2.2), decreased in vCM1, vCM3.0, and vCM3.1 (Figure 12C). Changes to excitation-contraction coupling gene transcripts were normalized by apelin analog treatment (Figure 12C).

**Figure 13:**
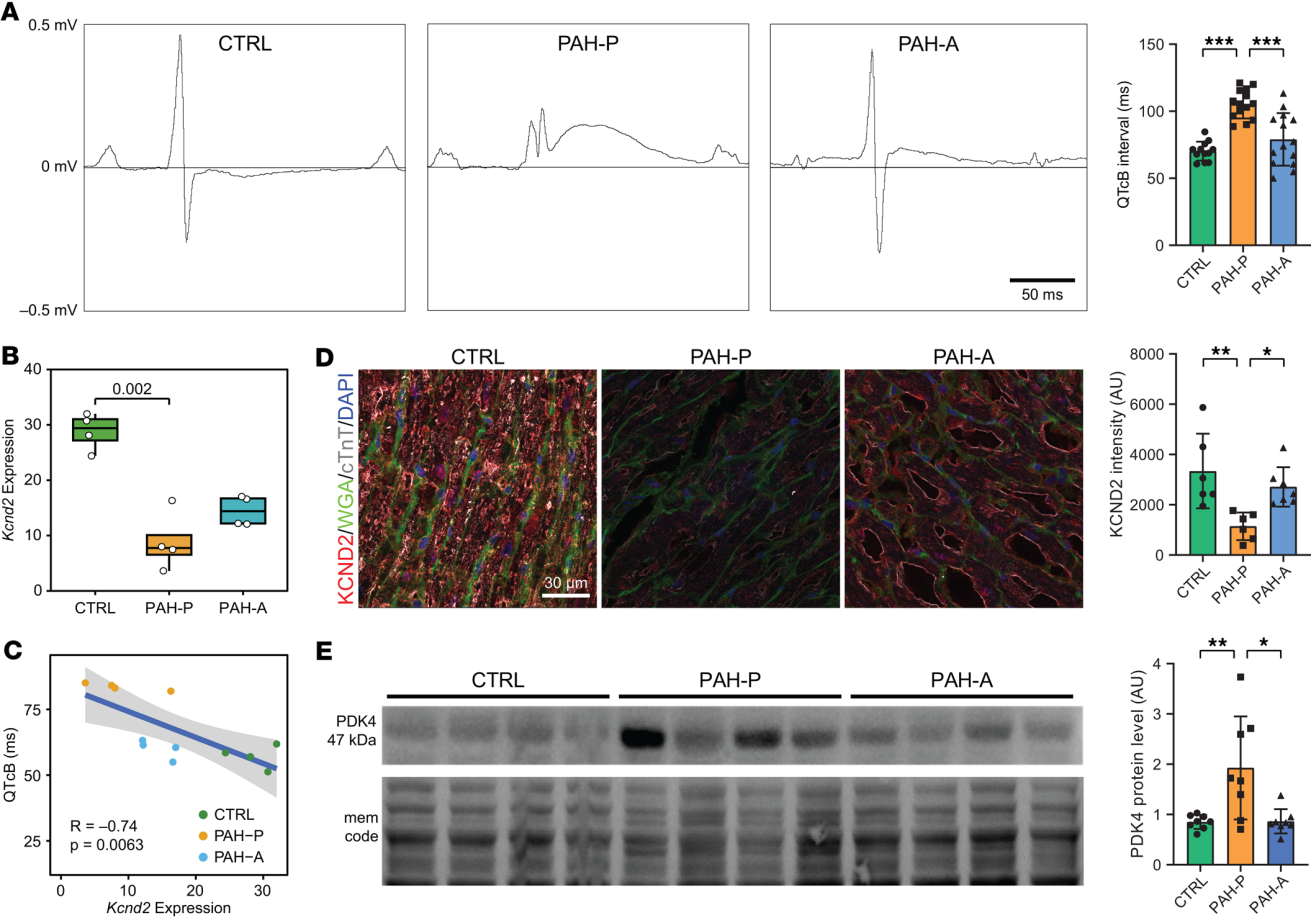
Apelin analog treatment reverses electrical and metabolic remodeling of right ventricular cardiomyocytes in pulmonary arterial hypertension. (**A**) Representative electrocardiograms (Lead I) and corrected QT intervals (Bazett correction). (**B**) Expression of K^+^ ion channel *Kcnd2* in RV cardiomyocytes. (**C**) Inverse correlation between QTc interval (Bazett) and *Kcnd2* expression in RV cardiomyocytes. (**D**) Representative immunofluorescence staining of RV tissue sections. Merged view: KCND2 (red), wheat germ agglutinin (WGA) (green), cardiac troponin T (cTnT) (gray), and DAPI (blue) (left); scale bar: 30 µm. Quantification of KCND2 protein levels in the cardiomyocyte area (right). (**E**) Representative Western blot of PDK4 levels across treatment groups normalized to memcode (left). Quantification of protein levels of PDK4 in Western blots of RV tissue (right). CTRL, control rats; PAH-P, PAH rats treated with placebo; PAH-A, PAH rats treated with apelin analog. *n* = 12–14 animals for ECG measurements, *n* = 8 for immunoblotting. Data are shown as mean ± SD (**A**, **D**, and **E**) and IQR (**B**). Comparisons are done with 1-way ANOVA Tukey’s post hoc analysis for multiple comparisons. *P < 0.05, **P < 0.01, ***P < 0.001.

To determine the effects of the apelin analog in electrical remodeling, we performed Lead I electrocardiogram (ECG) and showed that the electrical profile of untreated PAH rats was characterized by ST-segment elevation, a prominent T wave, and prolonged corrected QT interval with incomplete right bundle branch block (Figure 13A). In apelin-treated PAH rats, ST-segment elevation was diminished, QT intervals were nearly normal, and conduction block was absent (Figure 13A and Supplemental Figure 18A); RR and PR intervals were unaffected (Supplemental Figure 18, B and C). Expression of *Kcnd2* (Figure 13B) and *Kcnn2* (Supplemental Figure 18D) was reduced in the PAH group, and QT interval prolongation negatively correlated with *Kcnd2* levels (Figure 13C and Supplemental Figure 18, E and F). Immunofluorescence staining with confocal imaging revealed the RV cardiomyocyte membrane demarcated with Kv4.2 (*Kcnd2*) staining, which was downregulated in PAH and restored by treatment with apelin analog (Figure 13D). Overall electrical and metabolic disruptions were reversed by the apelin analog treatment (Figure 12C and Figure 13, A–D). Additionally, we confirmed PDK4 protein levels nearly doubled in the PAH-P group (Figure 13E), likely reflecting impaired glucose oxidation and promotion of glycolysis. Overall, these results highlight profound metabolic abnormalities and repolarization deficiencies in the failing RV induced by PAH, all of which were fully or partially reversed by apelin analog treatment.

### Reversal of RV profibrotic and inflammatory signatures in response to apelin analog.

**Figure 14:**
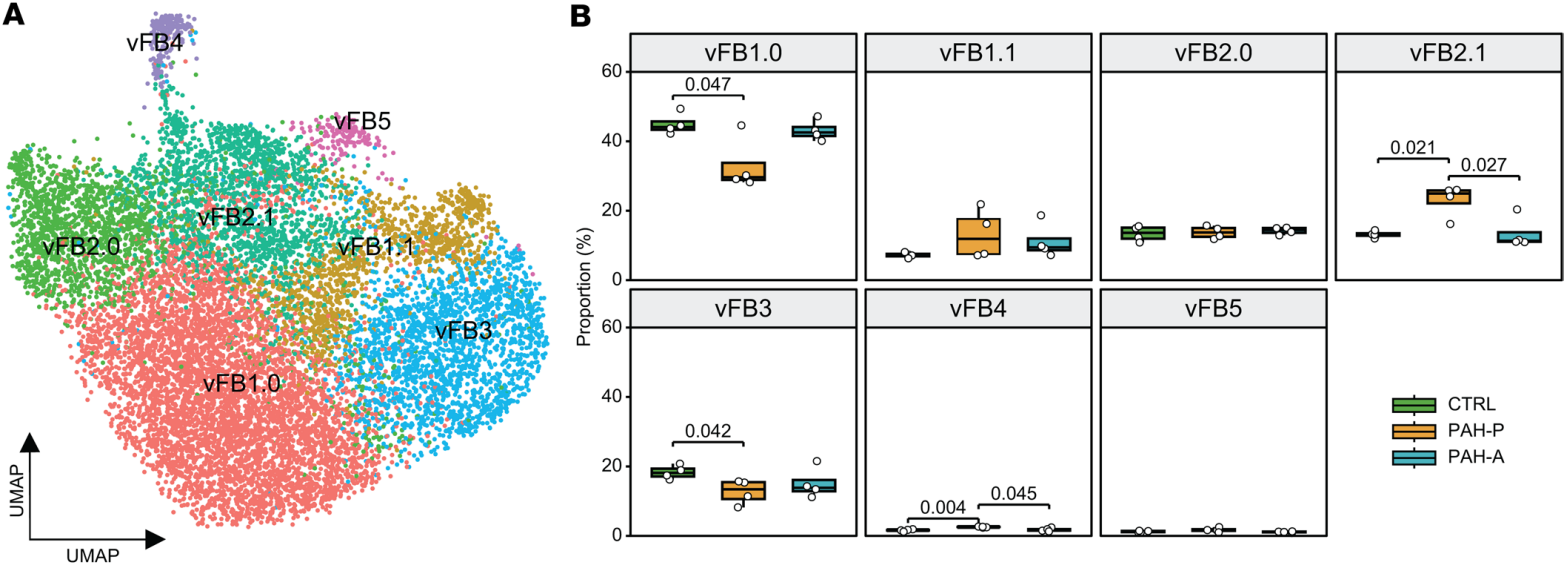
snRNA-seq reveals right ventricular fibroblast cell state remodeling in pulmonary arterial hypertension that is normalized by apelin analog treatment. (**A**) UMAP plot of 7 RV fibroblast cell states: vFB1.0, vFB1.1, vFB2.0, vFB2.1, vFB3, vFB4, and vFB5. (**B**) Changes in relative abundance of fibroblast cell states across treatment groups shown as percentages of the entire population of FB nuclei. CTRL, control rats; PAH-P, PAH rats treated with placebo; PAH-A, PAH rats treated with apelin analog. Data are shown as IQR. Comparisons are done with 1-way ANOVA Tukey’s post hoc analysis for multiple comparisons. Comparisons are done with 1-way ANOVA Tukey’s post hoc analysis for multiple comparisons. snRNA-seq data: *n* = 4 animals per treatment condition.

**Figure 15:**
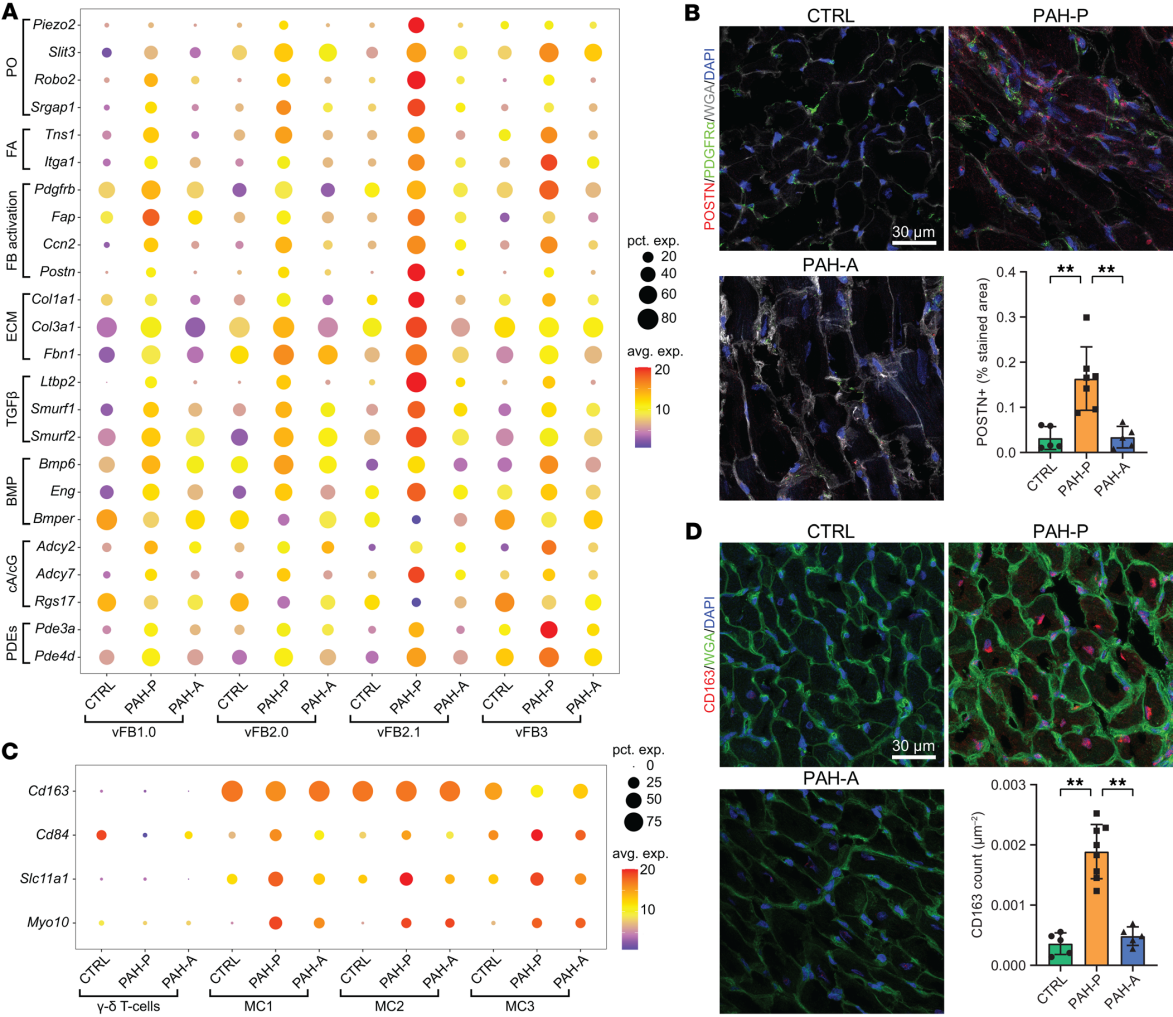
Apelin analog treatment normalizes fibroblast activation and myeloid cell states in the pressure-overloaded right ventricle in pulmonary arterial hypertension. (**A**) Dot plot of the selected genes showing average expression — purple (low), yellow (mid), and red (high) — and fraction of cell expressing (size of the dot) for vFB1.0, vFB2.0, vFB2.1, and vFB3 cell states. (**B**) Representative images and quantification of immunofluorescence staining for activated fibroblasts (vFB2.1) in the RV based on costaining for periostin (POSTN, red) and platelet derived grown factor receptor α (PDGFRα; green). Scale bar: 30 µm. (**C**) Dot plot of the selected genes showing average expression — purple (low), yellow (mid), and red (high) — and fraction of cell expressing (size of the dot) for γ-δ T cells and monocytes (MC1, MC2, and MC3). (**D**) Representative images and quantification of immuno-fluorescence staining for myeloid marker CD163 (red); WGA (green) and DAPI (blue). Scale bar: 30 µm. CTRL, control rats; PAH-P, PAH rats treated with placebo; PAH-A, PAH rats treated with apelin analog. PO, pressure overload; FA, focal adhesion; FB act., fibroblast activation; ECM, extracellular matrix components; TGF-β, transforming growth factor β pathway; BMP, bone morphogenic protein; cA/cG, cAMP/cGMP production; PDEs, phosphodiesterases. *n* = 5–8 tissue sections. Data are shown as mean ± SD. Comparisons are done with 1-way ANOVA Tukey’s post hoc analysis for multiple comparisons. **P < 0.01. snRNA-seq data: *n* = 4 animals per treatment condition.

Given the increased tissue fibrosis observed in pressure-overloaded RV, we explored the cellular and molecular basis for the profibrotic changes within the RV seen in our PAH model. Seven FB states — vFB1–5, vFB1.1, and vFB2.1 (Figure 14A and Supplemental Table 9) — were identified (31, 32). Cell state analysis revealed a PAH-associated increase in vFB2.1 state and a corresponding reduction in canonical vFB1 with the other states remaining unchanged (Figure 14B and Supplemental Table 11). Functional elucidation of FB cell states revealed that vFB2.1 executes ECM synthesis and glycosaminoglycan indicative of a secretory role (Supplemental Figure 19A); the expansion of vFB2.1 was alleviated with apelin analog treatment. Pathway analysis of pseudo-bulk data for FBs with Bioplanet (29) demonstrated that, in response to PAH, transcripts related to TGF-β regulation of ECM, focal adhesion, PDGF (platelet-derived growth factor) signaling, ECM-receptor interactions, collagen biosynthesis, and integrin-related pathways were enriched (Supplemental Figure 19B and Supplemental Table 13). Cell-state specific changes in gene expression showed that PAH upregulated genes related to pressure overload. *Piezo2* (mechanosensitive channel), *Slit3*, and *Robo2* (roundabout guidance receptor 2), components of Slit3/Robo axis involved in pressure overload (35), and its downstream target *Srgap1* (SLIT-ROBO Rho GTPase activating protein 1) were upregulated (Figure 15A and Supplemental Table 15). A similar response to PAH pressure-overload occurred in transcripts related to focal adhesion (*Tns1* and *Itga1*). These responses were associated with profibrotic transcripts (*Fap*, *Ccn2*, and *Pdgfrb*, platelet-derived growth factor receptor β) and ECM-related transcripts (*Postn*, *Col1a1*, *Col3a1*, and *Fbn1*, fibrillin 1) (Figure 15A). PAH also resulted in upregulation of TGF-β and BMP signaling. Upregulation of *Ltbp2* suggested activation of TGF-β pathway. However, this activation was limited by an increased expression of SMAD-specific ubiquitin ligases (*Smurf1* and *Smurf2*) (Figure 15A). Concordant upregulation of *Bmp6* and *Eng* coupled with downregulation of *Bmper,* an extracellular BMP antagonist, is consistent with enhanced BMP pathway activity (Figure 15A). PAH was associated with upregulation of transcripts associated with cAMP and cGMP production. Adenylate cyclases were upregulated (*Adcy2* in vFB3 and *Adcy7* in vFB2.1), whereas inhibitor of G-protein signaling, Rgs17, was downregulated mainly in vFB2.1. Increased cyclic nucleotide production was associated with increased expression of PDEs (*Pde3a* and *Pde4d*). These changes were normalized by treatment with the apelin analog (Figure 15A). Overall, all FB states responded similarly to PAH and apelin-analog treatment with the most prominent changes seen in vFB2.1 state (Figure 15A). Periostin^+^ staining was increased in PAH-P RV and was normalized by apelin analog treatment confirming the profibrotic changes in the RV tissue (Figure 15B).

We next examined the close association between the immune system, tissue injury, and fibrosis (36, 37). Categorization of immune cell states showed 17 cell states (Supplemental Figure 20A and Supplemental Table 16). In PAH, expression of *Cd84* (suppressor of T and B Cell activation) was increased in MC1 (monocytes) (Figure 15C and Supplemental Table 16). *Slc11a1* (H^+^-coupled divalent metal ion transporter) that augments activation of lymphocytes was increased in PAH-P monocytes (MC1-MC3). *Myo10* (myosin X) responsible for filopodial motility was significantly upregulated in MC1 within the PAH-P group (Figure 15C). We stained RV sections for the macrophage marker, CD163, and found an increase in positive cells in PAH, which was resolved with apelin analog treatment (Figure 15D) consistent with the changes observed in global myeloid immune cell count (Supplemental Table 11). Overall, these changes suggest that PAH resulted in mild activation of monocytes and inflammation, and most of those changes were reverted by the apelin analog treatment (Figure 15, C and D). Mural cells (pericytes and vascular smooth muscle cells) showed no discernable changes in cell distribution and gene expression (Supplemental Figure 20B). These results highlight the crucial role of myofibroblast and myeloid cell activation in development of fibrosis due to pressure overload. PAH-driven myofibroblast expansion, myeloid cell activation, and fibrosis were normalized by apelin analog treatment.

## Discussion

In PAH, physiological lung endothelial signaling and angiogenic processes are dysfunctional leading to a proproliferative, fibrogenic, and inflammatory vascular milieu, followed by RV maladaptive remodeling and dysfunction. The SU/Hx rat model recapitulates human PAH pathogenesis with partial BMPR2 inhibition, vascular injury closely resembling human PAH vasculopathy (16, 38, 39), and subsequent RV remodeling. In the lungs, we observed neointimal formations leading to plexiform lesions, loss of the perfusing pulmonary vasculature resulting in high pulmonary arterial pressures. In the heart, PAH was associated with pronounced RV dilation, paradoxical septal movement, and ventricular interdependence. The latter, combined with impaired LV preload, led to reduced LV stroke volume and elevated venous pressure, which in turn diminished renal perfusion and culminated in cardiorenal syndrome, a key determinant of outcomes in patients with RV failure (40, 41). We used a potentially novel apelin analog therapy and demonstrated its ability (a) to rebalance TGF-β-BMP signaling thereby promoting recovery of lung endothelial cell function and protection against blood vessel rarefaction, (b) to reduce pulmonary arterial pressures, and (c) to reverse adverse cardiac (and cardio-renal) remodeling.

The use of snRNA-seq analyses allowed us to characterize PAH-induced changes within the pulmonary and cardiac transcriptome. Our study demonstrates that many cell composition and transcriptional perturbations elicited by PAH are reversed by apelin analog treatment. In the lungs, PAH induced upregulation of TGF-β signaling and suppression of BMP/BMPR2 signaling in EC and FB resulting in FB expansion, activation of profibrotic FBs, and increased production of fibrotic ECM components. The major site of action for apelin-induced signaling is within the endothelium of the lung, where the apelin receptor is abundant. Therefore, we concentrated our analyses on apelin-mediated signaling in the lung to deduce the specific mechanism of apelinergic protection. Activation of apelin receptor on gCap cells resulted in an expansion of the gCap population, congruent with the restored lung vascularity seen within our apelin-treated PAH rats. gCaps have progenitor-like properties that allow them to facilitate lung reparative pathways and reform rarefied distal capillaries (19). Furthermore, apelin analog treatment normalized BMPR/SMAD1/5 signaling and induced vasodilative effects of the apelinergic pathway in the lungs. In the heart, the apelin analog conferred beneficial effects primarily in cardiomyocytes and cardiac fibroblasts by reducing cardiomyocyte hypertrophy, improving RV dilatation, minimizing interstitial fibrosis, and normalizing RV systolic function. Mild vasodilation resulting from systemic apelin analog administration is likely balanced by improved RV and LV function, increased LV stroke volume, and cardiac index, thereby minimizing the risk of hypotension as seen in the PAH rats treated with apelin analog and in patients with PAH treated with native apelin peptide (42). Our apelin peptide analog demonstrated high selectivity, high potency, and lack of hepatotoxicity, making it an excellent drug candidate for targeting the apelin receptor (43, 44). Importantly, stimulation of the apelin receptor with our apelin analog did not lead to downregulation of the apelin receptor, possibly because BMPR2 activation promotes apelin expression via the PPAR-γ/p53 transcriptional complex (7, 45, 46), thereby sustaining apelin pathway signaling.

In our study, apelin analog treatment was able to promote beneficial modulation of the TGF-β and BMP pathways in lung endothelial cells, including reduced *Tgfbr3* and increased *Acvrl1*, *Smad6*, *Smad7*, *Bmp6*, *Egfl7*, and *Id1* expression. Levels of pSMAD1/5 were also elevated in lung tissue, suggesting a shift toward BMPR2 axis activation and recovery of endothelial function. The translational potential of these findings is underscored by current therapies approved for PAH. Sotatercept, which targets the TGF-β superfamily via ActRIIA fused to the Fc domain of human IgG, has had recent success in clinical trials for PAH (17, 47). Apelin is a well-known activator of eNOS (48), leading to nitric oxide production and increased soluble cGMP. Approved therapies for PAH targeting soluble guanylate cyclase/PDE pathway, via soluble guanylate cyclase stimulator (47) or PDE5 inhibitor (49), and endothelin inhibition provide mechanistic parallels. In our study, apelin analog suppressed gene expression in both the endothelin and PDE pathways. A striking example of the therapeutic importance of targeting multiple targets is illustrated by the lack of the established therapy, endothelin receptor antagonist, to fully reverse the detrimental structural and hemodynamic changes in PAH, whereas a biased apelin receptor agonist (50) and our apelin analog provided a near-complete reversal of the PAH phenotype. The translational potential of our findings is enhanced by the reported short-term hemodynamic response to native apelin in patients with PAH (42) and the beneficial effects of the cyclic biased apelin receptor agonist in a PAH model and human volunteers (50–52).

Our study demonstrates that pulmonary and RV transcriptomic and cellular domains become aberrant in PAH and unveils cell-specific restorations and recoveries elicited by apelin analog treatment. The use of snRNA-seq analyses to characterize the cellular composition and molecular states provides an emerging benchmark in which disease-related changes and therapeutic responses can be assessed and compared (32). Elevated RVSP was alleviated by apelin therapy, partly due to apelin-induced vasodilation and reverse remodeling of the pulmonary vascular bed. Apelin-mediated stimulation of the BMPR2 axis, known to protect against arterial malformations and perivascular inflammation (2, 17, 53), elicited a robust antihyperplasia response. Restoration of BMPR2-mediated BMP signaling within the pulmonary vasculature is a promising therapeutic avenue that may also preserve RV structure and function (54). The apelinergic mechanism underlying this effect involves promoting BMPR2-mediated BMP signaling, which induces transcription of downstream effectors such as Id1. Id1 facilitates endothelial antihyperplasia (55) while inhibiting TGF-βR2–mediated TGF-β signaling, thereby fostering endothelial quiescence (33). Together, these findings support augmentation of BMPR2 pathway signaling in PAH as a promising therapeutic strategy that may also preserve RV structure and function (54).

Importantly, there is a paucity in the understanding and development of specific and targeted therapies for RV failure. Cardiomyocytes in the PAH RV demonstrated emergence of myocardial fetal reprogramming, as indicated by enhanced *Myh7* expression and reduced *Myh6* expression (32, 56); PAH reduced canonical vCM1 cells, denoting loss of functional cardiomyocytes and upregulated vCM3.1 state, involved in cellular adhesion, cytoskeletal and ECM binding. Notably, within the myocardium, apelin is predominantly expressed by cardiomyocytes, whereas the apelin receptor is enriched primarily in vasculature endothelial cells, indicating a direct paracrine signaling between these cell types. Given that BMPR2 is critical for maintaining vascular integrity and endothelial cell homeostasis (2, 51, 52), disruption of this pathway likely exacerbates the endothelial dysfunction observed in PAH. In keeping with this, rats with genetically ablated *Bmpr2* not only developed spontaneous PAH but also exhibited dysregulated K^+^ channels and impaired RV contractility (39). These findings underscore a close mechanistic link between impaired BMPR2 signaling and injury to both the lungs and RV. Our electrocardiographic findings of ST segment elevation and enlarged T wave inversion in PAH are suggestive of RV myocardial ischemia, which may be caused by a diminished transmural pressure perfusion gradient (57). Apelin analog therapy preserved the K^+^ channel levels, decreased fibrotic disruption of electrical conduction, and reversed adverse electrical remodeling. In pressure-overload induced heart failure, increased *Pdk4* and *Mpc1* expression suggest reduced glucose oxidation (58). Consistently, elevated levels of PDK4, which inhibits pyruvate dehydrogenase in the PAH-P group, indicate a shift from oxidative phosphorylation toward anaerobic glycolysis, a hallmark of PAH and characteristic of heart failure (59, 60). Apelin analog corrected this maladaptive metabolic shift.

Unexpectedly, the total number of fibroblasts in the PAH RV was reduced despite extensive ECM deposition. This paradox is likely explained by an increased proportion of activated myofibroblasts with a secretory, profibrotic phenotype driving ECM accumulation. Importantly, apelin analog treatment counteracted TGF-β–associated profibrotic mesenchymal transition in both PAH lung and RV. There was a signal toward compromised desmosomal connections between cardiomyocytes within the PAH RV, rendering the tissue permissive to immune cell infiltration, ECM deposition, and disruption of electrical junctions, thereby increasing susceptibility to arrhythmias. *Srgap1*, a downstream target of the Slit3/Robo pathway, was also upregulated, suggesting increased cardiomyocyte-fibroblast crosstalk in the setting of pressure-overload induced RV dilation and dysfunction (35). Although previous studies have demonstrated capillary rarefaction in the failing RV (61), the endothelial cell expansion observed in our study is consistent with our prior findings of augmented endothelial cell populations in both the RV and LV of human hearts with dilated cardiomyopathies (32). Moreover, 3D volumetric stereological assessment of RV vasculature in PAH showed an expansion of the RV capillary network concomitant with RV hypertrophy (62). While our study documents the benefits of apelin analog in only 1 animal model, this model best recapitulates human PAH. Our apelin analog will need to be tested in additional models of PAH (63) and in the setting of combination therapy with other therapeutic agents to better define its therapeutic applicability. Future studies should also examine the molecular changes in the RV of rat PAH models in relationship to human RV failure related to PAH (64, 65) to enhance the translatability of our findings. In addition, we need to better define the in vivo pharmacokinetics and tissue binding of our apelin analog. Given the high prevalence of PAH in women, the importance of sex-based differences in PAH in relation to the apelin pathway needs to be elucidated.

In summary, pharmacological stimulation of the apelin receptor with our analog robustly suppressed hyperproliferative, proinflammatory, mitogenic, and fibrogenic processes, effectively reversing adverse molecular perturbations seen in PAH. In parallel, apelin analog attenuated structural, fibrotic, and arrhythmogenic remodeling of the RV. Collectively, these findings position apelin analog therapy as a promising strategy to directly counteract the maladaptive pulmonary vascular and myocardial remodeling, thereby addressing disease progression and the associated morbidity and mortality in PAH.

## Methods

Supplemental Methods are available online with this article.

### Sex as a biological variable.

Our study examined male rats because of the propensity for worse prognosis in male patients with PAH, and male rats were used as the traditional model of PAH. However, given the higher prevalence of PAH in women, therapeutic effects of the apelin analog in female models of PAH require future studies.

### Study design.

The study was conducted to replicate PAH in the animal model and investigate whether apelinergic stimulation can have a therapeutic effect. The study was conducted as a controlled laboratory experiment (Figure 1A). The endpoint (8 weeks: 3 weeks of hypoxia followed by 5 weeks of normoxia) was selected to ensure the development of the advanced phenotype and allow sufficient treatment time, which was introduced after 5 weeks. We used the traditional and established model of PAH based on male Sprague-Dawley rats (Charles River) aged 9–10 weeks (weighing 250–300 g) with implanted jugular vein catheters (15, 16). PAH was induced using SU/Hx protocol (Figure 1A). Rats (*n* = 40) were injected with VEGF receptor inhibitor, SU5416 (Sigma Aldrich, S8442, 20 mg/kg, dissolved in 0.9% carboxymethylcellulose, 0.9% saline, 0.4% polysorbate 80, and 0.6% benzyl alcohol) s.c. before being placed in hypoxia chambers of 10% oxygen for 3 weeks. Rats were reexposed to normoxia for 5 weeks, with twice-daily i.v. injections occurring within the final 3 weeks of normoxia. Rats were randomized between placebo and treatment group before starting of injections of either placebo (saline) or apelin analog (0.25 μmol/kg in saline solution). Rats that did not reach the endpoint were excluded from the study (2 rats died in hypoxia chambers, and 2 rats had catheter blockage). Healthy control animals (*n* = 16) were housed in normal room air for 8 weeks in the same hypoxia chambers before the experimental endpoint. Safety of apelin analog administration was confirmed in a separate experiment with rat receiving either placebo (saline; *n* = 8) or apelin analog (0.25 μmol/kg in saline solution; *n* = 8) at the same time points as in the PAH study (Figure 1A).

Sample sizes and experimental designs were determined on the basis of previously published data from our laboratories. Blinding was used only during scoring and analysis of experimental data. For snRNA-seq, 4 random tissue samples were selected from each group (CTRL, PAH-P, and PAH-A).

### Statistics.

To assess differences between groups, Graphpad Prism 9 and R were used to generate plots and conduct 1-way ANOVA with Tukey’s post hoc analysis for multiple comparisons. A *P* value of less than 0.05 was considered significant.

### Study approval.

Animal procedures were performed in accordance with the Canadian Council on Animal Care guidelines, and the animal protocol was reviewed and approved by the IACUC at the University of Alberta (AUP00003022).

### Data availability.

RNA transcriptomics data have been deposited NCBI’s Gene Expression Omnibus (GSE330160). All other data are available in the main text, the supplemental materials, or Supporting Data Values file. The biosensors used for generating the data in Supplemental Figure 1B are protected by a patent but are available from MB for noncommercial research without restrictions under a regular academic Material Transfer Agreement with the Université de Montréal.

## Author contributions

JV, PZ, and GYO conceptualized the study and animal study design. JGS, CES, and YK conceptualized the study and transcriptomic study design. GYO, JCV, JGS, and MB were responsible for funding acquisition. GYO, JGS, CES, and JCV administered the project. GYO, JGS, CES, JCV, and MB supervised the studies. JV, PZ, AV, and MA conducted in vivo experiments and tissue collection. JV, HC, TUJ, FW, AE, AW, CF, and CA performed in vitro and histological studies. KJB, DMD, and JG were responsible for snRNA-seq. KJB, DMD, and PZ performed bioinformatic analysis. JV, PZ, KJB, and DMD were responsible for visualization. JV, PZ, and GYO wrote the original draft. All authors, including MCC, reviewed the manuscript and contributed to the editing. JV, PZ, and KJB contributed equally to the manuscript as co–first authors. The sequence of the co–first authors has been set according to relative contributions of the co–first authors. GYO, JGS, and CES contributed equally to the study.

## Conflict of interest

GYO and JCV are co-owners of apelin analog patents (US10723776B1; EP3649146A1). MB is the chair of the scientific advisory board of Domain Therapeutics, to which some of the BRET-based biosensors used are licensed for commercial use.

## Funding support

This work is the result of NIH funding, in whole or in part, and is subject to the NIH Public Access Policy. Through acceptance of this federal funding, the NIH has been given a right to make the work publicly available in PubMed Central.

- Canadian Institute of Health Research grants PJT-425751, PJT-462950 (GYO).
- Canadian Institute of Health Research grant PJT-166151 (JCV).
- National Institutes of Health grants R01 HL080494, 5U01HL098147 (JGS).
- Canadian Institute of Health Research grant PJT-183758 (MB).

## Supplementary Material

Supplemental data

Unedited blot and gel images

Supplemental table 1

Supplemental table 2

Supplemental table 3

Supplemental table 4

Supplemental table 5

Supplemental table 6

Supplemental table 7

Supplemental table 8

Supplemental table 9

Supplemental table 10

Supplemental table 11

Supplemental table 12

Supplemental table 13

Supplemental table 14

Supplemental table 15

Supplemental table 16

Supplemental video 1

Supplemental video 2

Supporting data values

## Version 1 06/18/2026

In-Press Preview

## Version 2 08/10/2026

Electronic publication

## Version 3 08/11/2026

Metadata fix
