# Supplementary material for: Apelin analog treatment reverses severe pulmonary arterial hypertension and right ventricular heart failure
Source: JCI Insight. 2026 Jun 18;11(15):e200684. doi: 10.1172/jci.insight.200684 (PMC13464015; doi:10.1172/jci.insight.200684)
Supplement: Supplementary file 1 [file jciinsight-11-200684-s348.pdf]

Supplemental Material

Title: Apelin analog treatment reverses severe pulmonary arterial hypertension and right ventricular heart failure

Authors: Jennie Vu[1,†], Pavel Zhabyeyev[2,†], Kemar J. Brown[3,4,†], Joshua Gorham[3], Daniel M. DeLaughter[3], Huachen Chen[2], Thilina U. Jayawardena[2], Ander Vergara[2], Maria Alexiou[5], Anjalee Wijewardane[6], Conrad Fischer[6], Charlotte Avet[7], Abby Ewasiuk[2], Faqi Wang[2], Mark C. Chappell[8], Yuri Kim[3,9], Michel Bouvier[7], John C. Vederas[6], Christine E. Seidman[3,9,#], Jonathan G. Seidman[3,#], and Gavin Y. Oudit[1,2,10*,#]

Affiliations:
[1]Department of Physiology, University of Alberta; Edmonton, AB, Canada.
[2]Department of Medicine, University of Alberta; Edmonton, AB, Canada.
[3]Department of Genetics, Harvard Medical School; Boston, MA, USA.
[4]Division of Cardiovascular Medicine, Massachusetts General Hospital; Boston, MA, USA.
[5]Department of Dentistry & Dental Hygiene, University of Alberta; Edmonton, AB, Canada.
[6]Department of Chemistry, University of Alberta, Edmonton, AB, Canada.
[7]Institute for Research in Immunology and Cancer, Université de Montréal, Montréal, QC, Canada.
[8]Department of Surgery/Hypertension and Vascular Research, Wake Forest School of Medicine; Winston-Salem, NC, USA.
[9]Division of Cardiovascular Medicine, Brigham and Women's Hospital; Boston, MA, USA.
[10]Mazankowski Alberta Heart Institute, Alberta Health Services; Edmonton, AB, Canada.

*Corresponding author. †These authors contributed equally; # equal contributions

Address for correspondence: Gavin Y. Oudit
116 St and 85 Ave, HMRC-407
Edmonton, Alberta T6G 2R3, Canada
University of Alberta
780 407 8569
gavin.oudit@ualberta.ca

MATERIALS AND METHODS

Single-nucleus RNA sequencing

Nuclei were isolated from flash frozen tissue as described (1). Nuclei were examined automatically or manually for viability after staining with Trypan Blue using a Countess II machine (Invitrogen). Viable nuclei suspensions were loaded into chips and onto a Chromium Controller (10X Genomics) at a density targeting 5,000 to 10,000 cells for recovery per sample following the manufacturers protocol for single nuclei 3′ libraries (10x Genomics, version 3). Successful libraries were then sequenced on an Illumina NovaSeq machine to a depth of approximately 50,000 reads per nuclei. Bcl files generated by the sequencer were processed into fastq files and aligned to the *Rattus norvegicus* genome (mRatBN7.2) using the CellRanger software suite (10x Genomics, version 3.0.2). Aligned reads for each sample were run through CellBender software to reduce ambient background reads and other technical artifacts (https://github.com/broadinstitute/CellBender). The annotation and analysis for the RV were based on our studies (2-4). For the lungs, average expression was calculated for each sample (12 total) and then genes with the absolute log2 fold change of less than 0.25 were removed. Only genes present in more than 75% within each group were kept to remove outliers and sample specific effects. Lung marker genes were used for annotating (5-7).

Downstream quality control filtering and clustering analysis were done using the Seurat software package (version 3.1.1). Each sample was subjected to nuclei doublet analysis using Solo (https://github.com/calico/solo). Single nuclei were filtered for having the number of genes per nuclei greater than 200, read counts per nuclei less than 50,000, for mitochondrial reads to be less than or equal to 5% of total reads, and for solo scores less than 0.5. To control for batch effects between samples the Seurat object was processed with Harmony (https://github.com/immunogenomics/harmony). The effect of mitochondrial reads was regressed out, and the data normalized and scaled. Principal Components (PCs) were calculated and the top 50 PCs were suitable for neighbor graph construction. Cells were clustered using the network-based Louvain algorithm. Uniform Manifold Approximation and Projection (UMAP) plots were generated to visualize the data. A gene was considered to be differentially expressed if it has a $\log_2$-transformed fold change > 0.5 and P adjusted value (P_{adj}) < 0.05 (2, 3).

Cell culture

For the proliferation assay, healthy Human Pulmonary Artery Endothelial Cells (HPAEC) (ATCC; PCS-100-022) were cultured in Vascular Cell Basal Medium (ATCC PCS-100-030) supplemented with Endothelial Cell Growth Kit-VEGF (ATCC; PCS-100-041) at 37 °C in a humidified atmosphere of 5% CO_2. Cells were grown to 70% confluency.

To induce hyper proliferating state, the cells were subjected to 150 ng/mL FGF-2 (R&D Systems, #233-FB) and 50 ng/mL VEGF to induce proliferation. Apelin was tested in each well of cells at doses of 0, 0.25, 1, and 5 μM to observe anti-hyperproliferation effects. A commercial Click-It Plus EdU Alexa Fluor 488 kit (Thermofisher, C10632) was used and cells were collected and analyzed with BL1 detectors during flow cytometrical analyses (8, 9).

Cells were assigned to the following experimental groups: (i) control, (ii) SU5416 + hypoxia, and (iii) SU5416 + hypoxia + apelin analog. SU5416 and apelin analog were used at concentrations established in preliminary experiments. Hypoxic exposure was performed at 1% O_2 (5% CO_2, balance N_2).

Viability in HPAEC cells was assessed using. MTT (3-(4,5-dimethylthiazol-2-yl)-2,5-diphenyltetrazolium bromide) (10). HPAECs were seeded at a density of 5,000 cells/well in a 96-

well plate and incubated overnight. Cells were then treated with vehicle (DMSO), SU5416 (1 µM), Apelin analog (0.1, 0.5, or 1 µM), or their combinations under normoxic or hypoxic (1% O_2) conditions. After 24 hours, the medium was replaced with 25 µL Opti-MEM and 25 µL of MTT solution (5 mg/mL). Plates were incubated at 37 °C for 3 hours. The resulting formazan crystals were solubilized with 50 µL DMSO, and absorbance was measured at 570–590 nm. Cell viability was calculated as a percentage relative to vehicle-treated controls.

Nitric oxide (NO) release was measured using the DAF-FM DA fluorescent probe, which detects intracellular NO levels (11). Cells were incubated with 5 µM DAF-FM DA (Invitrogen) diluted in phenol-red-free complete endothelial growth medium for 10 minutes at 37 °C in the dark. After incubation, excess dye was removed by washing with fresh medium. Fluorescence was measured using a microplate reader (excitation 495 nm, emission 515 nm), and NO production was expressed as relative fluorescence units (RFU), normalized to vehicle-treated controls.

Cyclic GMP (cGMP) levels were measured using the Direct cGMP ELISA kit (Enzo Life Sciences, ADI-900-014) according to the manufacturer's instructions. Cells were washed with PBS and lysed directly in the culture plates using ice-cold 0.1 M hydrochloric acid (HCl), added at a volume sufficient to cover the bottom of each well (~250–300 µL/well). Plates were incubated for 10 minutes at room temperature to ensure complete cell lysis. Lysates were collected and centrifuged at ≥600 ×g for 10 minutes to pellet cellular debris. The supernatant was collected and stored at −80 °C until analysis. Immediately prior to the assay, samples and standards were acetylated by adding 10 µL of freshly prepared Acetylation Reagent (1 part acetic anhydride to 2 parts triethylamine) per 200 µL of sample or standard, followed by brief vortexing. Acetylated samples and standards were loaded in duplicate onto a microtiter plate pre-coated with goat anti-rabbit IgG. cGMP conjugate and anti-cGMP antibody were added, and the plate was incubated for 2 hours at room temperature on a microplate shaker (500 rpm). After washing, pNPP substrate was added, and the plate was incubated for 1 hour at room temperature (21 ºC) in the dark. Stop solution was added, and absorbance was measured at 405 nm using a microplate reader. A standard curve was generated from serial dilutions of the cGMP standard (0.08–50 pmol/mL), and sample concentrations were interpolated using a 4-parameter logistic fit. Final values were normalized to total protein concentration and expressed as pmol cGMP per mg of total protein.

Cell Migration Assays

For transwell migration assay (12), HPAEC migration was assessed using a transwell chamber system with Falcon® cell culture inserts (12-well format, 3.0-µm pore size, transparent PET membrane). The lower chambers were filled with 1.5-2.0 mL of chemoattractant medium containing complete growth medium. HPAECs were harvested, counted, and resuspended in serum-reduced medium, and 5×10^4–2×10^5 cells were seeded into the upper chamber in 0.4-0.8 mL. Cells were treated according to group assignment at the time of seeding. Control cells were maintained under normoxic conditions, whereas SU5416-treated groups were placed under hypoxia immediately after seeding. For baseline measurements (0 h), a dedicated set of inserts was fixed immediately after cell seeding. Migration assays were otherwise allowed to proceed for 8 h. At the designated endpoints, non-migrated cells on the upper surface of the membrane were removed using a cotton swab. Migrated cells on the lower surface were fixed with 4% paraformaldehyde for 10-15 min at room temperature, washed with PBS, and stained with 0.1% crystal violet solution. Excess stain was removed by extensive washing with distilled water. Membranes were air-dried and imaged using bright-field microscopy. Migration was quantified by counting stained cells in at least five random fields per insert or by measuring stained area using ImageJ software. Data are expressed as migrated cells per field or as fold change relative to control.

For the wound-healing assay (13), HPAECs were seeded into 12-well plates and grown to full confluence. Cells were serum-reduced overnight prior to wounding. A uniform linear scratch was generated across the monolayer using a sterile 200-μL pipette tip. Detached cells were removed by washing twice with PBS, and serum-reduced medium was added. Cells were treated immediately according to experimental group assignment. Images of the wound area were captured at 0 h using phase-contrast microscopy, and plates were then incubated under normoxic or hypoxic conditions as appropriate. After 8 h, images were acquired from the same wound locations. Wound closure was quantified using ImageJ software by measuring wound area at 0 h and 8 h. Migration was expressed as percentage wound closure calculated as:

$$\text{Wound closure (\%)} = \frac{(\text{Area}_{0h} - \text{Area}_{8h})}{\text{Area}_{0h}} \times 100$$

All experiments were performed with three technical replicates and repeated independently at least three times.

Flow cytometry

Apoptosis and cell death were assessed by flow cytometry (14) using the Dead Cell Apoptosis Kit with Annexin V-FITC and Propidium Iodide (PI) (Thermo Fisher Scientific, V13242), according to the manufacturer's instructions with minor adaptations for adherent endothelial cells. HPAECs were assigned to the following experimental groups: (i) control, (ii) SU5416 / hypoxia, and (iii) SU5416 / hypoxia + apelin analog. Cells were analyzed at 0 h and 8 h following treatment. Both floating and adherent cells were collected to ensure accurate quantification of apoptotic and dead populations. Culture supernatants were retained, and adherent cells were gently detached using brief enzymatic dissociation. Detached cells were combined with their corresponding supernatants, centrifuged, and washed once with cold phosphate-buffered saline (PBS). Cell pellets were resuspended in 1X annexin-binding buffer prepared by diluting the supplied 5X buffer with deionized water. Cells were adjusted to a concentration of approximately 1×10^6 cells/mL, and 100 μL of cell suspension was transferred to individual flow cytometry tubes for staining with V-FITC and propidium iodide (PI). For each sample, 5 μL Annexin V–FITC and 1 μL PI were added to the 100 μL cell suspension. Samples were gently mixed and incubated for 15 min at room temperature (21ºC) in the dark. Following incubation, 400 μL of 1X annexin-binding buffer was added to each tube, and samples were kept on ice and analyzed by flow cytometry within 1 hour. Unstained cells, Annexin V-FITC-only stained cells, and PI-only stained cells were prepared as controls for setting voltages, compensation, and gating. Compensation was performed using single-stained controls prior to data acquisition.

Data were acquired on a BD LSR Fortessa X-20 flow cytometer. The FITC fluorescence was detected using the 488-nm laser with a 530/30-nm emission filter, and the PI fluorescence was detected using 580/30-nm emission filter. At least 10,000 single-cell events were collected per sample. Data were analyzed using standard gating strategies. Briefly, debris was excluded based on forward- and side-scatter properties, and doublets were excluded using forward scatter area versus height. Apoptotic populations were identified using bivariate plots of Annexin V-FITC versus PI fluorescence. Cells were classified as viable (Annexin V^-/PI^-), early apoptotic (Annexin V^+/PI^-), late apoptotic or secondary necrotic (Annexin V^+/PI^+), or necrotic (Annexin V^-/PI^+). Results were expressed as the percentage of total cells within each population.

Tissue collection and histology

One lung was ligated and excised to be snapped frozen in liquid nitrogen, the remaining lung was inflated with 10% formalin via cannulation of the trachea, the lung was removed *en bloc*.

Alternatively, the remaining untied lung was inflated with 50% optimal cutting medium and 50% phosphate buffered saline (PBS) and a small portion was cut and placed in a cassette then snapped frozen. The heart was excised, doused in 1M potassium chloride (KCl) to induce diastole, and the left and right ventricles were separated and weighed; alternatively, the whole heart was submerged in KCl, briefly in liquid nitrogen, and then a 0.5 cm section between base and apex was placed in a cassette with optimal cutting medium (OCT) then snapped frozen, or placed in 10% formalin for 48 hr fixation. The liver was excised, weighed, small part was placed in 10% formalin for 48 hr fixation, and the rest of the liver was frozen in liquid nitrogen. Using paraffin embedded sections that were cut 5 μm thick, Hematoxylin and eosin (H&E) and Masson's trichrome staining was carried out in accordance with standard protocols. PSR staining also utilized paraffin sectioned samples and followed a protocol entailing deparaffinization with xylene, subsequent rehydration with diluted ethanol concentrations, submersion of slides into 0.2% phosphomolybdic acid, and then immersing slides into picric acid with 0.1% Sirius red solution (9:1 v/v respectively). Slides were then added into 0.5% acetic acid and washed with ethanol followed by xylene before mounting. Immunofluorescence staining was conducted using OCT preserved tissues. In brief, tissues were preserved with 4% paraformaldehyde solution, and permeabilized with 0.1% triton X-100. Primary antibodies were administered overnight in 2% BSA at 4 °C, and secondary antibodies were used at a 1:1000 dilution with 2% BSA for 1 hour. All washes in between steps were completed with PBS. Slides were mounted with gold prolong antifade reagent with DAPI. Images were acquired by a confocal laser-scanning fluorescence microscope and processed with Leica Application Suite X software (Leica Microsystem). Antibodies utilized for immunocytochemistry were goat anti-rat Ki67 (Santa Cruz M-19, 1:100 dilution), rabbit anti-rat Kcnd2 (Alomone labs, Jerusalem, #APC-023, 1:100 dilution), mouse anti-rat cardiac troponin T (Invitrogen, MA5-12960, 1:100 dilution), mouse anti-rat α-actin (Santa Cruz, sc-32251, 1:50 dilution), mouse anti-rat periostin (Santa Cruz, sc-398631, 1:50 dilution), rabbit anti-rat Pdgfr-α (Abcam, ab203491, 1:50 dilution), rabbit anti-rat CD31 (Abcam, AB222783, 1:50 dilution), rabbit anti-CD163 (Thermo Fisher, BS-2527, 1:50 dilution), and rabbit anti-ERG (Abcam, ab92513, 1:50 dilution). For secondary antibodies, the following fluorescent conjugates from Thermo Fisher (Waltham, Massachusetts, USA) were used: anti-goat Alexa 568 (A-11057), anti-mouse Alexa 647 (A-21235), anti-rabbit Alexa 568 (A-11011), anti-rabbit Alexa 647 (A-31573), and anti-mouse Alexa 568 (A-11004). Wheat germ agglutinin (WGA), conjugated to Alexa 488 (W32466, Thermo Fisher), was used to stain cellular membranes. Images were acquired using a Leica Stellaris 8 confocal laser-scanning fluorescence microscope (Leica Microsystems, Wetzlar, Hesse, Germany) and processed with Leica Application Suite X software.

In vitro plasma stability assay by High Performance Liquid Chromatography (HPLC)

20 μL of human or rat plasma was pipetted into 1.7 mL Eppendorf tubes and incubated at 37 °C for 10 mins. 5 μL of apelin analog (400 μM) was added, mixed and then incubated at 37 °C for various time intervals. The solutions were then quenched with the addition of 20 μL of 10% aqueous TFA, followed by the addition of 5 μL (1 mM) of an internal standard; Dansyl-YVG-OH (Bachem) for native apelin-17 and dansyl amide for apelin analog. The experiments were diluted with another 20 μL of 0.5% aqueous TFA in 5% ACN and then centrifuged at 10,200 g for 10 min. The supernatant was then loaded onto a pre-equilibrated C18 (G-Biosciences) or biphenyl spin column, and was centrifuged at 1500 g for 1 minute. The flow-through was reapplied to the resin bed and the centrifugation was repeated to ensure binding. The resin was then washed with 2 x 200 μL with 0.5% aqueous TFA in 5% ACN, with the filtrate being discarded after each wash. The desired peptides were then eluted with 2 x 20 μL of 40% ACN in 0.1% TFA with centrifugation at 1500 g for 1 minute. The eluate was stored at −20 °C until loading on RP-HPLC

using the method described previously. The ratio of the apelin peptide to the internal standard was calculated by comparing areas under the peaks on the HPLC chromatogram. The 0 min incubation internal standard/analogue area ratio was used to compare the internal standard/analogue area ratio for the timed experiments and determine the remaining % analogue. Experiments were performed in triplicates. Analytical scale HPLC was performed on an Agilent 120 Infinity II chromatograph. HPLC solvents were filtered through a Millipore filtration system under vacuum prior to use. The degradation of apelin was monitored using diphenyl RP-HPLC column with aqueous 0.1% TFA (solvent A) and 0.1% TFA in acetonitrile (solvent B) as eluents. The analytical purification method used was 0–5 min, 10% B; 9-11 min, ramp 10-60% B, 11-15 min, 100% B; 15-16 min, ramp 100-10% B; and 16-21 min, 10% B.

Plasma collection and blood work

Ten minutes prior blood collection, rats were given heparin (IV, 1000 IU/kg). Blood (2-3 ml) was collected from carotid artery after euthanasia and spun down at 4000 g for 10 min. Plasma was aspirated and frozen in liquid nitrogen. Blood work for bilirubin, SDH, ALT, AST, and ALP was performed by IDEXX Laboratories Canada using Beckman AU chemistry analyzer.

Bioluminescence resonance energy transfer assay in HEK293 cells

HEK293 clonal cell line (HEK293SL cells), hereafter referred as HEK293 cells, were a gift from S. Laporte (McGill University, Montreal, Quebec, Canada) and previously described (15). Cells were cultured in DMEM medium (Wisent; St-Jean-Baptiste, QC, Canada) supplemented with 10% newborn calf serum iron fortified (NCS; Wisent) and 1% of antibiotics (100 U/mL penicillin and 100 µg/mL streptomycin). Cells were passaged weekly and incubated at 37°C in a humidified atmosphere with 5% CO_2 and checked for mycoplasma contamination. Forty-eight hours before the experiments, 1 µg of total DNA (adjusted with salmon sperm DNA; Invitrogen) was used to transfect 3.5×10^5 cells/mL using linear polyethylenimine (PEI, 1 mg/mL; Polysciences) diluted in NaCl (150 mM pH 7.0) as a transfecting agent (3:1 PEI/DNA ratio). Cells were immediately seeded (3.5×10^4 cells/well) in 96-well white microplates (Greiner Bio one). Cells were maintained in culture for the next 48 h and BRET experiments carried out.

Enhanced bystander BRET (ebBRET) was used to monitor the activation of Gαi1, Gαi2, Gαi3, GαoB, Gα12, Gα13, Gαq, Gα11, Gα15 and Gαs proteins, as well as β-arrestin 1 and 2 recruitment to the plasma membrane. Gα proteins activation was followed using APJ receptor and the plasma membrane marker rGFP-CAAX with either the selective-Gi/o effector Rap1GAP-RlucII along with the human Gαi1, Gαi2, Gαi3 or GαoB subunits, or the selective-Gq/11 effector p63-RlucII along with the human Gαq, Gα11 or Gα15 subunits, or the selective-G12/13 effector PDZ-RhoGEF-RlucII along with the human Gα12 or Gα13 subunits (16). Gαs protein engagement was measured between rGFP-CAAX and human Gαs-RlucII in presence of human Gβ1, Gγ1 and APJ receptor (16). β-arrestin recruitment to the plasma membrane was determined using β-arrestin1-RlucII or β-arrestin2-RlucII with rGFP-CAAX (15) and APJ receptor. On the day of the experiment, cells were washed with phosphate-buffered saline (PBS) and incubated in Tyrode HEPES buffer (137 NaCl, 0.9 KCl, 1 $MgCl_2$, 11.9 $NaHCO_3$, 3.6 NaH_2PO_4, 25 HEPES, 5.5 D-Glucose, and 1 $CaCl_2$ in mmol/L; pH 7.4) for 1 h at 37°C. Cells were then treated with increasing concentrations of compounds for 10 min at 37°C. The luciferase substrate Prolume purple (1 µmol/L, NanoLight Technologies) was added during the last 6 min before the reading. BRET measurements were performed on a Tecan Spark multimode microplate reader (Männedorf, Switzerland) using BRET2 setting: wavelength between 360 and 440 nm (donor) and, 505 and 575 nm (acceptor) for detecting the RlucII (donor) and rGFP (acceptor) light emissions, respectively. BRET signal ($BRET^2$) was determined by calculating the ratio of the light intensity

emitted by the acceptor over the light intensity emitted by the donor and normalized in percentage of the maximal response elicited by the reference compound apelin-17. The ligand-promoted BRET signal (ΔBRET) was calculated as the difference in BRET recorded from cells treated with compound and cells treated with vehicle before normalization in percentage of the maximal response elicited by the reference compound apelin-17. The data were analyzed in GraphPad Prism 10.4.1 using "log(agonist) vs. response – Variable slope (four parameters)". 3-6 independent experiments were performed.

Immunofluorescence staining of human pulmonary artery endothelial cells (HPAECs)

Immunofluorescence staining was performed to visualize nuclei, adherens junctions, and the actin cytoskeleton in HPAEC cells using DAPI, VE-Cadherin, and Rhodamine Phalloidin, respectively (17).

Cells were seeded in 4-well chamber slides and treated under indicated conditions. After treatment, cells were gently washed with phosphate-buffered saline (PBS) and fixed with 4% paraformaldehyde (PFA) for 15 minutes at room temperature. Fixed cells were then permeabilized with 0.1% Triton X-100 in PBS for 10 minutes and blocked with 1% bovine serum albumin (BSA) in PBS for 30 minutes at room temperature to reduce nonspecific binding.

For VE-Cadherin staining, cells were incubated with a primary anti-VE-Cadherin antibody rabbit anti-VE-Cadherin, (Cell Signaling, #2500S) diluted in blocking buffer (1:100) for 1 hour at room temperature. After washing, cells were incubated with an Alexa Fluor-conjugated secondary antibody (donkey anti-rabbit Alexa Fluor 488, Invitrogen; A-21206, 1:500 dilution) for 1 hour in the dark at room temperature.

Actin filaments were stained by incubating cells with Rhodamine Phalloidin (Invitrogen; R415; 1:400 dilution) for 20 minutes at room temperature in the dark. After the final washes, the chamber walls were removed, and the slides were mounted using ProLong™ Gold Antifade Mountant with DAPI (Invitrogen, P36935). To visualize cell migration protrusions, the actin cytoskeleton (Rhodamine Phalloidin), focal adhesion kinase activation (phospho-FAK Tyr397; #83933-1-RR; Proteintech), and focal adhesion plaques (Vinculin; sc-73614; Santa Cruz Biotechnology) were used. Coverslips were applied, and the slides were allowed to cure in the dark at room temperature before imaging on a fluorescence microscope.

Western blotting

For tissue samples, lysates were prepared by homogenizing tissue in a Tissue Lyser with CellLytic buffer with added phosphatase and protease inhibitors. Immunoblotting was performed using 10% SDS gels, samples were run at 90V, and transfer was done at 4 °C for 3 hours at 0.24 mAmps, then membranes were blocked with 5% milk in tris-buffered-saline with 0.1% tween 20. At a concentration of 1:1000 in 5% BSA overnight at 4 °C, the antibodies used were rabbit anti-rat pSMAD1/5 (Cell signaling, #9516), goat anti-rat PDK4 (Santa Cruz C-16), apelin (Santa Cruz Biotechnology, 2A1-2D5: sc-293441), and apelin receptor (Invitrogen, #PA5-114830) diluted 1:1000 in TBST with 3% BSA. After washing, membranes were incubated with HRP-conjugated anti-rabbit/anti-mouse/anti-rat/anti-goat IgG secondary antibody (Cell Signaling Technology, #7074, #7076, #7077, Invitrogen #A15999; 1:3000) for 1 hour at room temperature. Total protein was acquired using Pierce reversible protein stain kit for PVDF membranes (Thermo Scientific™ 24585).

For cells in the culture, cells were treated under indicated conditions and harvested 10 minutes post-treatment for protein extraction. Cells were lysed in ice-cold RIPA buffer (Thermo Fisher Scientific) supplemented with protease and phosphatase inhibitor cocktails (cOmplete™ ULTRA

Tablets, Roche), incubated on ice for 30 minutes, and centrifuged at 12,000 × g for 15 minutes at 4 °C. The supernatant was collected, and protein concentration was quantified using the BCA assay (Pierce™ BCA Protein Assay Kit, Thermo Fisher Scientific). Equal amounts of protein (15 μg) were resolved by SDS-PAGE on an 8% polyacrylamide gel and transferred onto PVDF membranes (Millipore). Membranes were blocked with 3% BSA in Tris-buffered saline with 0.1% Tween-20 (TBST) for 1 hour at room temperature, then incubated overnight at 4 °C with the following primary antibodies: phospho-eNOS (Ser1177) (Cell Signaling Technology, #9570), total eNOS (Cell Signaling Technology, #5880), apelin (Santa Cruz Biotechnology, 2A1-2D5: sc-293441), apelin receptor (Invitrogen, #PA5-114830), phospho-Akt (Ser473) (Cell Signaling Technology, #4060), total Akt (Cell Signaling Technology, #9272), phospho-ERK (Cell Signaling Technology, #4370), total ERK (Cell Signaling Technology, #4696) diluted 1:1000 in TBST with 3% BSA. Apelin antibodies (Abcam ab59469 and Pheonix peptide Apelin-36) were used to check for apelin protein levels but were unsuccessful to produce any bands at the end of the experiment. After washing, membranes were incubated with HRP-conjugated anti-rabbit/anti-mouse IgG secondary antibody (Cell Signaling Technology, #7074, #7076; 1:3000) for 1 hour at room temperature. Protein bands were visualized using enhanced chemiluminescence (ECL; BioRad, #1705062) and imaged with a ChemiDoc system (ImageQuant™ LAS 4000 biomolecular imager). Densitometric analysis was performed using ImageJ, and phospho-protein levels were normalized to total protein (18).

Hydroxyproline assay

Wet tissue mass was weighed and then lyophilized for 2 hours. Dry tissues were homogenized (in a Tissue Lyser at 25 Hz, 3 mins with metal bead) with 6M HCl. Tissues were heated at 180 °C in a heating block for 18 hours. Tubes were then cooled to room temperature and neutralized with 6M NaOH, and centrifuged at 15000Xg (15 mins). 400 μL was aliquoted from each tube, and 1.4% chloramine T solution was added to each aliquot and hydroxyproline standards. Samples were incubated at room temperature for 20 minutes. 200 μL of Ehrlich reagent was added to each sample and vortexed. Samples were incubated at 65 °C for 20 minutes. 200 μL of each sample was loaded into a plate and the absorbance was read at 558 nm.

Urine albumin-to-creatinine ratio measurement

Urine was obtained at the end of the experiment through bladder puncture. Urine albumin and creatinine levels were measured using commercial assays (Nephrat II, Ethos Biosciences, USA, and The Creatinine Companion, Ethos Biosciences, USA) following the manufacturer's instructions. Albumin-to-creatinine ratio is expressed in μg/mg.

Glomerular morphology evaluation

Mesangial matrix expansion was assessed in 20 representative cortical glomeruli captured from Periodic Acid Schiff (PAS) stained renal sections. Briefly, paraffin blocks were cut at 4μm, deparaffinized in xylene and rehydrated through graded alcohols. Kidney sections were stained following PAS staining protocol and using Schiff's reagent. Counterstain was performed with Haematoxylin Gill Nº3 solution. Glomerular area and tuft-adjusted mesangial matrix area were measured in each 400x magnification image. All histology images in the study were analyzed by an independent blinded observer using ImageJ (v1.53a, National Institutes of Health, USA).

EchoMRI and echocardiography

Rats were lured into a plastic tube, slightly compressed, and placed into the EchoMRI machine (Whole Body Composition Analyzer EF-2014, Echo Medical Systems, Houston, Texas) for

measurement of body composition. ECG-gated transthoracic echocardiography using Vevo 3100 System and MX250 transducer probe. Rats were secured in supine position and anesthesia was induced with 3% gaseous isoflurane and maintained at 1.5-2.0% throughout the procedure. M-mode and B-mode videos were captured in parasternal longitudinal axis and short axis. Left atrial (LA) dimension was captured in the same plane as the aortic valve. The probe was aimed below the rib cage to achieve a subcostal angle for the apical 4-chamber view. Pulmonary arterial flow was found by viewing the heart in short axis and moving the probe close to the base of the heart where colored pulse wave Doppler flow identified location, direction, and intensity of flow, and allowed for measurement of the pulmonary artery flow profile. The pulmonary artery was visualized at the base of the heart in the short axis view with the aortic valve in sight. RVFWT was measured during M-Mode parasternal short and long axis at end-diastole and averaged. TAPSE and tricuspid valve flow were obtained using the 4-chamber view; TAPSE was the result of tracking the movement of lateral tricuspid annulus with M-Mode. For better visualization of the right ventricle, the probe was positioned from the right side of the rat. Analysis was performed using Vevo LAB 5.7.0. Ejection fraction was measured using LV parasternal long axis analyses.

Electrocardiography

Rats were under isoflurane anesthesia (1.5-2%) on a heated pad (body temperature maintained at 37 °C, measured by the rectal probe). ECG leads were placed in Lead I configuration. Signal was digitized using the acquisition interface ACQ-7700 (Data Science 2 International, USA) with P3 Plus software (ver. 5.0, Data Science International, USA). For each rat, 5 seconds of ECG recording were analyzed using Ponemah software.

Transcutaneous glomerular filtration rate measurement

Glomerular filtration rate (GFR) was assessed at the end of the treatment by transcutaneous measurement of fluorescein isothiocyanate bounded sinistrin (FITC-sinistrin) clearance. Briefly, under isoflurane anesthesia, a side of the rat's back was shaved. In that portion of the skin, a fluorescent signal measuring device (Transdermal Mini GFR monitor, MediBeacon, Germany) was attached with an adhesive patch and silk tape. After recording for 5 minutes the background signal of the skin, an intravenous bolus of a fluorescent agent (FITC-sinistrin) was administered at a dose of 3mg/100g body weight. Then isoflurane anesthesia was stopped and the signal decay of FITC-sinistrin was recorded in the conscious rats for one hour and thirty minutes before removing the device. Data was analyzed with a specific software (MPD Studio Version RC15, MediBeacon, Germany) using a 3-compartment model to obtain FITC-sinistrin half-life, which was converted to µL/min using a previously validated formula (19).

Right heart catheterization

Blood glucose was measured prior to anesthetic induction using a Contour Next glucometer. Rat anesthesia was induced with 4% isoflurane and maintained with 2.5% isoflurane. With the rat positioned in a supine position, an incision was made 2.5 cm to the right of the midline near the neck. The jugular vein was isolated, and the distal end was tied off and secured. The pre-implanted plastic cannula within the vein was removed, and the Transonic pressure volume transducer catheter (1.9F rat catheter, SciSense) was inserted and advanced towards the right atrium, and then maneuvered to enter the right ventricle. A suture was tied around the jugular vein to minimize blood loss, and another was tied to secure the inserted catheter. Online calculations and live analysis of pressure waveforms were accomplished using the Transonic ADV550 system. Offline calculations and analysis of hemodynamic data were performed with LabScribe 4.0.

Lung barium-contrast perfusion & Micro-CT angiography

In brief, rat anesthesia was maintained at 3% gaseous isoflurane, the thoracic cavity was exposed and RVOT was sliced. Polyethylene-90 tubing entered the RVOT and was advanced to cannulate the pulmonary artery. Lungs were first flushed with 60 mL of heparinized 0.9% saline. Barium gelatin suspension consisted of 17mL ddH_2O, 2.5 g gelatin, and 35 mL barium sulfate solution (Polibar+, E-2-2M Canada Inc, QC) dissolved over low heat and constant stirring. 10 mL of barium gelatin suspension was perfused through the lungs at a pressure approximating the right ventricular systolic pressure. 3D volumetric rendering of the lung was performed by 3D splicer computing platform, which allowed for quantification of the vasculature volume and total lung volume while maintaining a constant threshold. Following perfusion with barium the lungs were fixed overnight in 4% PFA. They were imaged using a Milabs UHT-µCT scanner (Milabs, Utrecht, The Netherlands) of the School of Dentistry using the following parameters: step angle: 0.1 degrees, voltage: 55 kV, current: 0.19 mA, exposure time: 75 ms. Scans were exported as NII files, reconstructed with 20 µm voxel size, and analyzed using 3D Slicer (https://www.slicer.org/) (version 5.0.3) for quantification of the vasculature volume and total lung volume.

Exercise training

Acclimatization lasted a total of 10 days running on a treadmill (Columbus Instruments, Mouse Rat Exercise 3/6 Treadmill 140645 220) at a speed of 5-8 m/min at an incline of 0 each day. Starting at 10 meters, the distance ran will be increased by 5 meters each day. An encouraging prodding stimulus was used to facilitate learning of running behavior. Endurance testing entailed each rat running at 6 m/min for 10 minutes. Every 10 meters, the speed is increased by 1 m/min until the maximum speed of 10 m/min. Thereupon, the rats will continue running, and the end of their endurance test is marked by adamant resistance to running after 5 consecutive prods to their backside.

References

1. Nadelmann ER, et al. Isolation of Nuclei from Mammalian Cells and Tissues for Single-Nucleus Molecular Profiling. *Curr Protoc.* 2021;1(5):e132.
2. Litvinukova M, et al. Cells of the adult human heart. *Nature.* 2020;588(7838):466-72.
3. Reichart D, et al. Pathogenic variants damage cell composition and single cell transcription in cardiomyopathies. *Science.* 2022;377(6606):eabo1984.
4. Dominguez Conde C, et al. Cross-tissue immune cell analysis reveals tissue-specific features in humans. *Science.* 2022;376(6594):eabl5197.
5. Gillich A, et al. Capillary cell-type specialization in the alveolus. *Nature.* 2020;586(7831):785-9.
6. Adams TS, et al. Single-cell RNA-seq reveals ectopic and aberrant lung-resident cell populations in idiopathic pulmonary fibrosis. *Sci Adv.* 2020;6(28):eaba1983.
7. Koenitzer JR, et al. Single-Nucleus RNA-Sequencing Profiling of Mouse Lung. Reduced Dissociation Bias and Improved Rare Cell-Type Detection Compared with Single-Cell RNA Sequencing. *Am J Respir Cell Mol Biol.* 2020;63(6):739-47.
8. Chavkin NW, et al. Endothelial cell cycle state determines propensity for arterial-venous fate. *Nat Commun.* 2022;13(1):5891.
9. Sindi HA, et al. Therapeutic potential of KLF2-induced exosomal microRNAs in pulmonary hypertension. *Nat Commun.* 2020;11(1):1185.
10. Mosmann T. Rapid colorimetric assay for cellular growth and survival: application to proliferation and cytotoxicity assays. *J Immunol Methods.* 1983;65(1-2):55-63.

11. Kojima H, et al. Detection and imaging of nitric oxide with novel fluorescent indicators: diaminofluoresceins. *Anal Chem.* 1998;70(13):2446-53.
12. Justus CR, et al. Transwell In Vitro Cell Migration and Invasion Assays. *Methods Mol Biol.* 2023;2644:349-59.
13. Liang CC, et al. In vitro scratch assay: a convenient and inexpensive method for analysis of cell migration in vitro. *Nat Protoc.* 2007;2(2):329-33.
14. Crowley LC, et al. Quantitation of Apoptosis and Necrosis by Annexin V Binding, Propidium Iodide Uptake, and Flow Cytometry. *Cold Spring Harb Protoc.* 2016;2016(11).
15. Namkung Y, et al. Monitoring G protein-coupled receptor and beta-arrestin trafficking in live cells using enhanced bystander BRET. *Nat Commun.* 2016;7:12178.
16. Avet C, et al. Effector membrane translocation biosensors reveal G protein and betaarrestin coupling profiles of 100 therapeutically relevant GPCRs. *Elife.* 2022;11.
17. Patel VB, et al. PI3Kalpha-regulated gelsolin activity is a critical determinant of cardiac cytoskeletal remodeling and heart disease. *Nat Commun.* 2018;9(1):5390.
18. Chen X, et al. Pharmacological and cell-specific genetic PI3Kalpha inhibition worsens cardiac remodeling after myocardial infarction. *J Mol Cell Cardiol.* 2021;157:17-30.
19. Schock-Kusch D, et al. Transcutaneous measurement of glomerular filtration rate using FITC-sinistrin in rats. *Nephrol Dial Transplant.* 2009;24(10):2997-3001.

Supplemental Figures

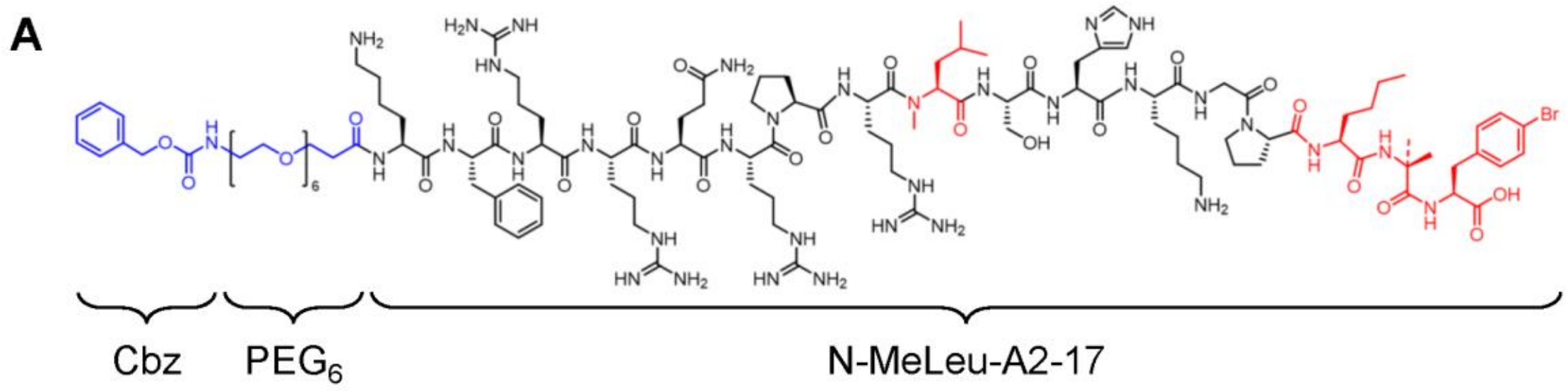

B

	Gαi1	Gαi2	Gαi3	GαoB	β-arr1	β-arr2	Gαq	Gα11	Gαs
A17	9.51 ± 0.04	9.65 ± 0.03	9.26 ± 0.05	9.44 ± 0.02	8.32 ± 0.02	8.33 ± 0.02	NA	NA	NA
AA	9.773 ± 0.05	9.87 ± 0.04	9.57 ± 0.06	9.74 ± 0.04	8.64 ± 0.04	8.65 ± 0.03	NA	NA	NA

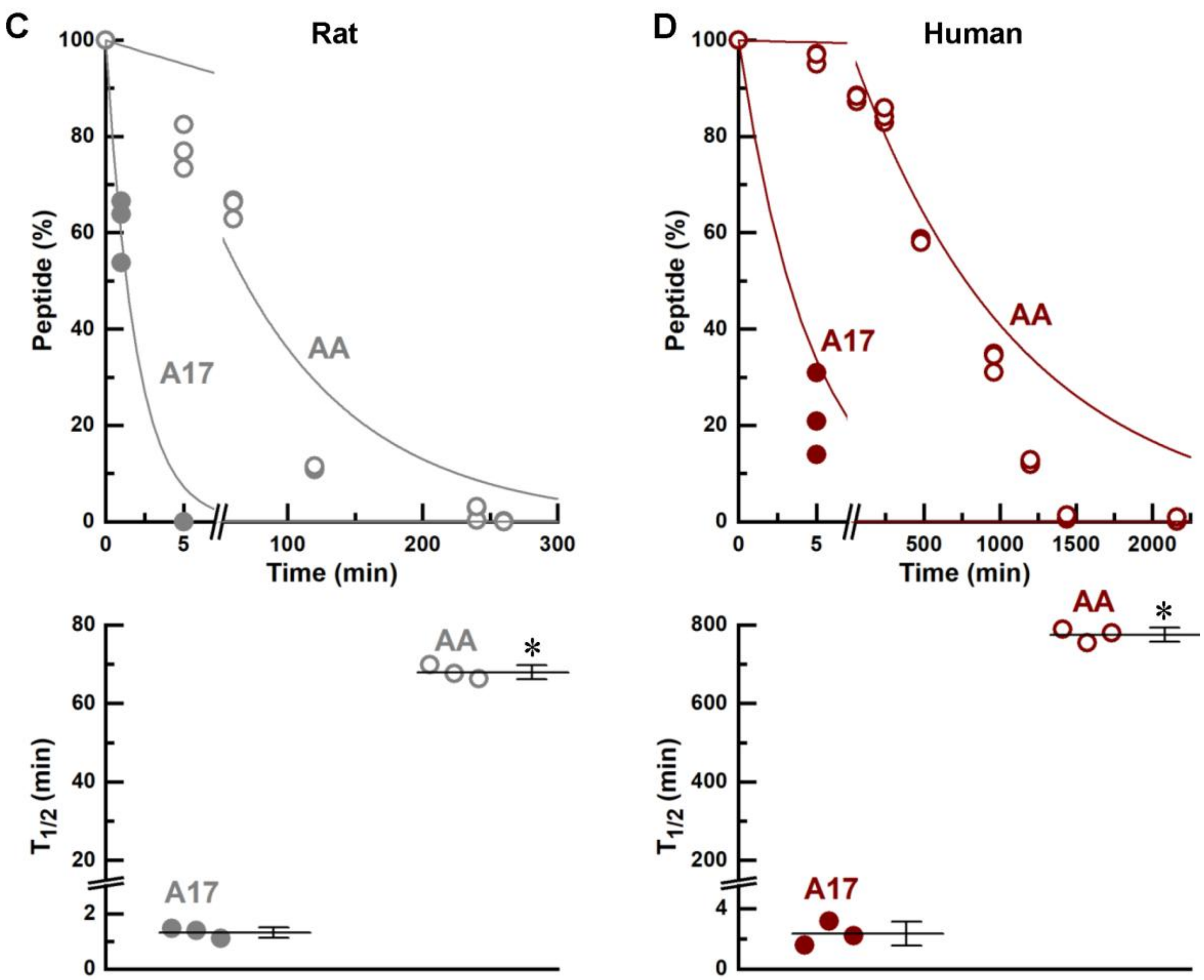

Supplemental Figure 1. **Apelin-17 analog (CbzPEG6-NMeLeu-A2-17) structure, G-protein specificity, and stability in rat and human plasma**. (**A**) Apelin-17 analog chemical structure. (**B**) pEC50 of activation of G-proteins by native apelin-17 (A17) and apelin-17 analog (AA) in bioluminescence resonance energy transfer assay in HEK293 cells. (**C**) Native apelin-17 (A17) and apelin-17 analog (AA) stability in rat heparinized plasma: time course of decay (top) and half-life ($T_{1/2}$) (bottom). (**D**) Native apelin-17 (A17) and apelin-17 analog (AA) stability in human heparinized plasma: time course of decay (top) and half-life ($T_{1/2}$) (bottom). Data are mean ± SD.

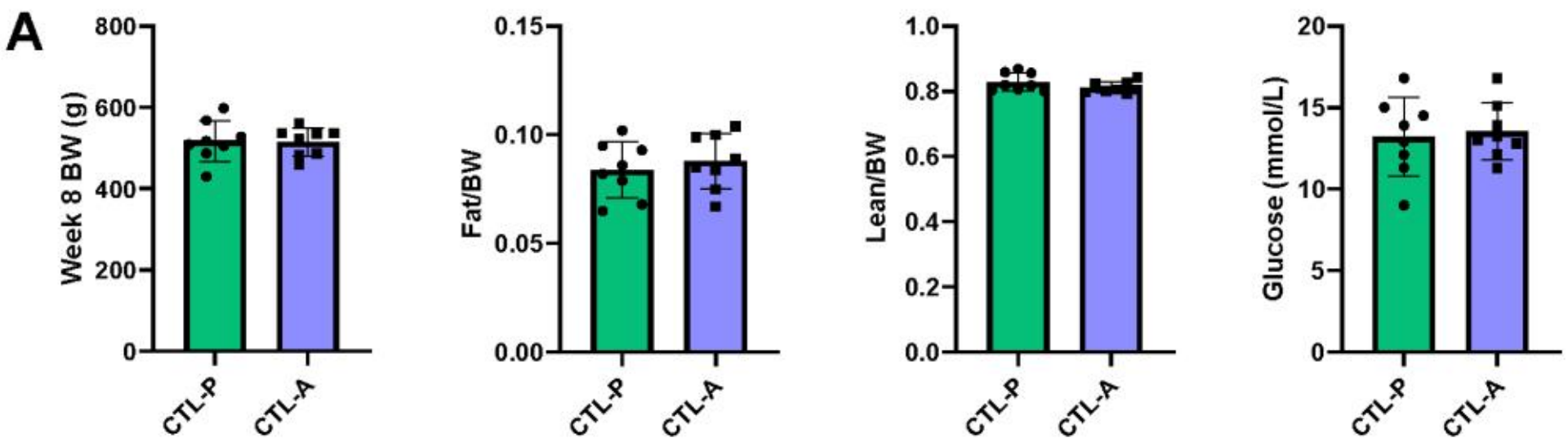

B

	RR	PR	QTcB	QTcF	SV	RV FAC	TAPSE	PAAT/PAET
	ms	ms	ms	ms	µL	%	mm	
CTL-P	190.6 ± 18.4	54.3 ± 4.77	64.2 ± 4.06	66.8 ± 4.72	254.3 ± 36.8	59.0 ± 4.40	2.88 ± 0.31	0.44 ± 0.07
CTL-A	197.9 ± 16.9	54.9 ± 5.35	62.4 ± 3.26	65.4 ± 3.76	265.7 ± 27.5	59.2 ± 8.16	2.80 ± 0.53	0.44 ± 0.06

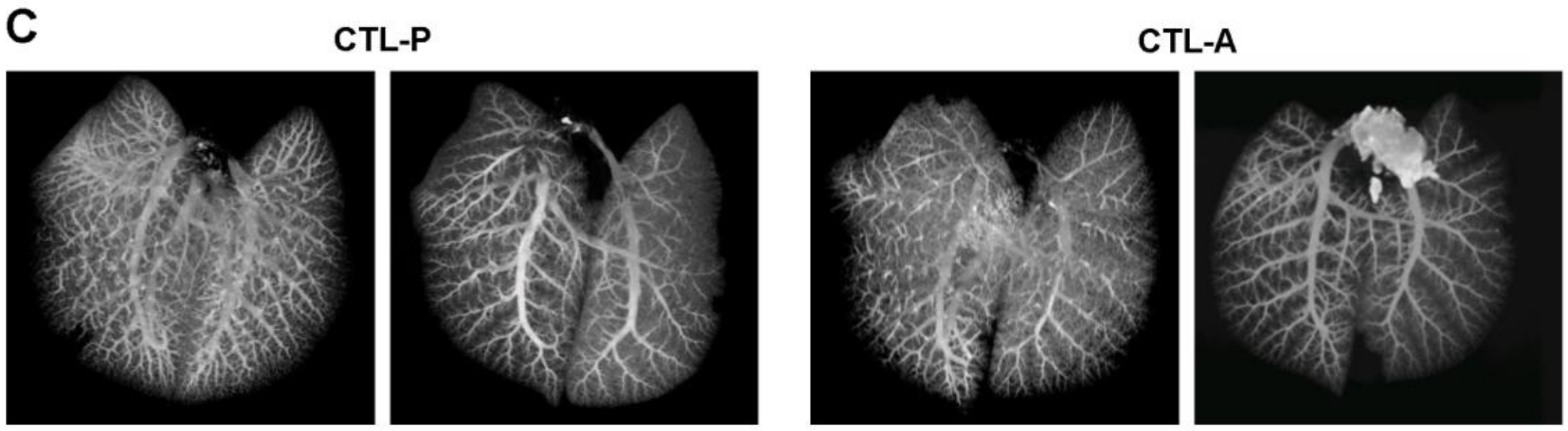

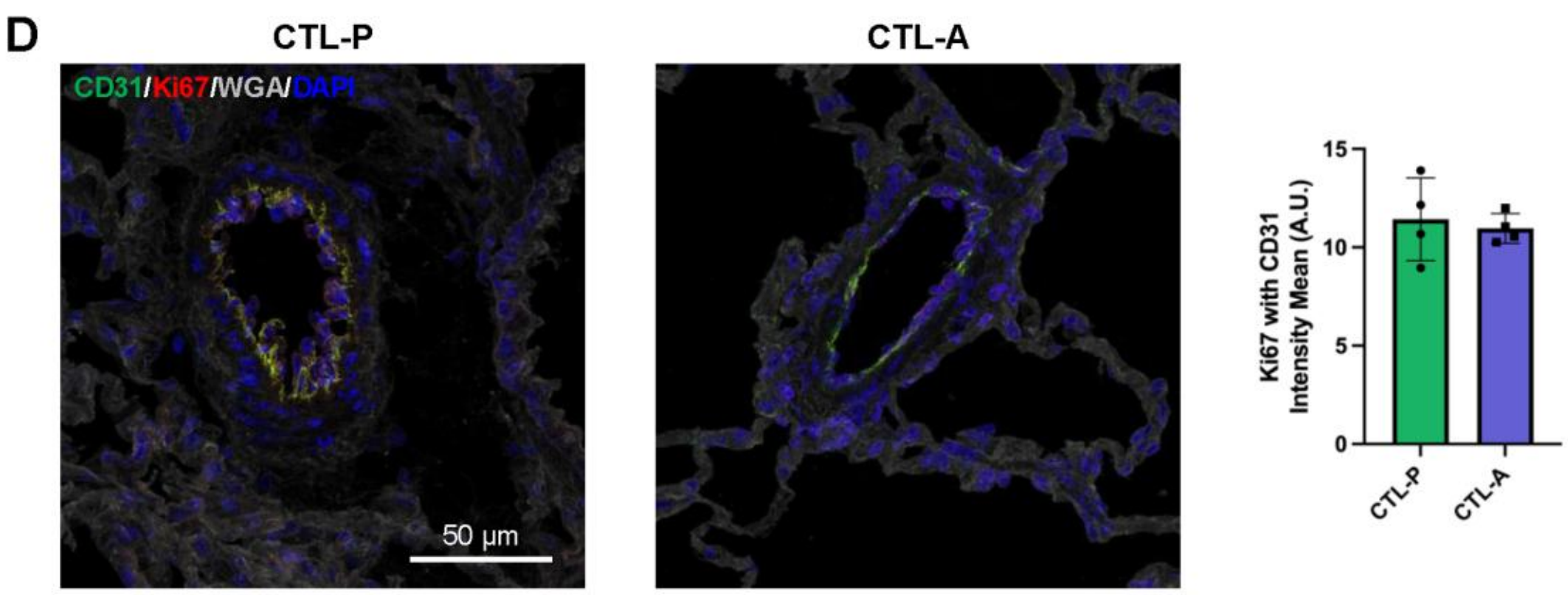

Supplemental Figure 2. **Metabolism, cardiac function, and angiogenesis in rats treated with apelin analog**. (**A**) Body weight at 8-week time point; echoMRI body composition: fat mass to body weight (Fat/BW) and lean mass to body weight (Lean/BW); and random blood glucose at the time of tissue collection; n=8 per group. (**B**) ECG and echocardiography: RR interval duration; PR interval duration; QTcB, QT interval duration with Bazett correction; QTcF, QT interval duration with Fridericia correction; SV, stroke volume; RV FAC, right ventricular fractional area change; TAPSE, tricuspid annular plane systolic excursion; PAAT, pulmonary artery acceleration time; PAET, pulmonary artery ejection time; n=8 per group. (**C**) Images of micro-computed tomography of the lungs following *ex vivo* perfusion of barium contrast into the main pulmonary artery. (**D**) Representative images of immunofluorescent co-staining of lung sections with CD31 (green), Ki67 (red), wheat germ agglutinin (WGA; grey), and DAPI (blue) and quantification of Ki67 positive staining. Eight images were taken from each lung; n=4 lungs per group for quantification. CTL-P, control rats treated with placebo (saline); CTL-A, control rats treated with apelin-17 analog. Data are mean ± SD.

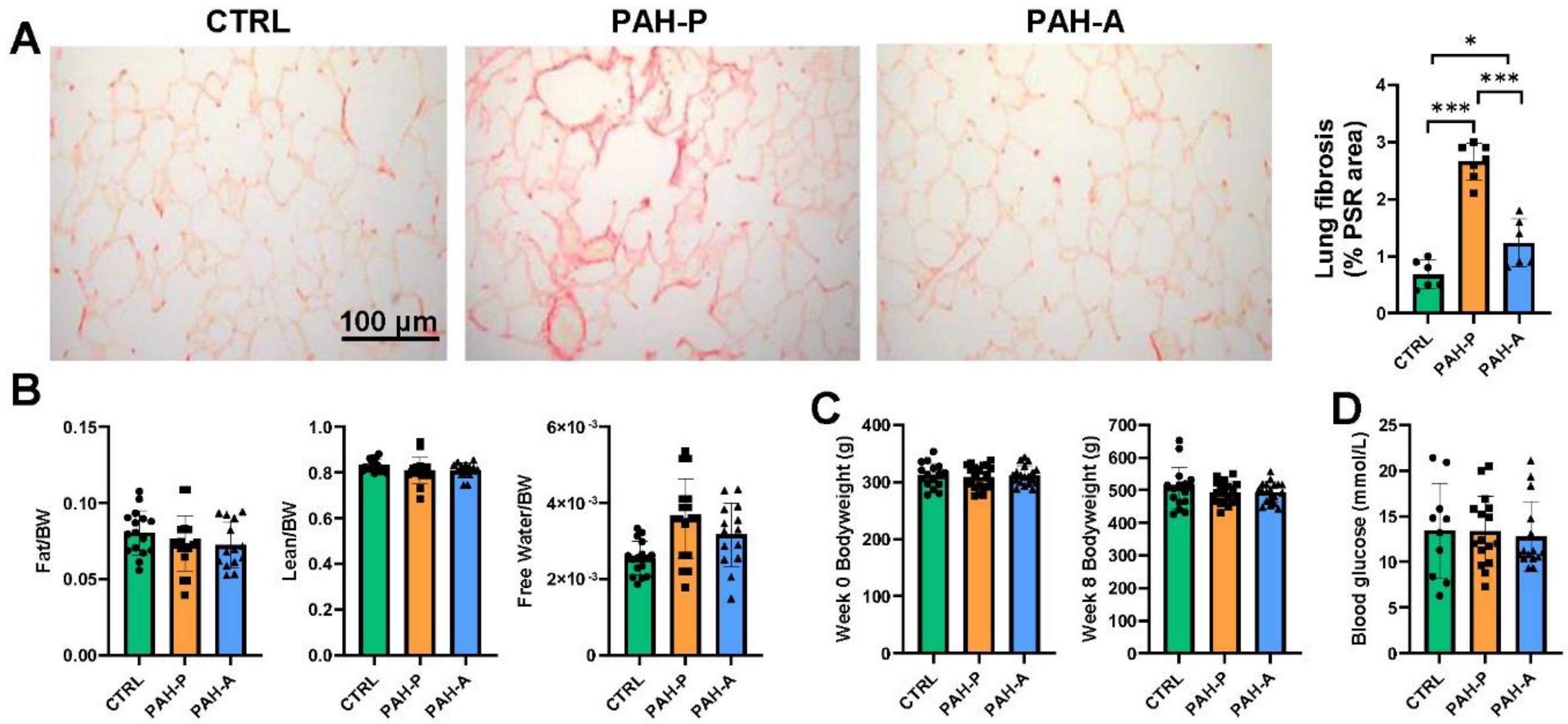

Supplemental Figure 3. **Pulmonary fibrosis, body composition, and blood glucose levels**. (**A**) Representative images of picrosirius red staining of lungs and quantification (n = 6). (**B**) EchoMRI body composition: fat mass to body weight (Fat/BW), lean mass to body weight (Lean/BW), and free water mass to body weight (Free Water/BW); n=14-18. (**C**) Rat body weight at Week 0 and Week 8; n=14-18. (**D**) Random blood glucose levels at the time of tissue collection; n=14-18. CTRL, control rats; PAH-P, pulmonary arterial hypertension (PAH) rats treated with placebo; PAH-A, PAH rats treated with apelin-17 analog. Data are mean ± SD.

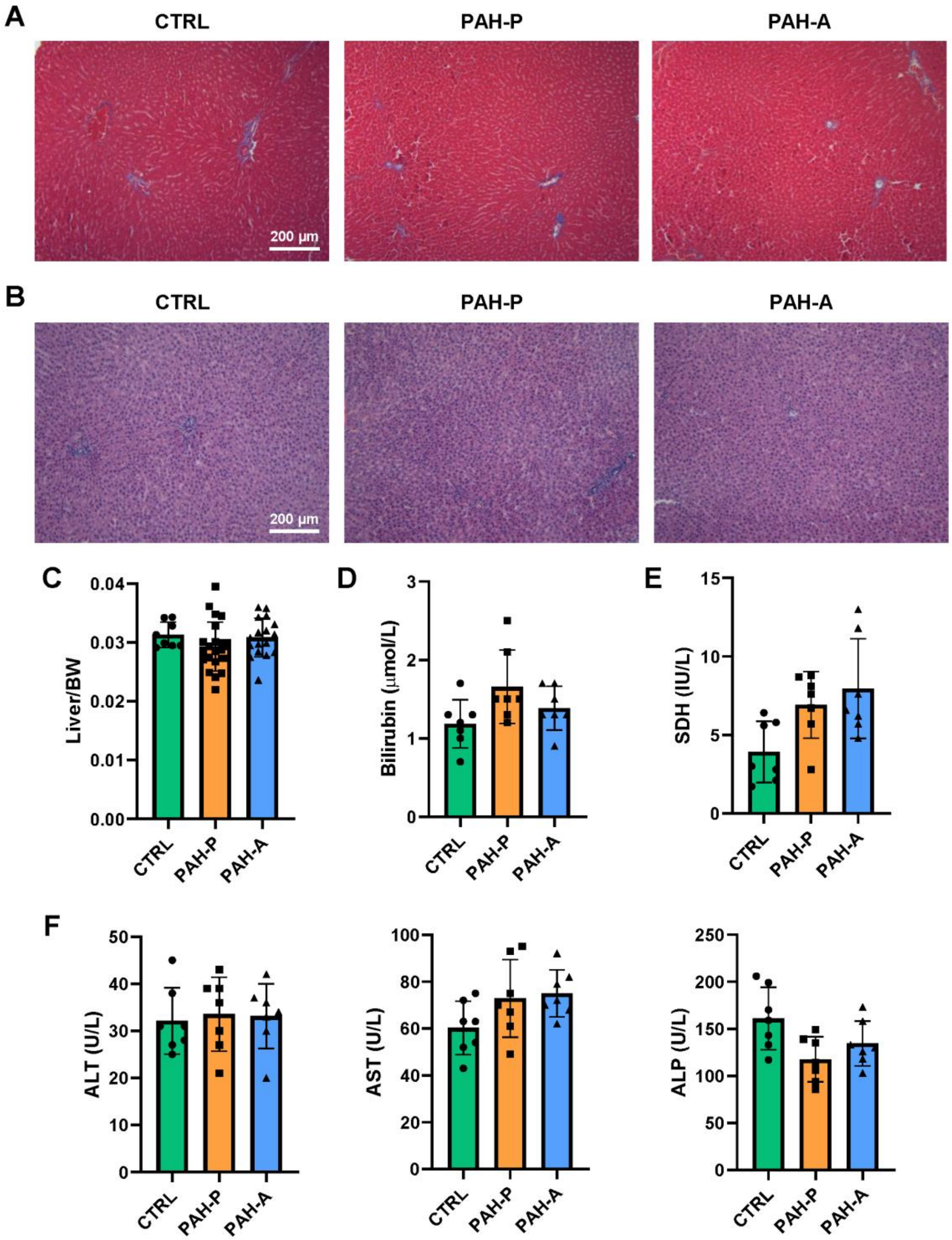

Supplemental Figure 4. **Liver histology and function in sugen-hypoxia model of PAH and after treatment with apelin-17 analog**. (**A**) Representative images of the Masons trichrome staining of liver sections. (**B**) Representative images of the hematoxylin and eosin staining of liver sections. (**C**) Liver mass to body weight (BW) ratio (n=8-20). (**D**) Bilirubin levels in the plasma (n=6). (**E**) Sorbitol dehydrogenase (SDH) activity in the plasma (n=6). (**F**) Activities of alanine transaminase (ALT), aspartate aminotransferase (AST), and alkaline phosphatase (ALP) activities in plasma (n=6). CTRL, control rats; PAH-P, PAH (pulmonary arterial hypertension) rats treated with placebo; PAH-A, PAH rats treated with apelin-17 analog. Data are mean±SD.

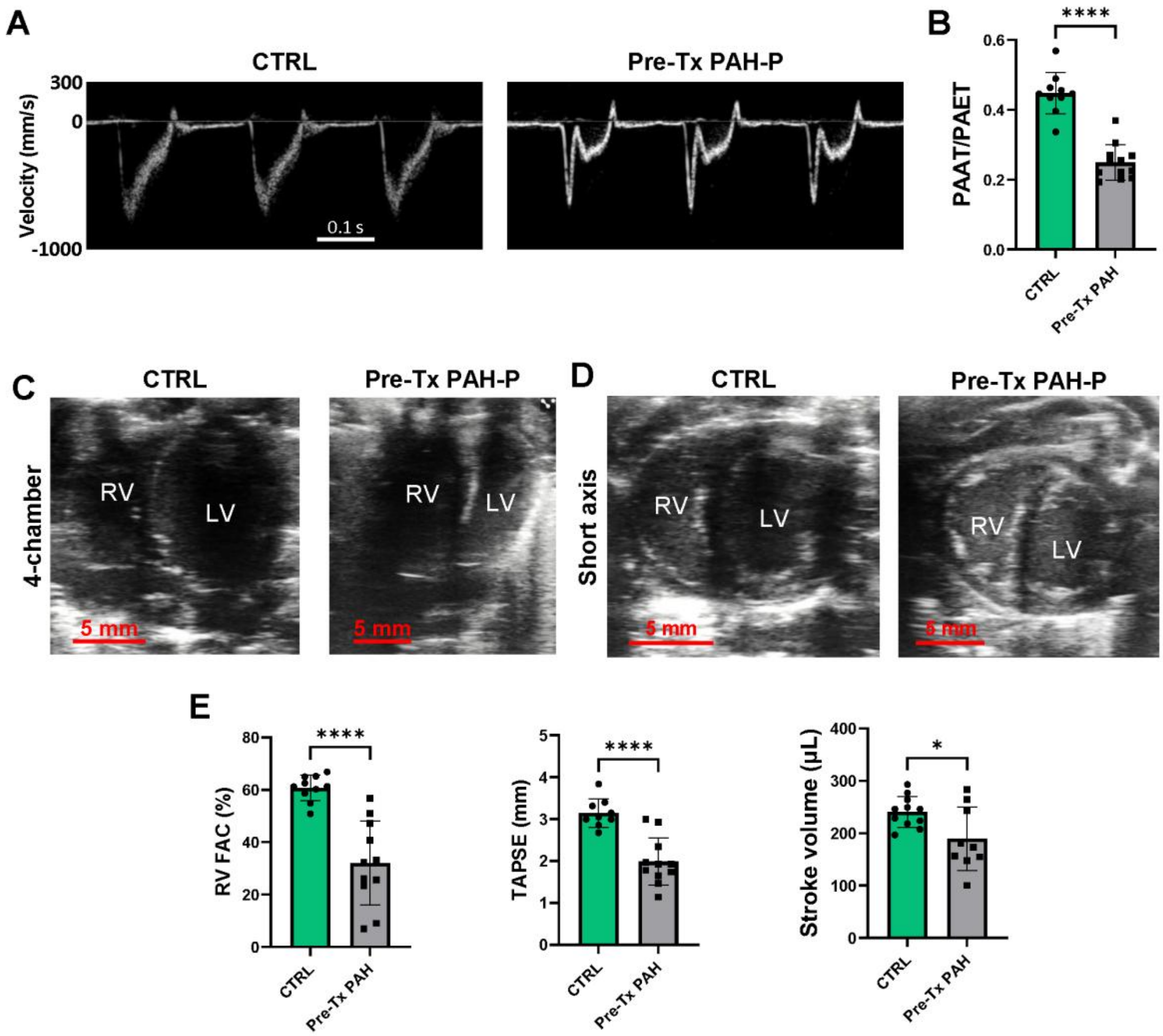

Supplemental Figure 5. **Echocardiographic assessment of rats before administration of placebo or apelin-17 analog**. (**A**) Representative pulse-wave Doppler images of the pulmonary arterial flow. (**B**) Quantification of the ratio of pulmonary artery acceleration time to pulmonary artery ejection time (n=10-12). (**C**) Representative B-mode images of 4-chamber views. (**D**) Representative B-mode image of short axis view. (**E**) Right ventricle fractional area change (RVFAC), tricuspid annular plane systolic excursion (TAPSE), and LV stroke volume (n=10-12). CTRL, control rats; Pre-Tx PAH, PAH (pulmonary arterial hypertension) rats before treatment with placebo or apelin-17 analog (at 5 week). Data are mean±SD.

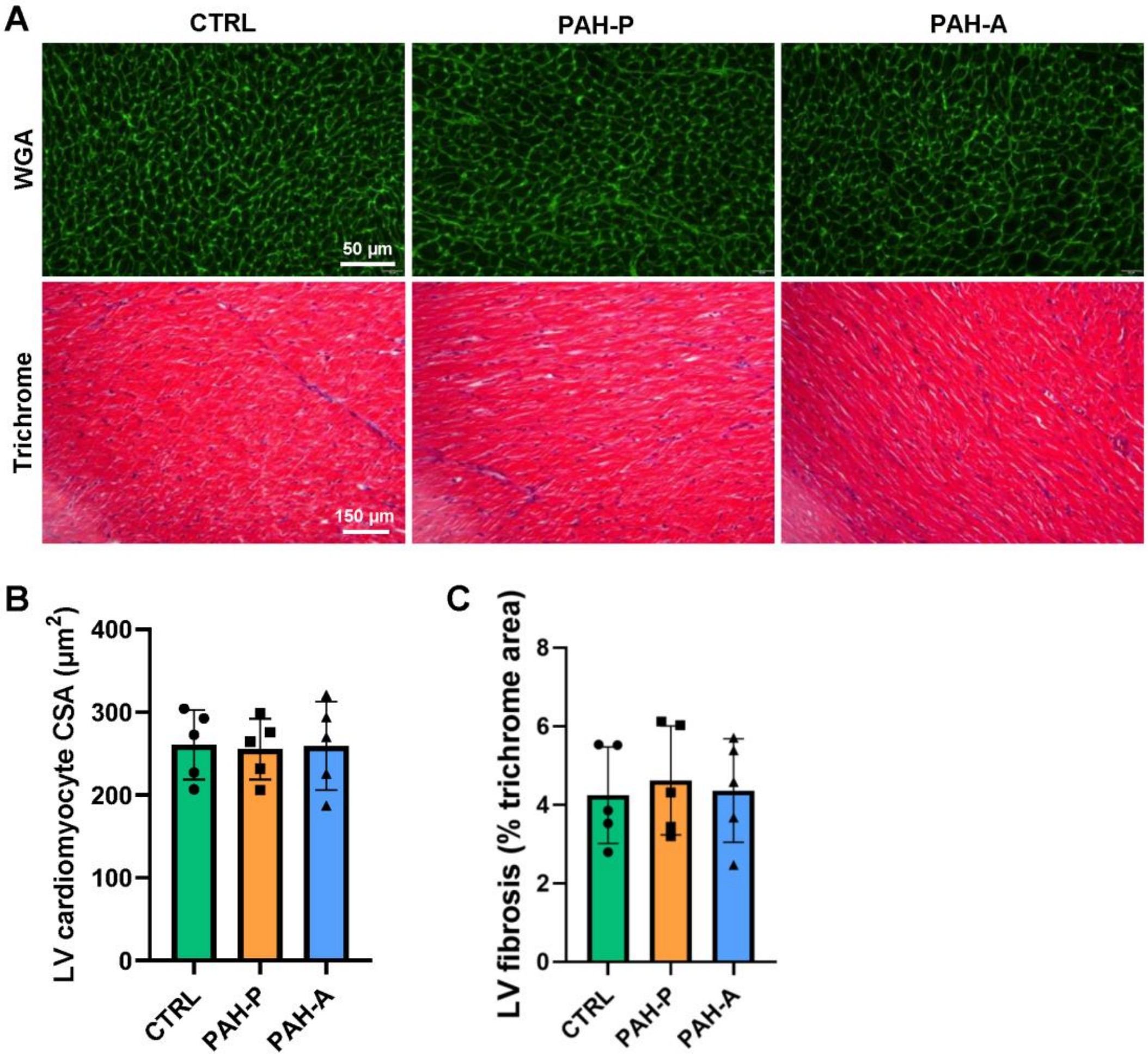

Supplemental Figure 6. **Absence of hypertrophy and fibrosis in the rat LVs**. (**A**) Representative images of wheat germ agglutinin (WGA) (top) and Masson's trichrome staining (bottom) of rat LV sections. (**B**) LV cardiomyocyte cross sectional area (CSA) quantified from WGA staining of sections of rat LVs (n=5 per group). (**C**) Percentage of blue trichrome strain of total LV tissue area, quantified from Masson's trichrome staining of sections of rat LVs (n=5 per group). CTRL, control rats; PAH-P, PAH (pulmonary arterial hypertension) rats treated with placebo; PAH-A, PAH rats treated with apelin-17 analog. Data are mean ± SD.

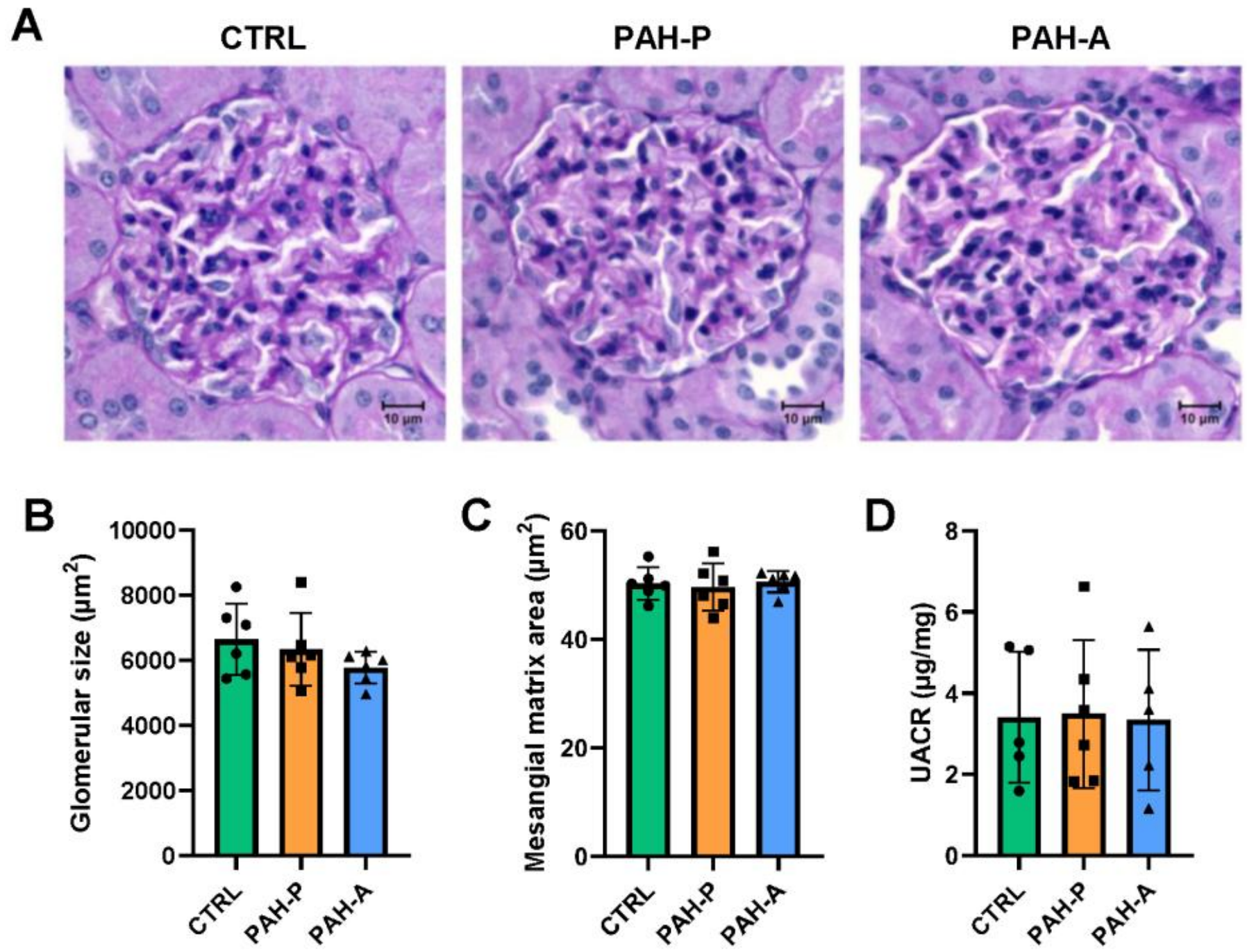

Supplemental Figure 7. **Glomerular morphology and function**. (**A**) Representative images of periodic acid Schiff (PAS) staining of the kidney sections. (**B**) Average glomerular size quantified from PAS staining of sections of rat kidneys (n=6 per group). (**C**) Mesangial matrix area quantified from PAS staining of sections of rat kidneys (n=6 per group). (**D**) UACR (urine albumin-creatinine ratio) from random urine samples (n=5 per group). CTRL, control rats; PAH-P, pulmonary arterial hypertension (PAH) rats treated with placebo; PAH-A, PAH rats treated with apelin-17 analog. Data are mean ± SD.

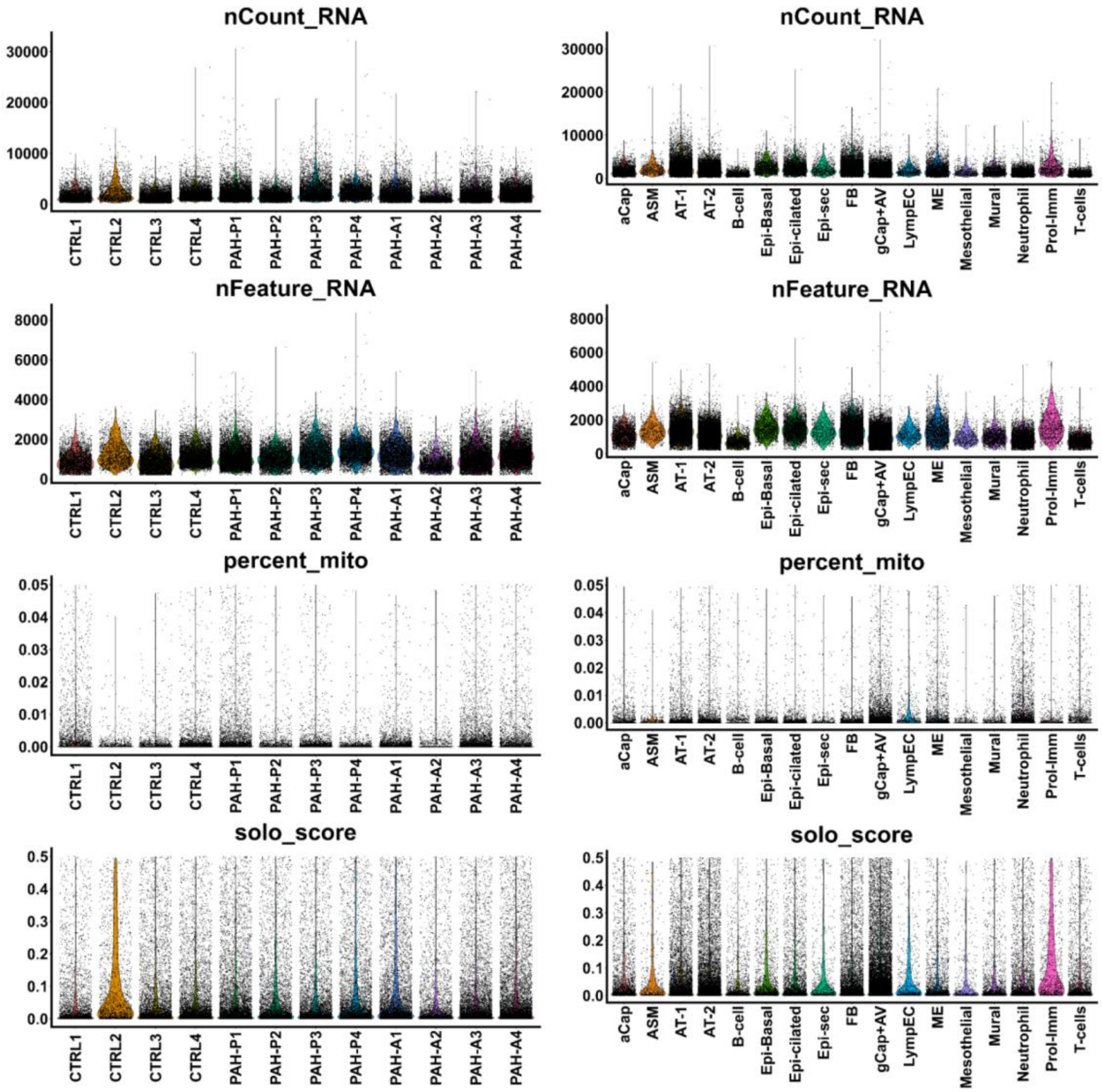

Supplemental Figure 8. **Single nuclei RNA sequencing quality control clustering and filtering of lung nuclei**. The number of UMIs (nCount_RNA), number of genes (nFeature_RNA), fraction of UMIs that are mitochondrial (percent mito), and doublet score (solo_score) for each lung sample and cell type; UMI, unique molecular identifier.

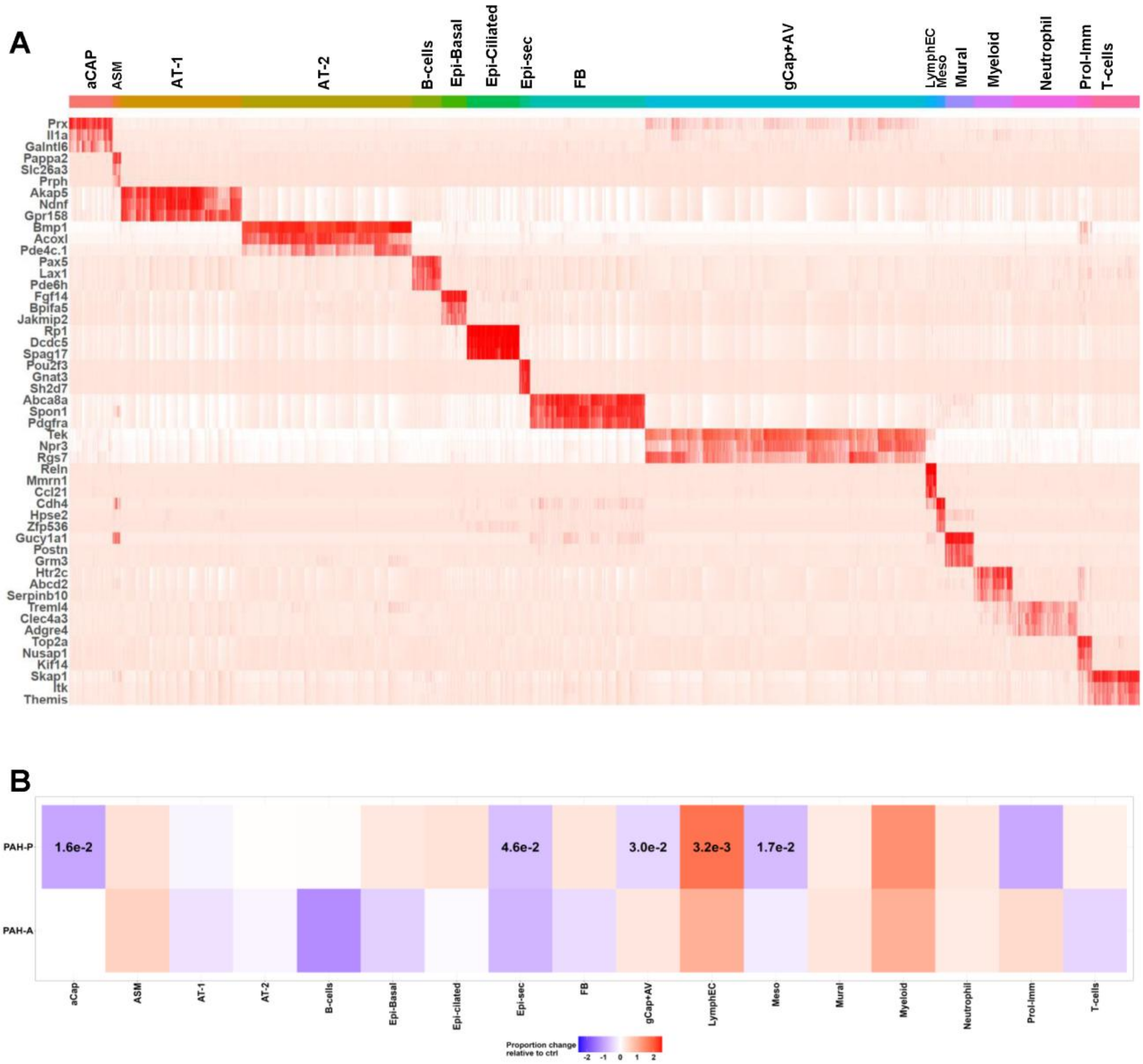

Supplemental Figure 9. **Pulmonary cell type markers and proportion change of pulmonary cell types**. (**A**) Heatmap of expression levels of pulmonary cell type marker genes. (**B**) Heatmap of changes in proportions for all pulmonary cell types (p values are shown for significant changes).

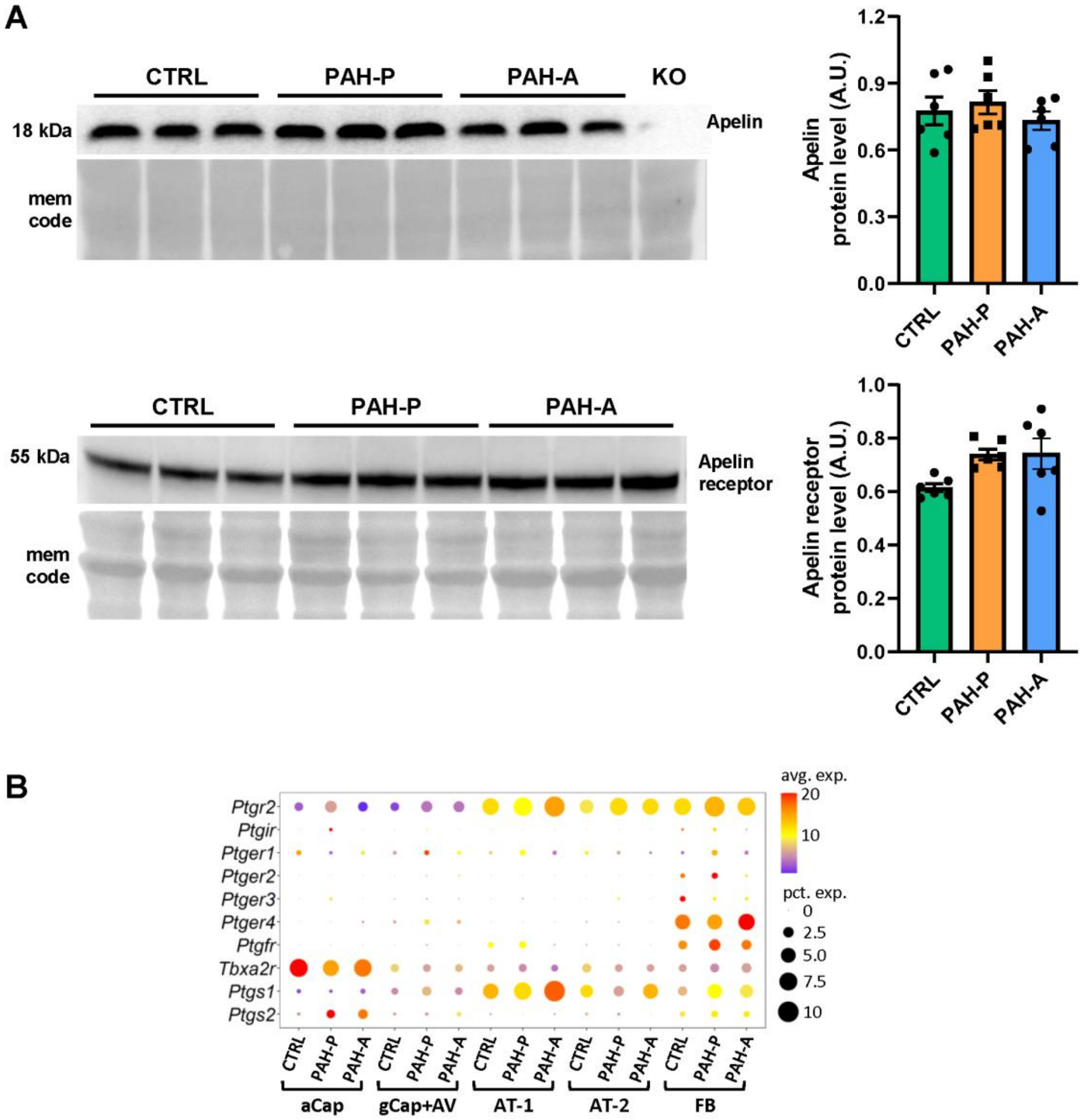

Supplemental Figure 10. **Apelin and apelin receptor levels and prostaglandin pathways in the lungs**. (**A**) Representative Western blots for apelin and apelin receptor in lung tissue and the quantification of apelin and apelin receptor levels for CTRL, PAH-P, and PAH-A (n=6 animals). (**B**) Dot plot of the selected genes showing average expression (purple (low), yellow (mid), and red (high)) and fraction of cell expressing (size of the dot) for major cell types (aCap, gCap+AV, AT-1, AT-2, and FB) across treatment conditions. CTRL, control rats; PAH-P, pulmonary arterial hypertension (PAH) rats treated with placebo; PAH-A, PAH rats treated with apelin analog.

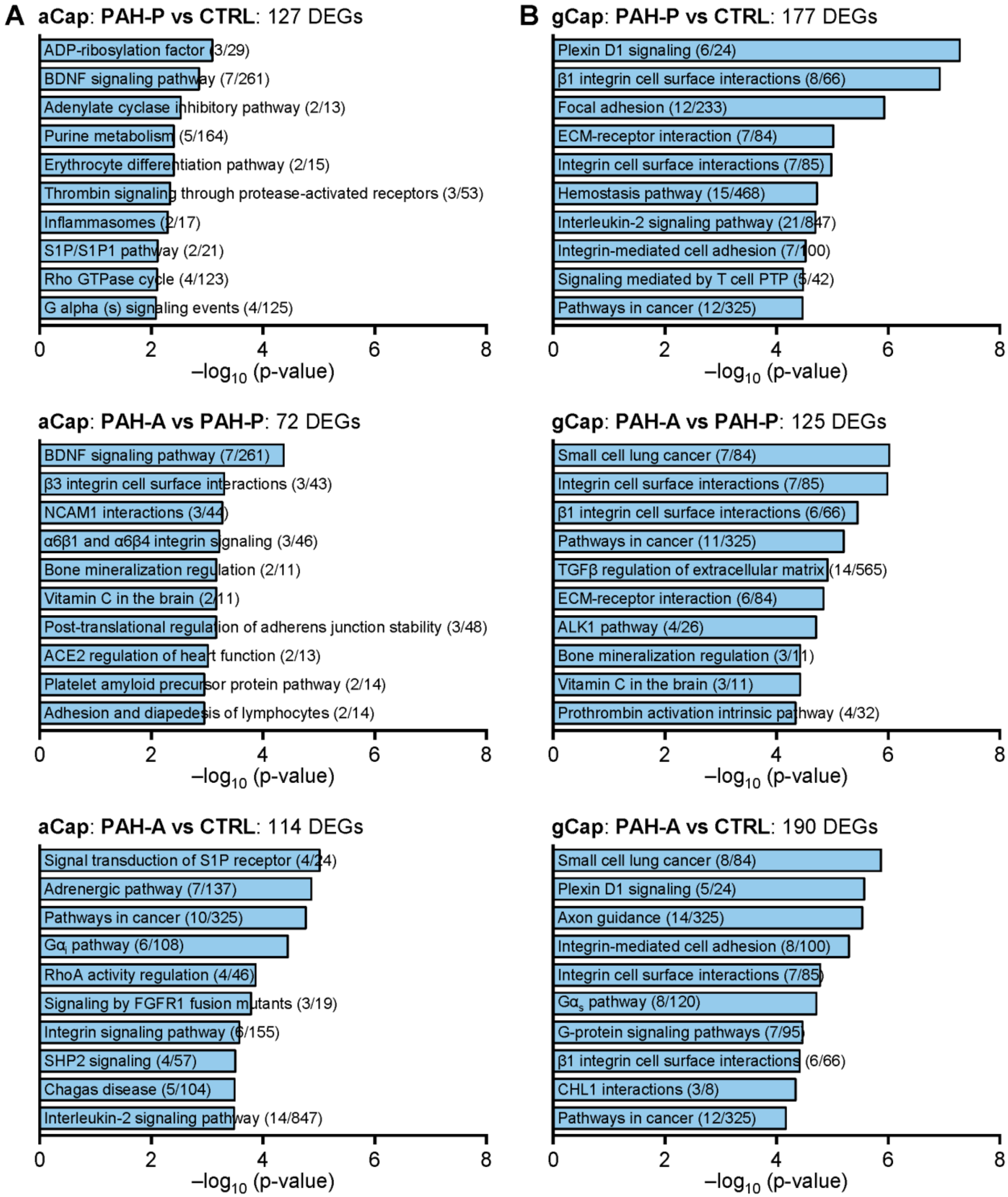

Supplemental Figure 11. **Pathways enrichment analyses of differently expressed genes for aCaps (aerocytes) and gCaps (general endothelial capillary cells)**. (**A**) aCaps: BioPlanet pathway analysis of differentially expressed genes (DEGs) between PAH-P and CTRL, PAH-A and PAH-P, and PAH-A and CTRL in aCaps. (**B**) gCaps: BioPlanet pathway analysis of differentially expressed genes (DEGs) between PAH-P and CTRL, PAH-A and PAH-P, and PAH-A and CTRL rats. CTRL, control rats; PAH-P, pulmonary arterial hypertension (PAH) rats treated with placebo; PAH-A, PAH rats treated with apelin-17 analog.

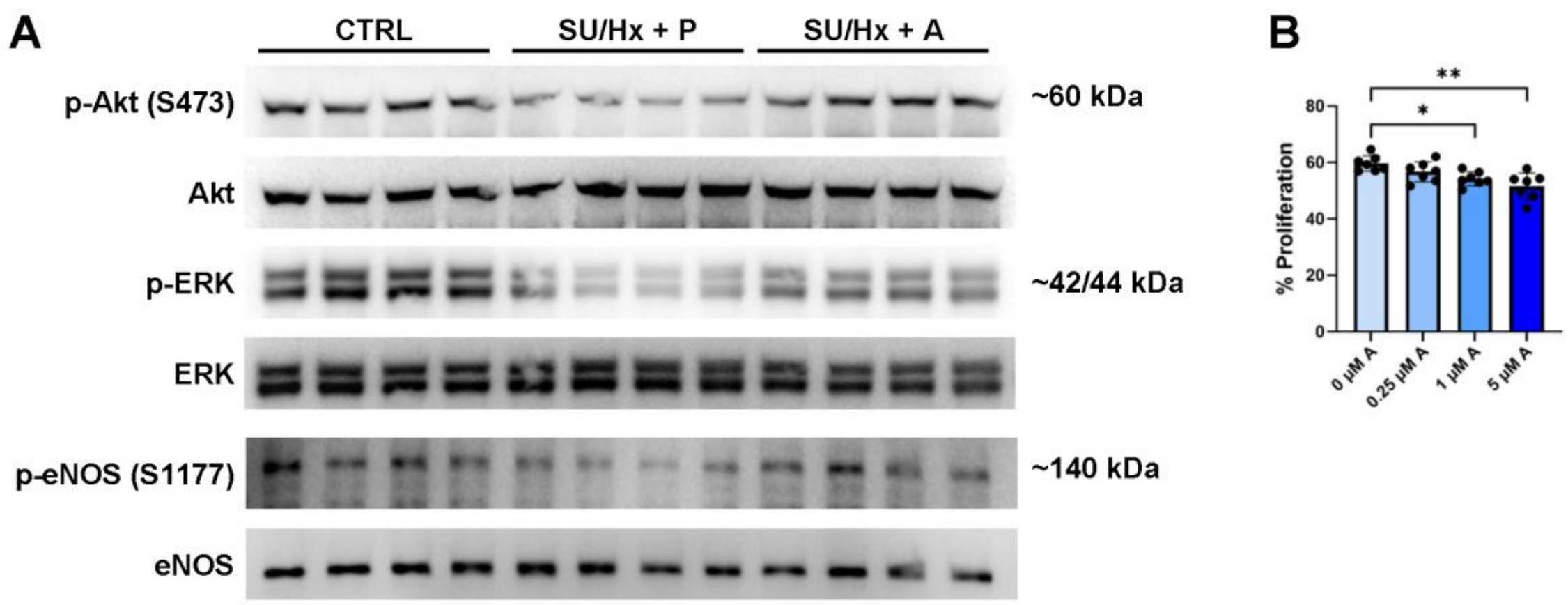

Supplemental Figure 12. **Signaling and proliferation in Human Pulmonary Artery Endothelial Cells (HPAEC)**. (**A**) Immunoblots for p-Akt(S473)/Akt, p-ERK/ERK, and p-eNOS (S1177)/eNOS in HPAECs across treatment groups (n=4 replicates). (**B**) Apelin analog reduces hyperproliferation of HPAECs *in vitro* quantified with flow cytometry (n=7 replicates). CTRL = vehicle control; SU/Hx + P = SU5416/hypoxia + placebo; SU/Hx + A = SU5416/hypoxia + apelin analog. Data are mean ± SD. *p<0.05, **p<0.01, ***p<0.001.

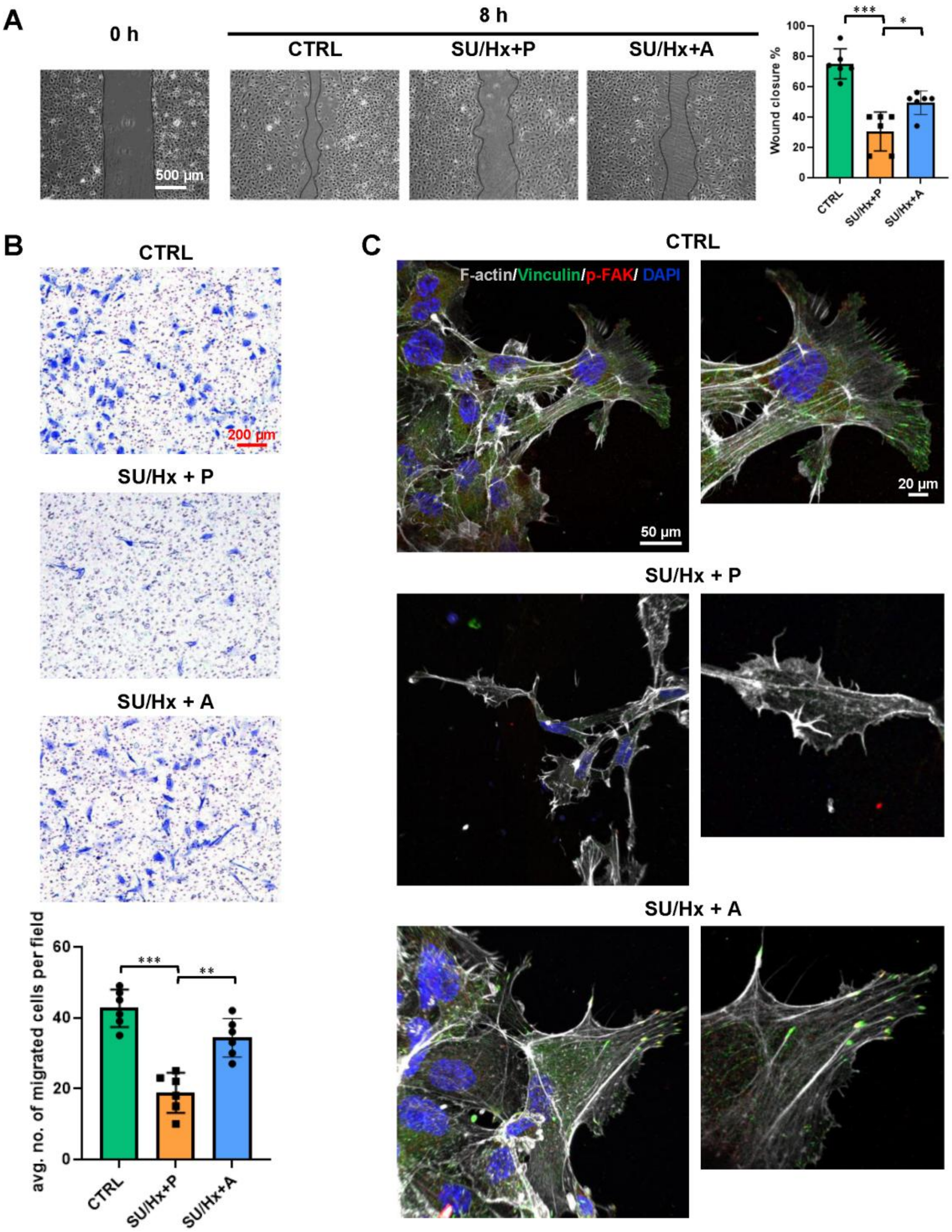

Supplemental Figure 13. **Apelin analog rescues impaired Human Pulmonary Artery Endothelial Cells (HPAEC) migration under simulated PAH conditions**. (**A**) Wound-healing assay in HPAECs: representative images (0 and 8 h) and quantification of wound closure (n=6 replicates). (**B**) Representative transwell membrane images and quantification of migrated cell number per field toward VEGF/FGF-2 chemoattractant gradient (n=6 replicates). (**C**) Representative confocal immunofluorescence images of F-actin (white), vinculin (green), pFAK (red), and DAPI (blue) at the wound edge. CTRL = vehicle control; SU/Hx + P = SU5416/hypoxia + placebo; SU/Hx + A = SU5416/hypoxia + apelin analog. Data are mean ± SD. *p<0.05, **p<0.01, ***p<0.001.

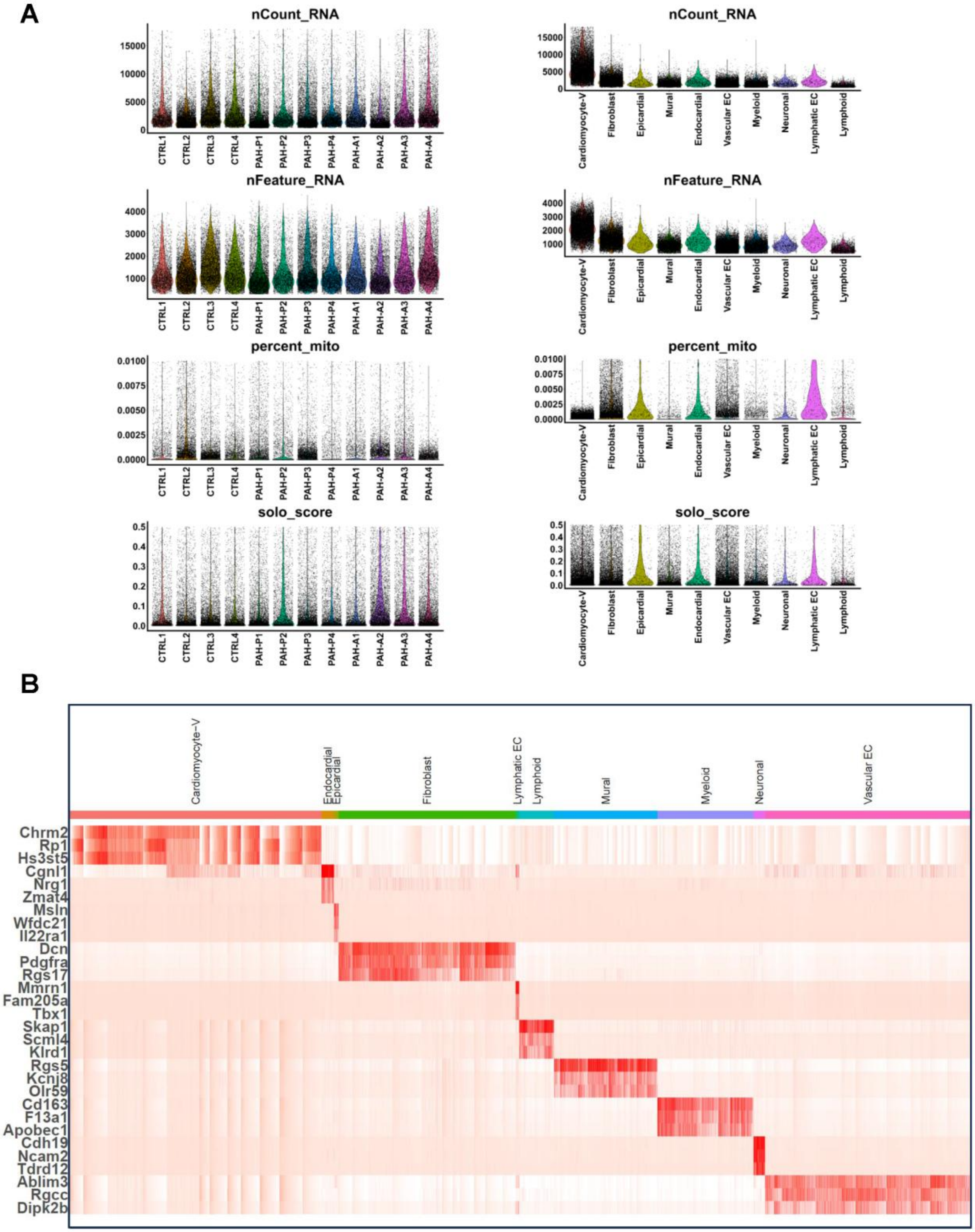

Supplemental Figure 14. **Single nuclei RNA sequencing quality control clustering, filtering, and marker genes in the RV**. (**A**) The number of UMIs (nCount_RNA), number of genes (nFeature_ RNA), fraction of UMIs that are mitochondrial (percent mito), and doublet score (solo_ score) for each RV sample and cell type. **(B)** Heatmap of expression levels of RV cell type marker genes. UMI, unique molecular identifier.

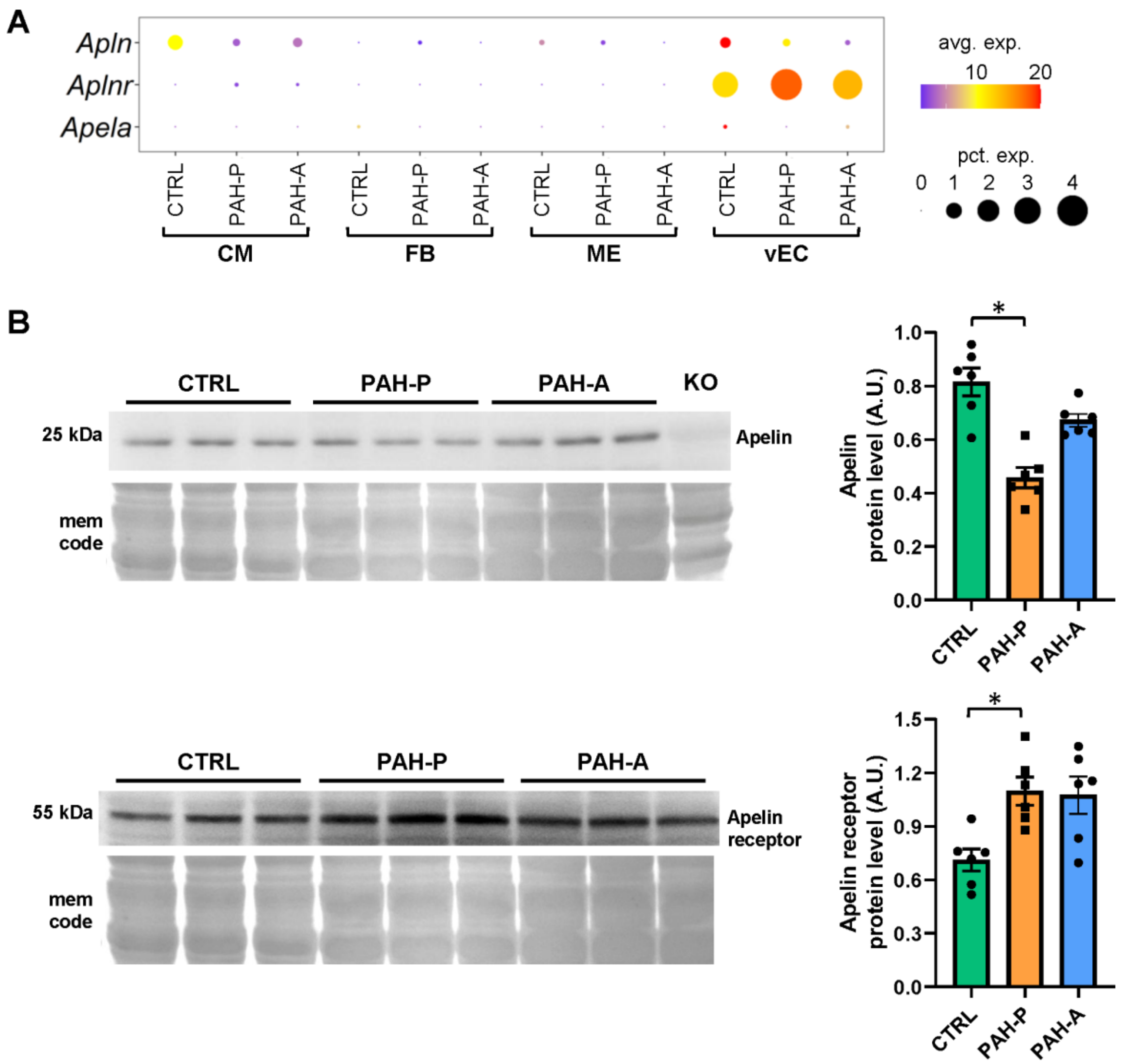

Supplemental Figure 15. **Apelin and apelin receptor in the RV**. (**A**) Dot plot of expression of *Apln*, *Aplnr*, and *Apela* in cardiomyocytes (CM), fibroblasts (FB), myeloid (ME), and vascular endothelial cells (vEC). Average expression corresponds to purple (low), yellow (mid) and red (high) color scale, and proportion of cells expressing specific gene corresponds to size of dot. (**B**) Representative Western blots for apelin and apelin receptor in RV tissue and the quantification of apelin and apelin receptor levels for CTRL, PAH-P, and PAH-A (n=6 animals). CTRL, control rats; PAH-P, pulmonary arterial hypertension (PAH) rats treated with placebo; PAH-A, PAH rats treated with apelin analog.

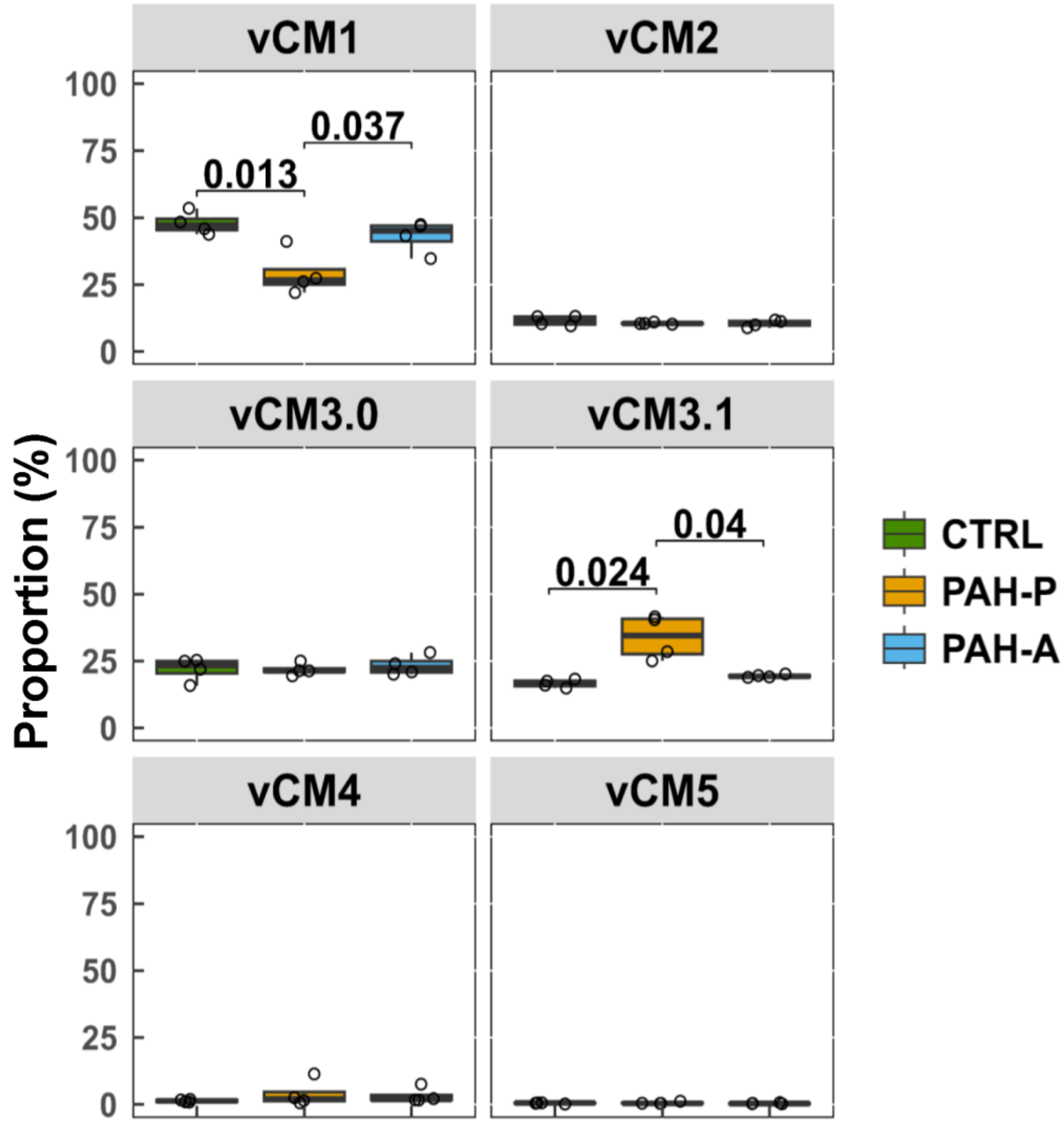

Supplemental Figure 16. **Cardiomyocyte cell state proportions**. Cardiomyocyte cell state proportions across treatment conditions presented as percentage of total number of cardiomyocyte nuclei. CTRL, control rats; PAH-P, pulmonary arterial hypertension (PAH) rats treated with placebo; PAH-A, PAH rats treated with apelin analog.

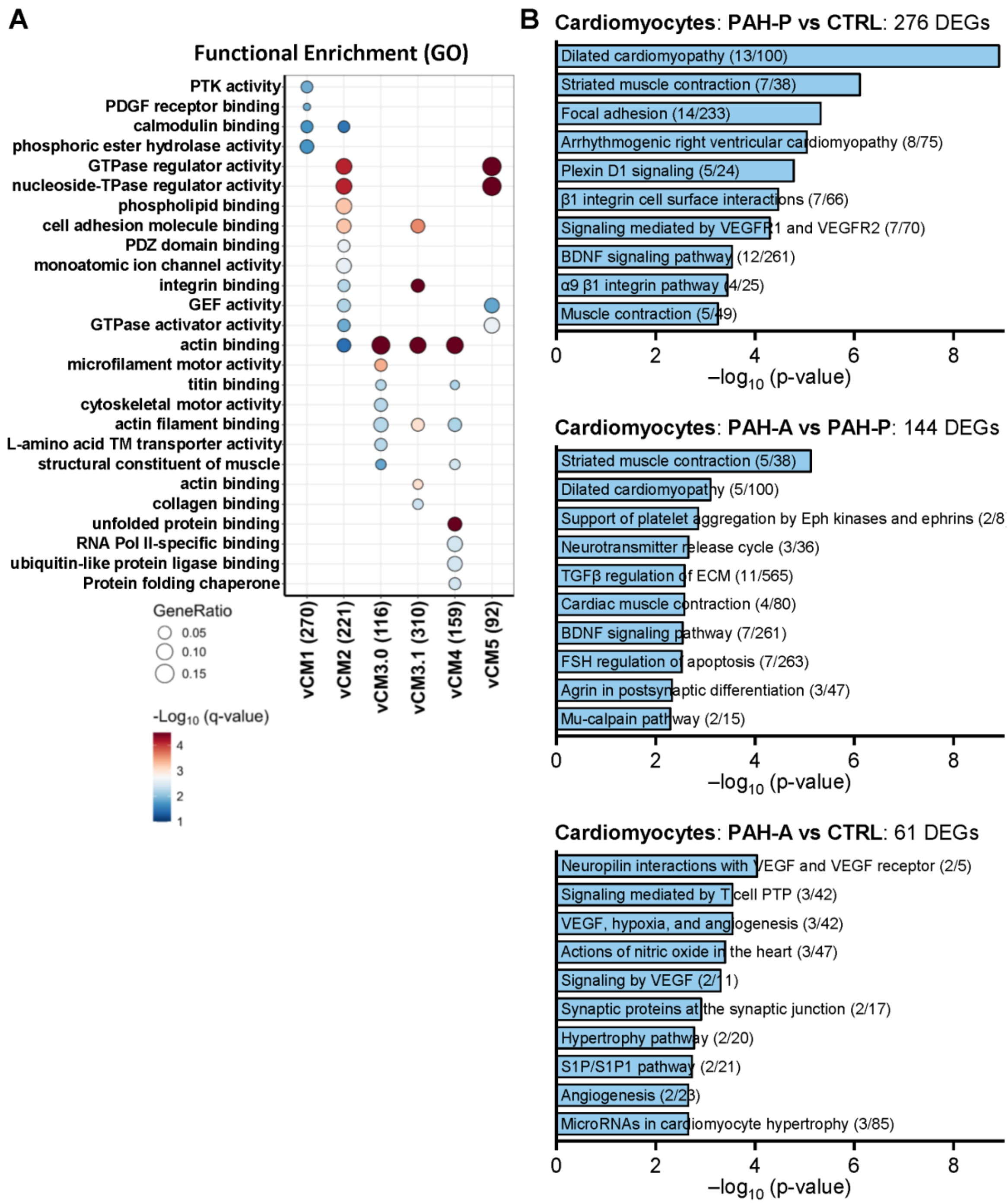

Supplemental Figure 17. **Functional enrichment of marker genes and pathway enrichment analysis of differentially expressed genes in RV cardiomyocytes (pseudo-bulk)**. (**A**) Functional enrichment analysis of cardiomyocyte marker genes to elucidate functional roles of RV cardiomyocyte cell states using Cluster profiler pathway analysis. (**B**) BioPlanet pathway analysis of differentially expressed genes (DEGs) between PAH-P and CTRL, PAH-A and PAH-P, and PAH-A and CTRL rat RV cardiomyocytes. CTRL, control rats; PAH-P, pulmonary arterial hypertension (PAH) rats treated with placebo; PAH-A, PAH rats treated with apelin analog.

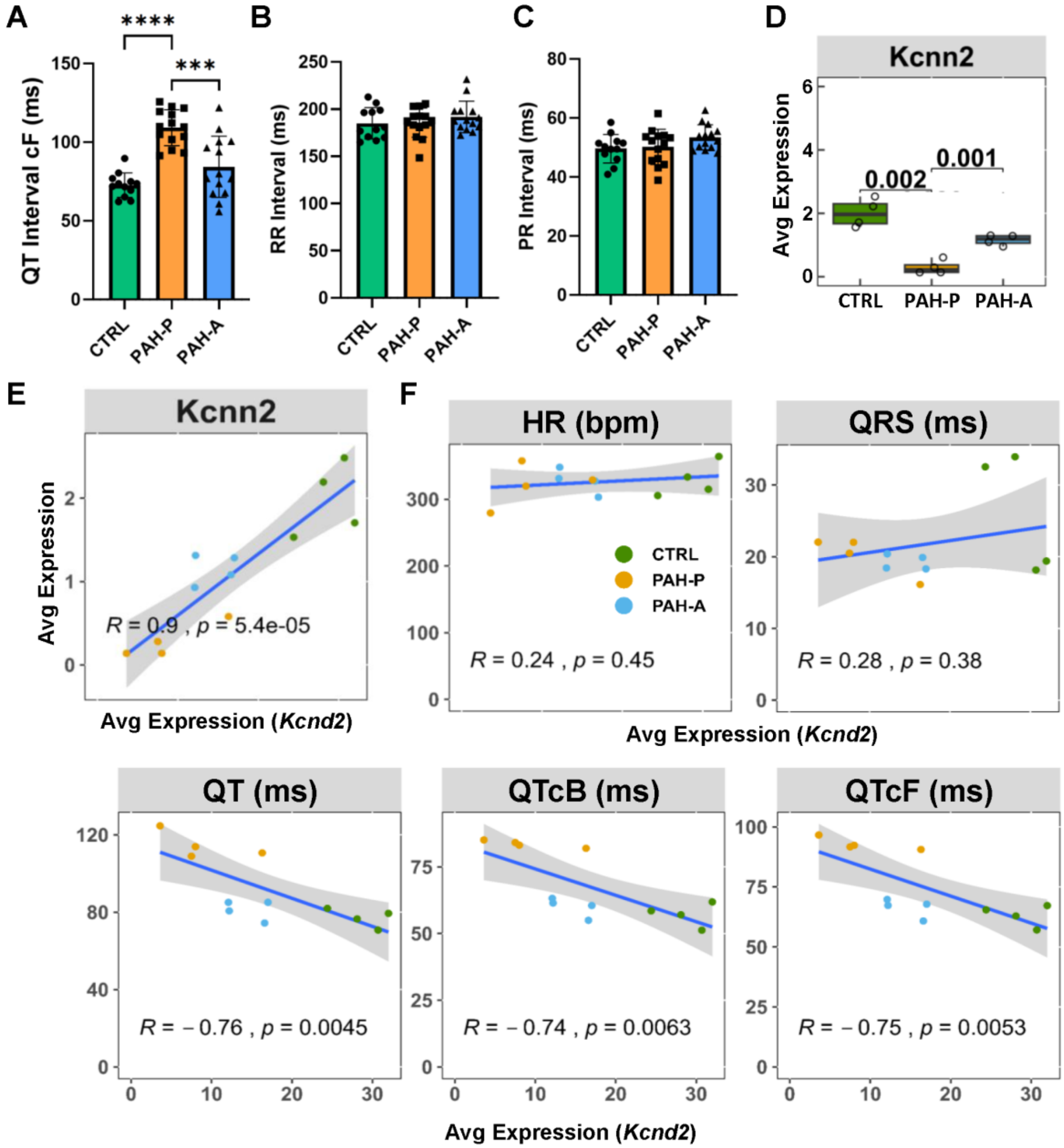

Supplemental Figure 18. **Electrical activity in rat hearts and expression levels of K$^+$ channels Kcnn2 and Kcnd2**. (**A**) QT interval duration with Fridericia's correction. (**B**) RR interval duration. (**C**) PR interval duration. (**D**) Expression of *Kcnn2* for CTRL, PAH-P, and PAH-A. (**E**) Correlation between *Kcnn2* and *Kcnd2* expression levels. (**F**) Correlation of heart rate (HR), QRS interval, QT interval, QT interval with Bazett correction (QTcB), and QT interval with Fridericia correction (QTcF) to *Kcnd2* expression levels. CTRL, control rats; PAH-P, pulmonary arterial hypertension (PAH) rats treated with placebo; PAH-A, PAH rats treated with apelin analog. Data are mean ± SD (A-C) and interquartile range (D).

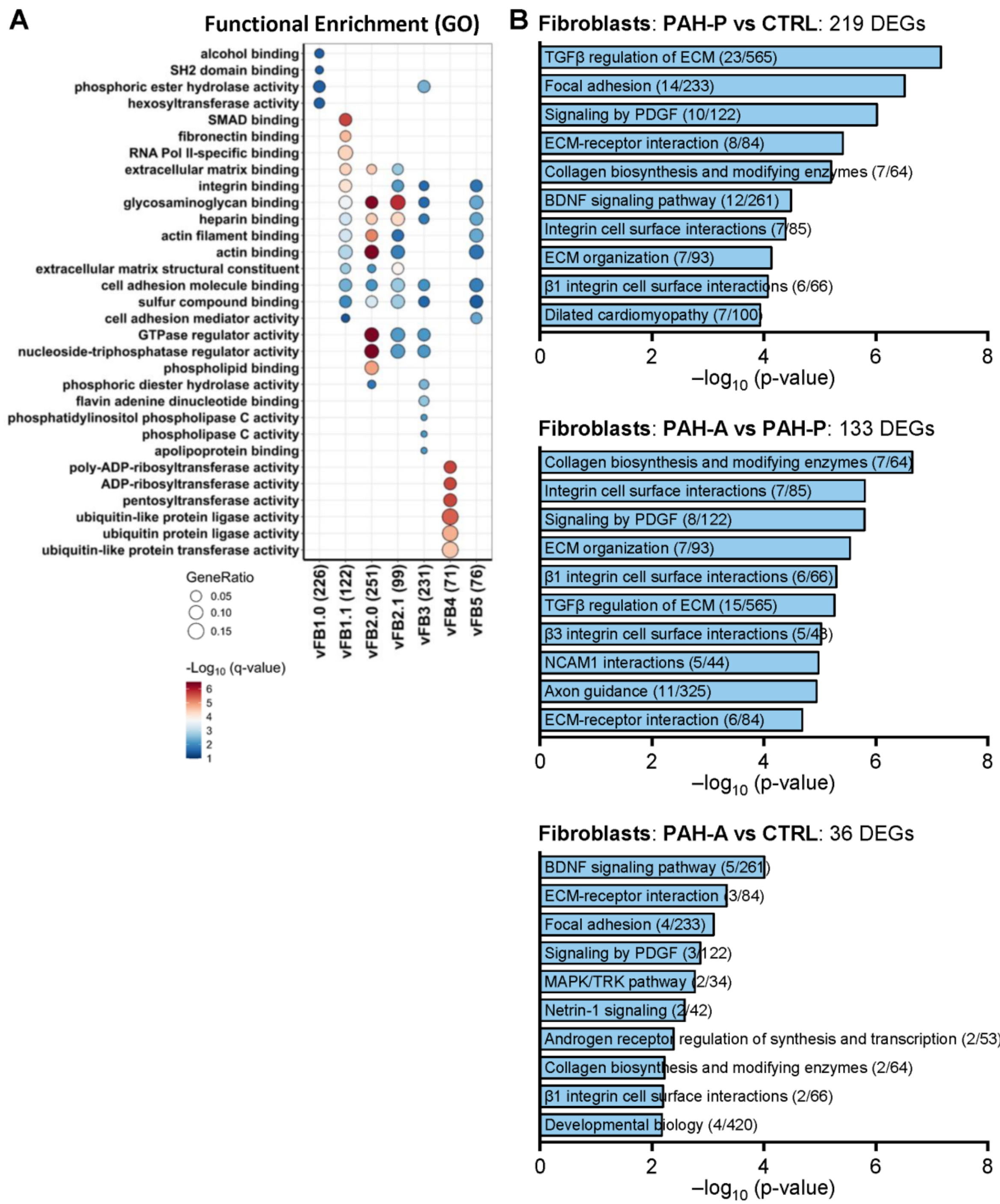

Supplemental Figure 19. **Functional enrichment of marker genes and pathway enrichment analysis of differentially expressed genes in RV fibroblasts (pseudo-bulk)**. (**A**) Functional enrichment analysis of fibroblast marker genes to elucidate functional roles of RV fibroblast cell states using Cluster profiler pathway analysis. (**B**) BioPlanet pathway analysis of differentially expressed genes (DEGs) between PAH-P and CTRL, PAH-A and PAH-P, and PAH-A and CTRL rat RV fibroblasts. CTRL, control rats; PAH-P, pulmonary arterial hypertension (PAH) rats treated with placebo; PAH-A, PAH rats treated with apelin analog.

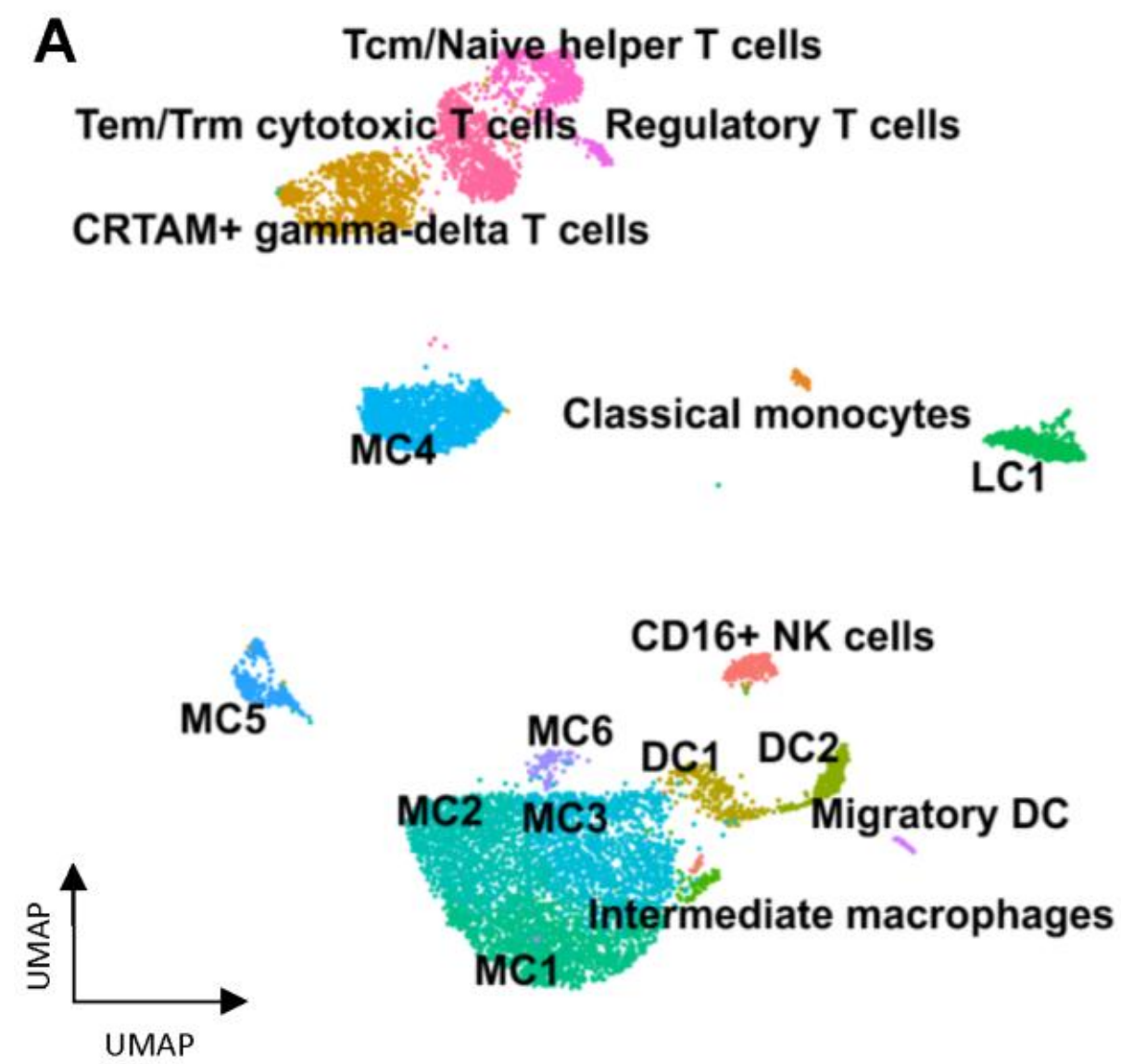

Supplemental Figure 20. **Immune and Mural cell states in the RV**. (**A**) UMAP of immune cell states. (**B**) UMAP of mural cell states (PC1.0 – PC4, SMC1, SMC2). CTRL, control rats; PAH-P, pulmonary arterial hypertension (PAH) rats treated with placebo; PAH-A, PAH rats treated with apelin analog.

Supplemental Videos

Video S1. Parasternal short axis video of the untreated pulmonary arterial hypertension heart. PAH-P, pulmonary arterial hypertension rats treated with placebo.

Video S2. Parasternal short axis video of pulmonary arterial hypertension heart treated with apelin analog. PAH-A, pulmonary arterial hypertension rats treated with apelin analog.
