# Supplementary material for: Apelin analog treatment reverses severe pulmonary arterial hypertension and right ventricular heart failure
Source: JCI Insight. 2026 Jun 18;11(15):e200684. doi: 10.1172/jci.insight.200684 (PMC13464015; doi:10.1172/jci.insight.200684)
Supplement: Supplementary file 2 [file jciinsight-11-200684-s349.pdf]

Full unedited gel for Figure 6D

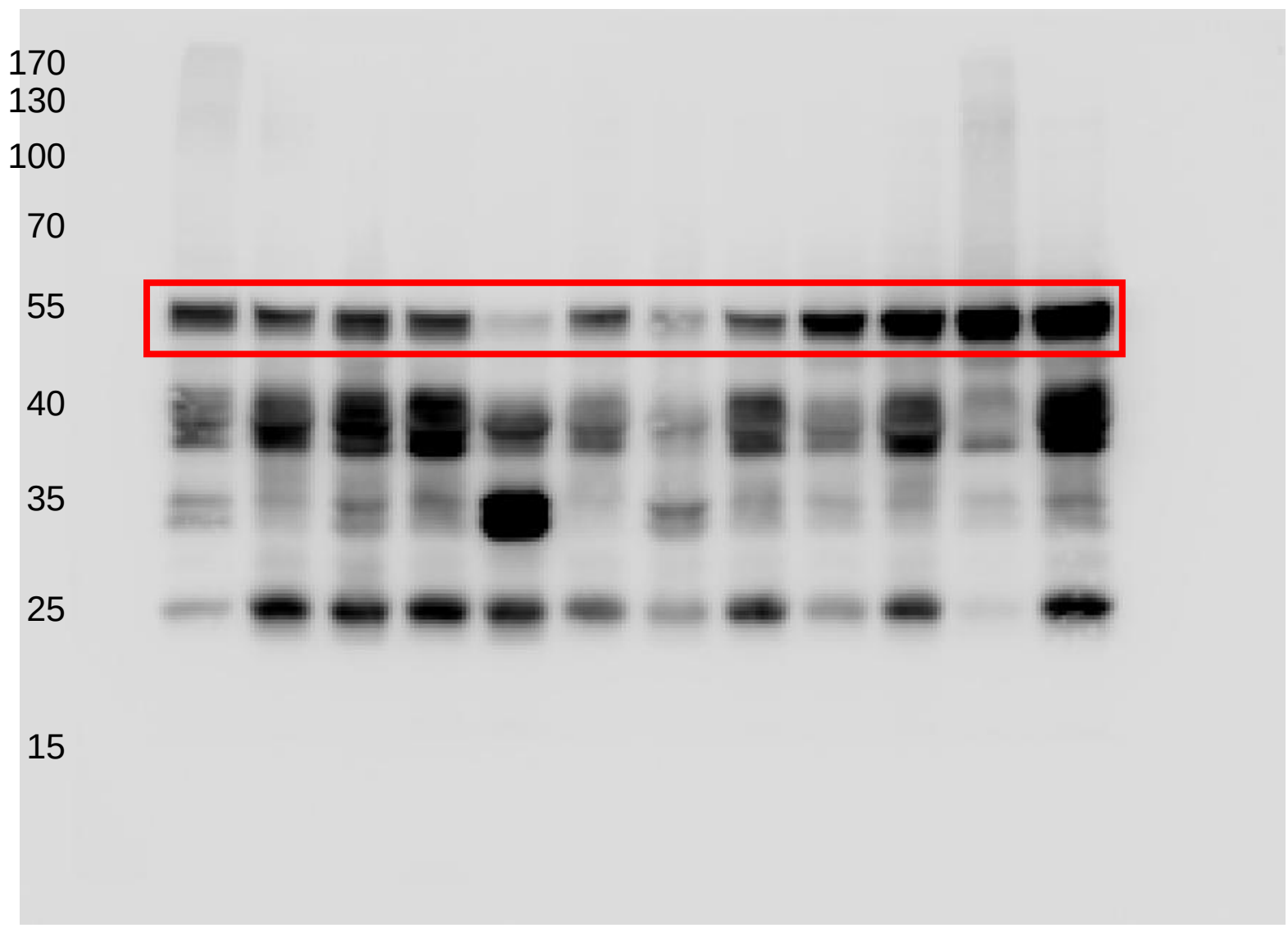

pSmad1-5 Lung

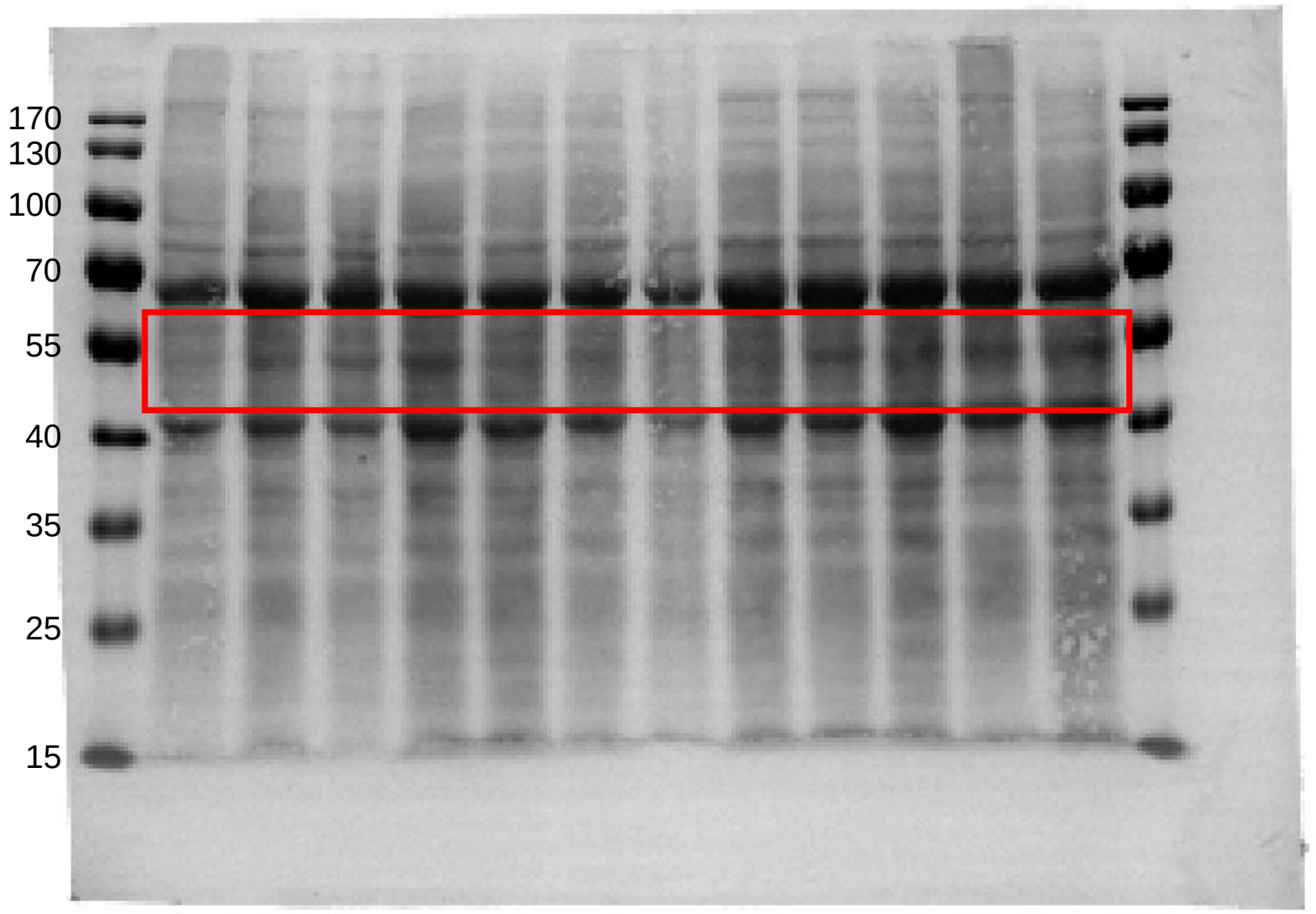

pSmad1-5 Lung (mem code)

Full unedited gel for Figure 11D

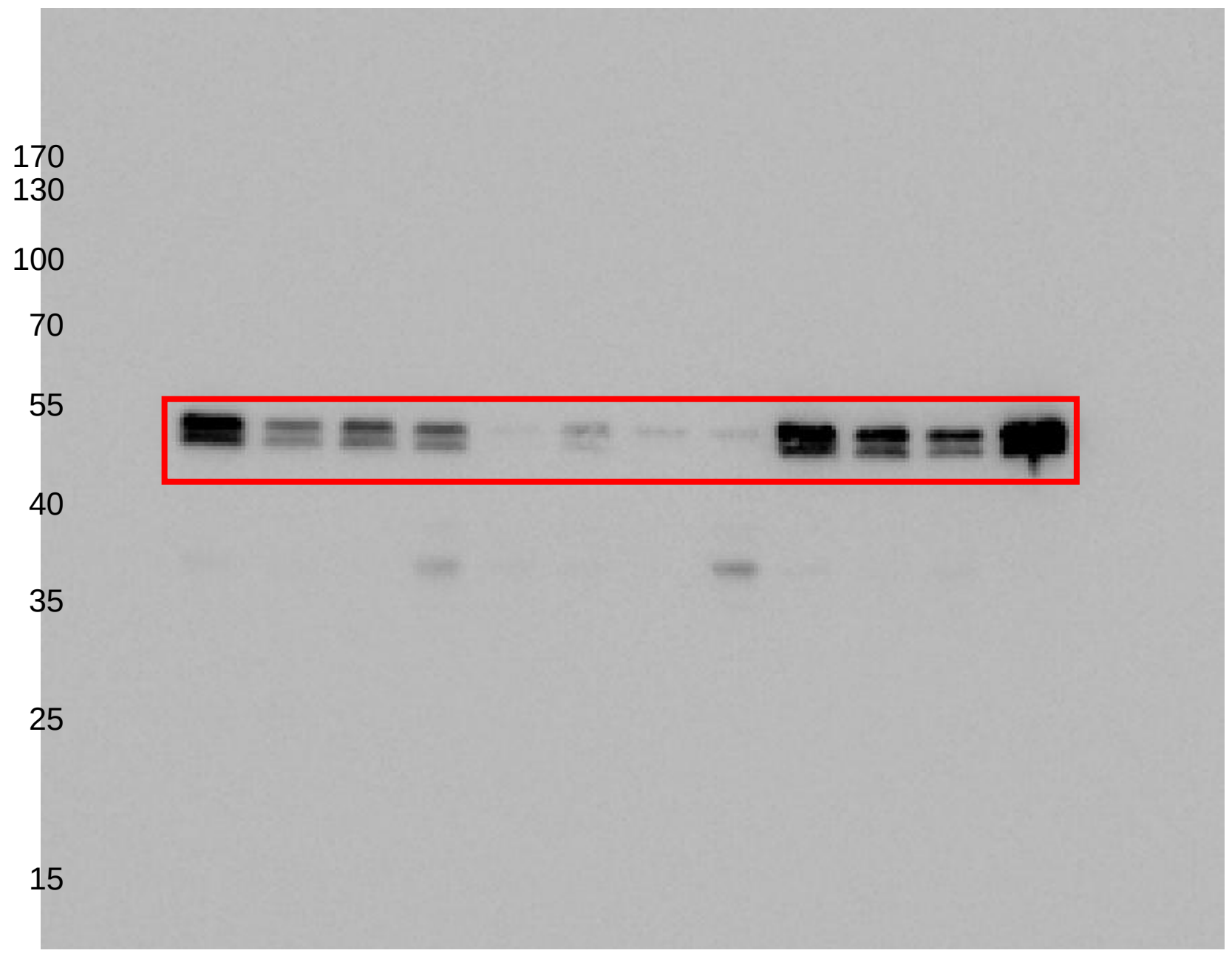

pSmad1-5 RV

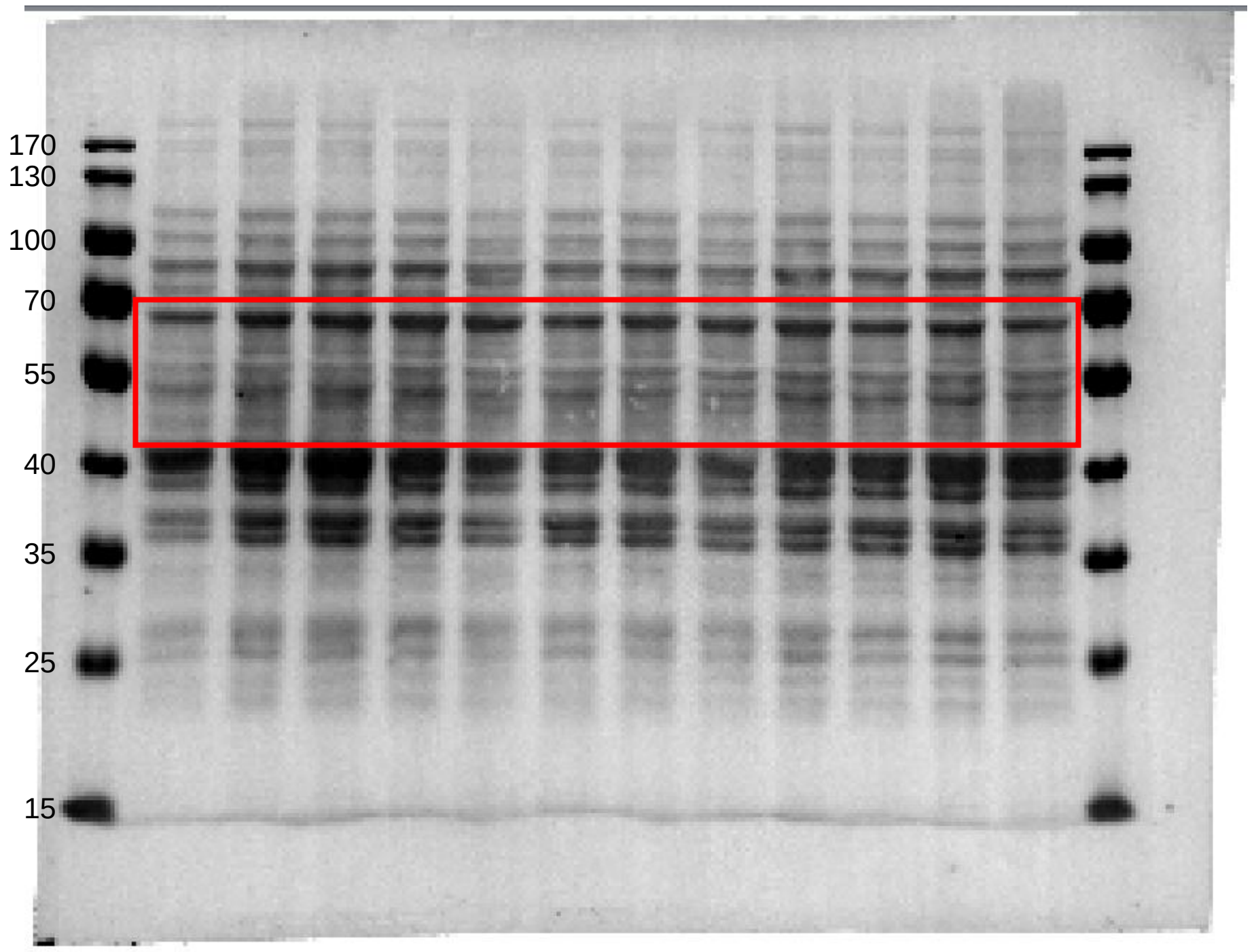

pSmad1-5 RV mem code

Full unedited gel for Figure 13E

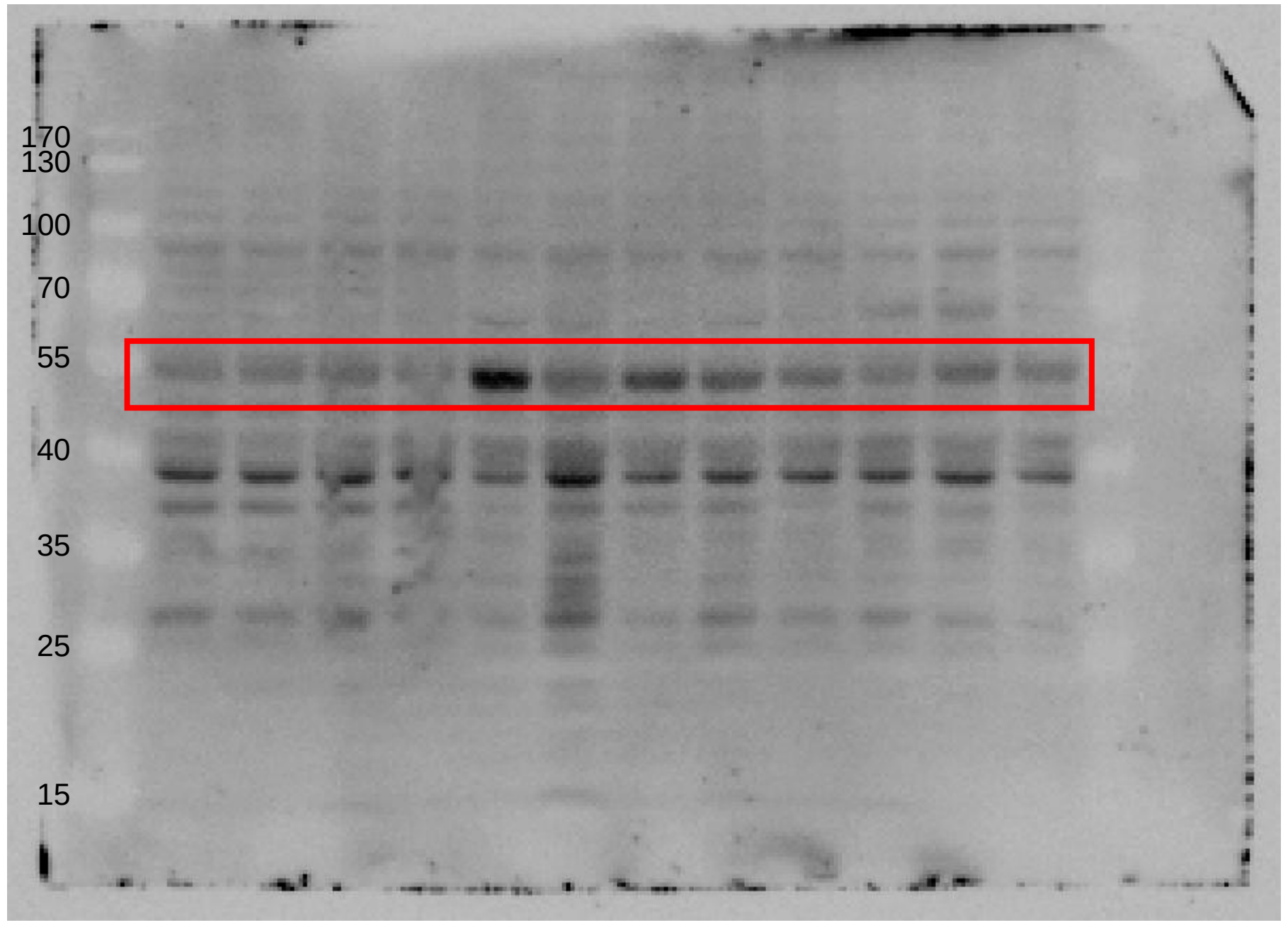

PDK4 RV

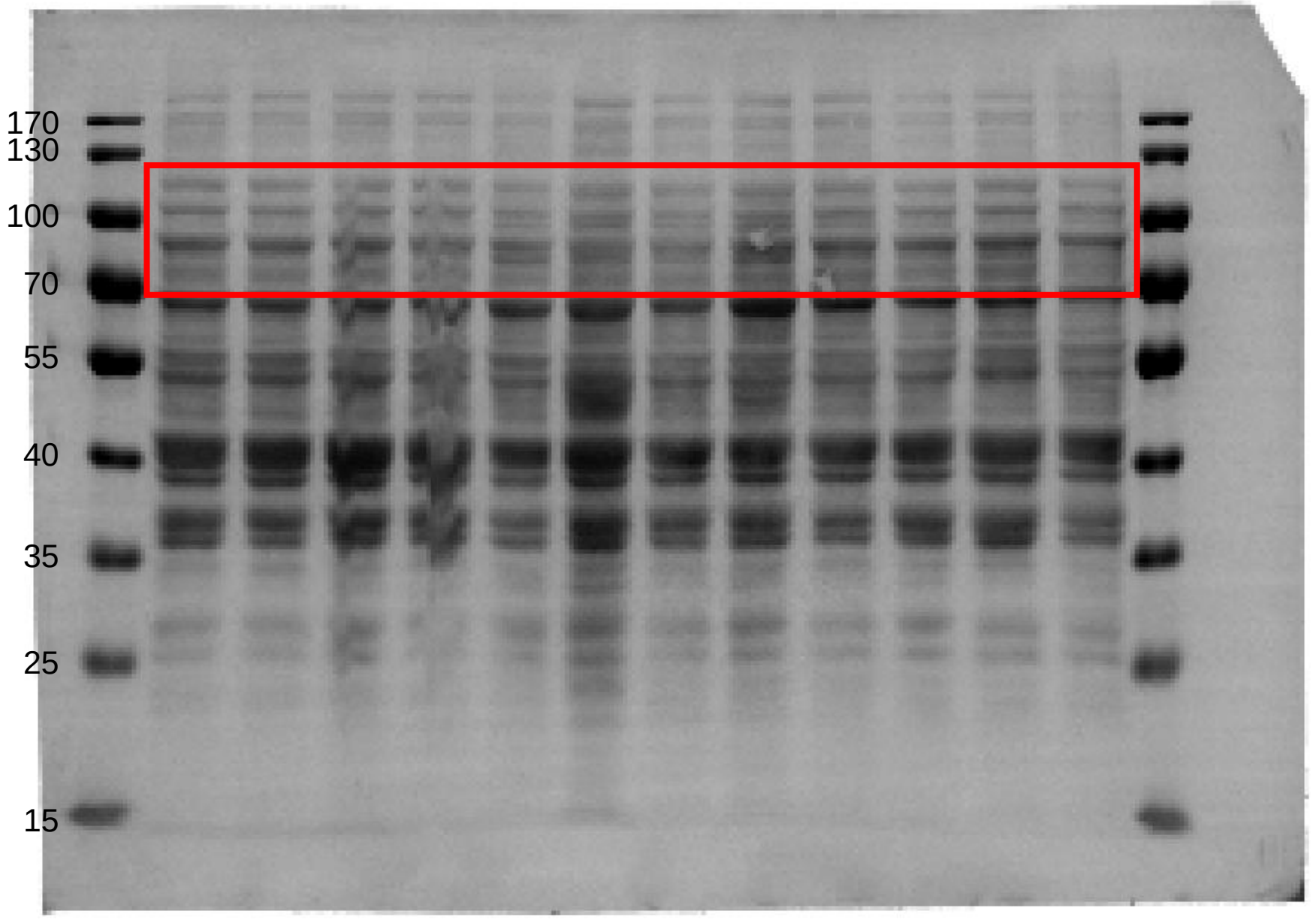

PDK4 RV (mem code)

Full unedited gel for Figure S10A

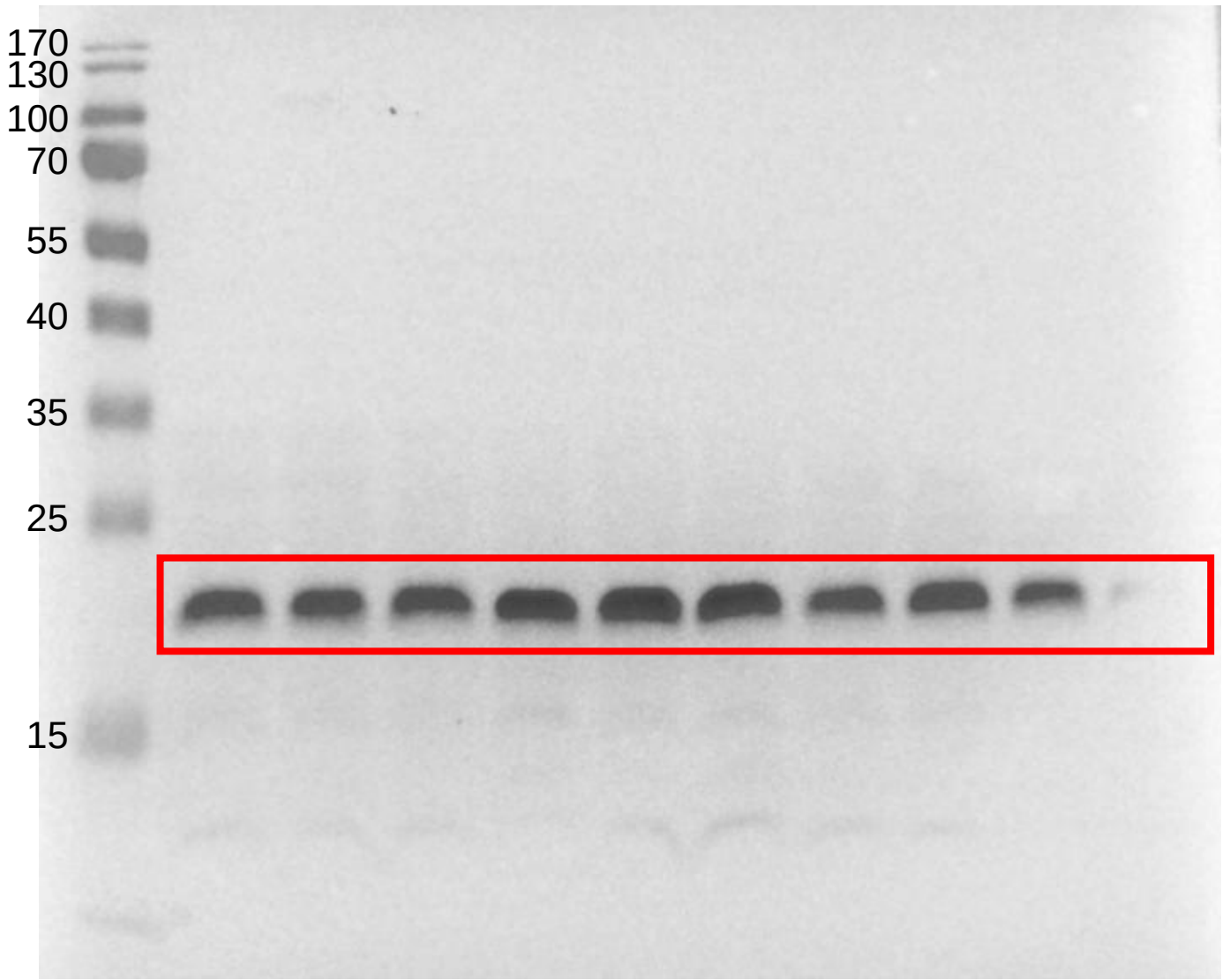

Apelin Lung

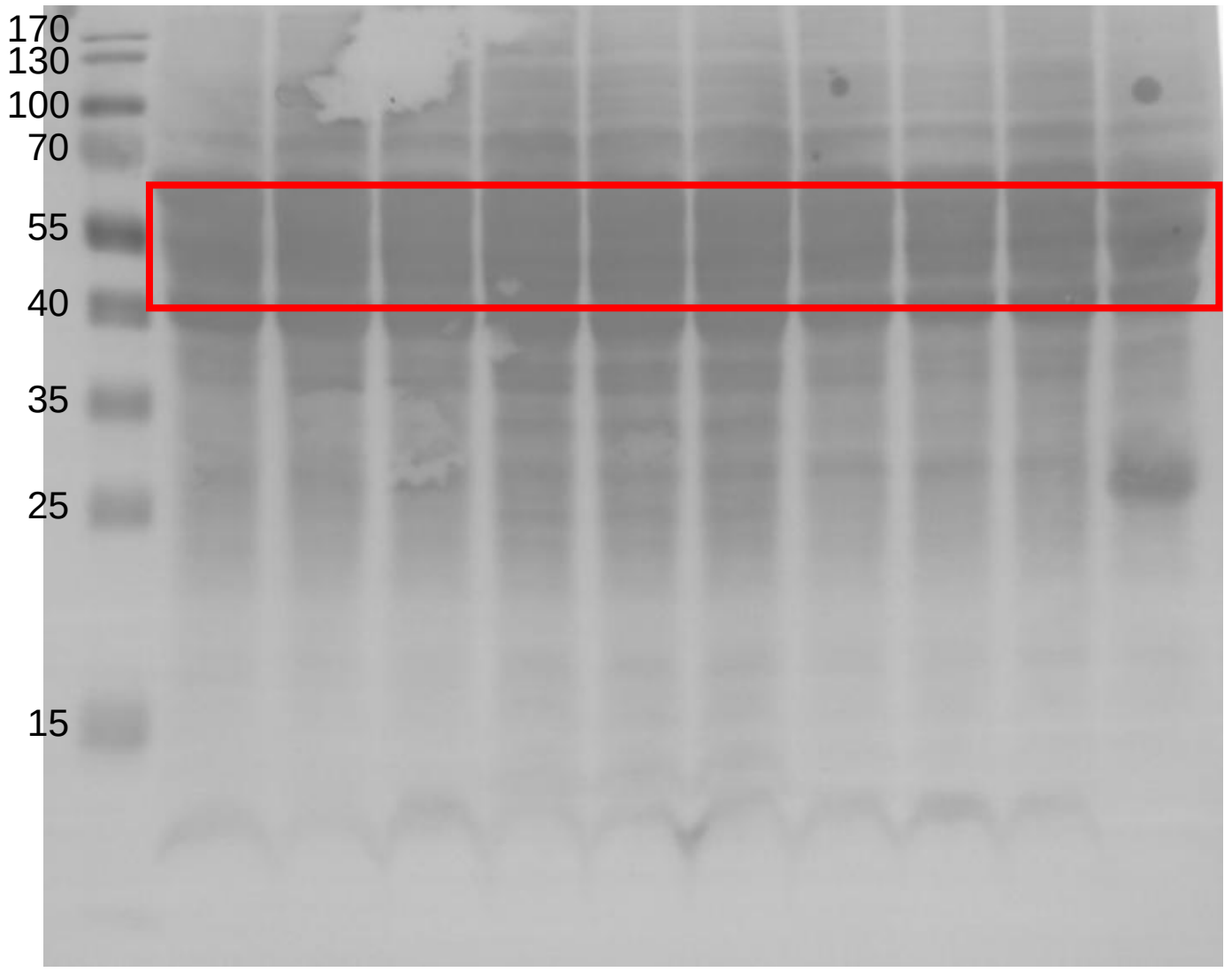

Apelin Lung (mem code)

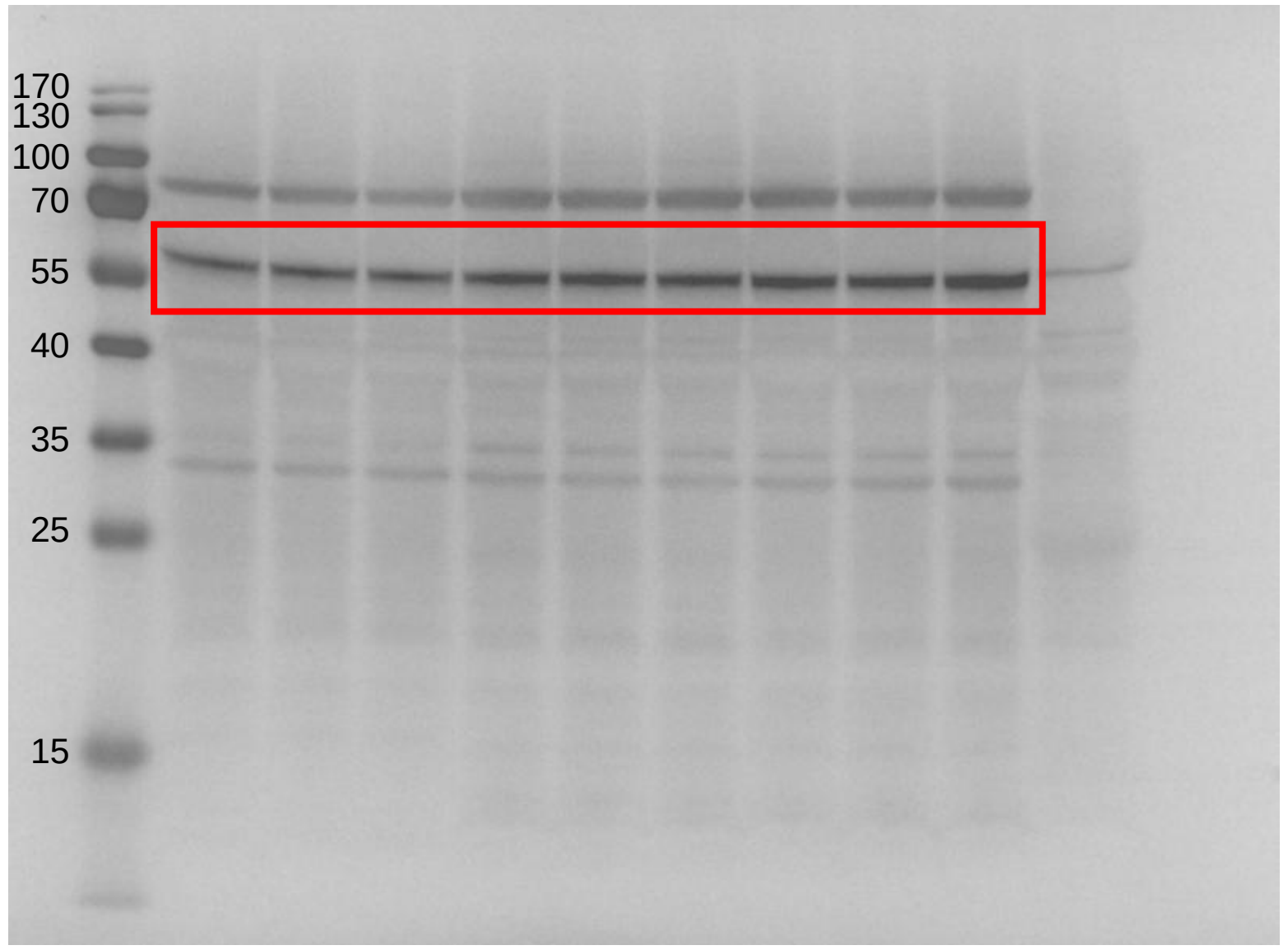

Apelin Receptor Lung

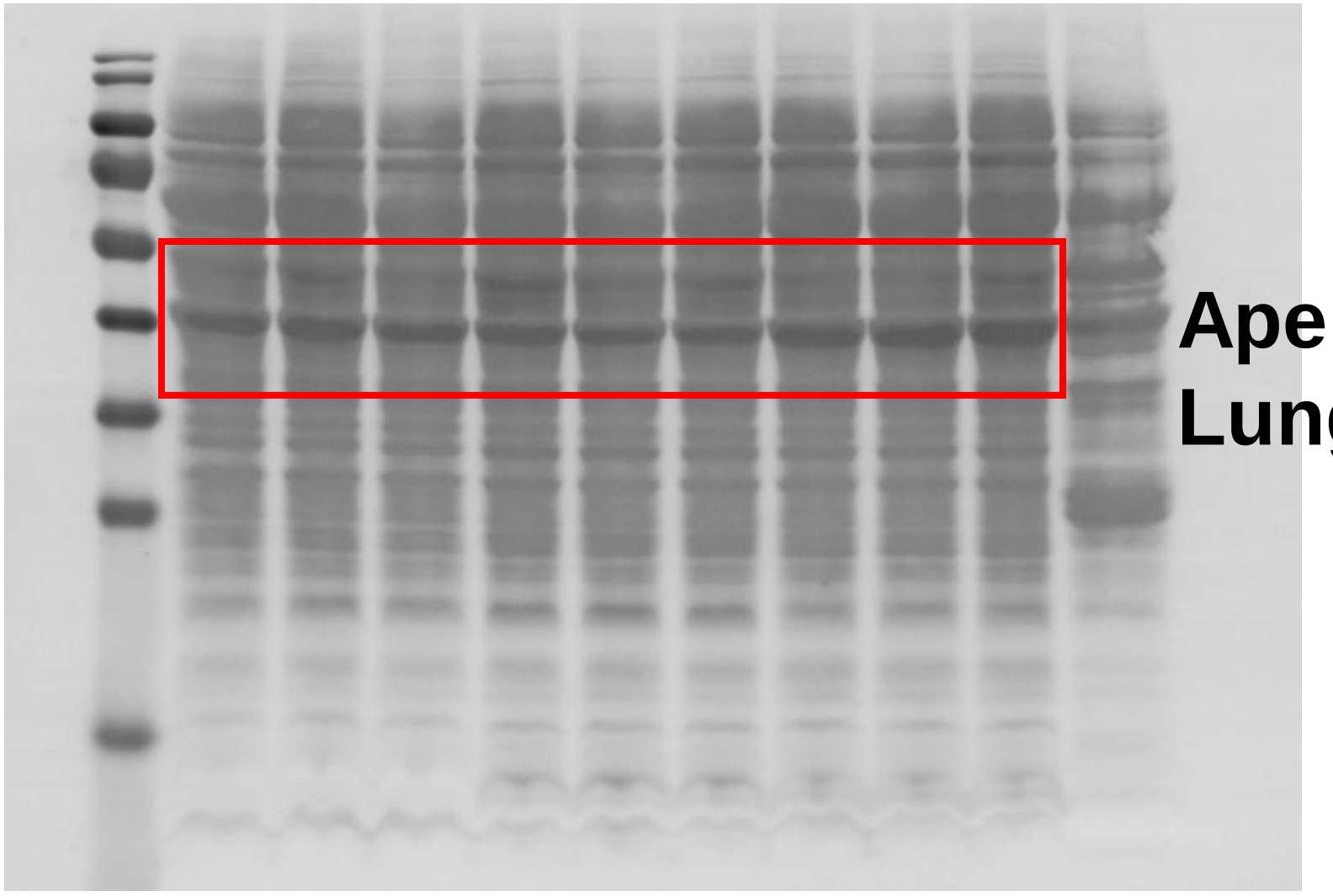

Apelin Receptor Lung (mem code)

Full unedited gel for Figure S12A

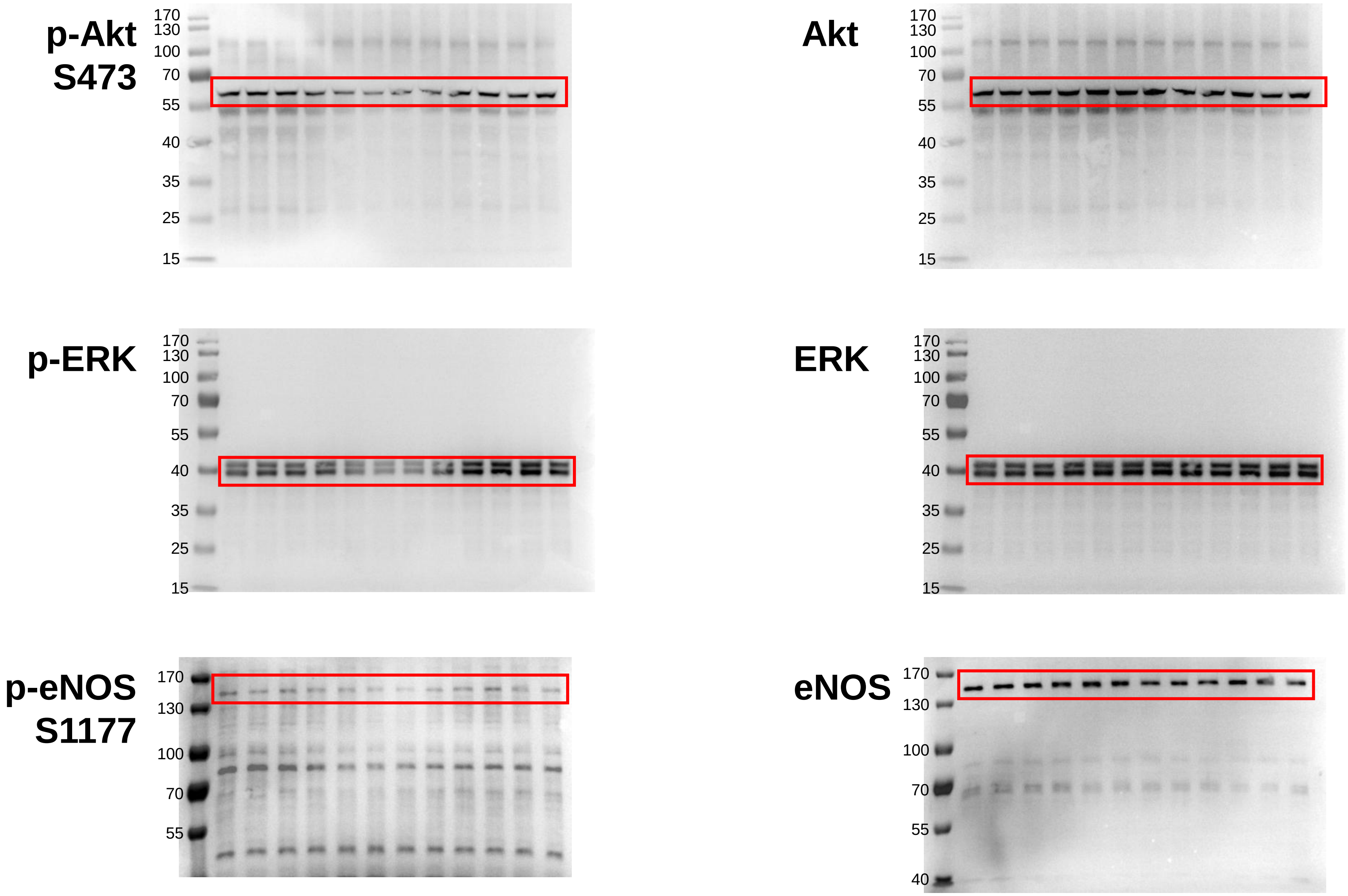

Full unedited gel for Figure S15B

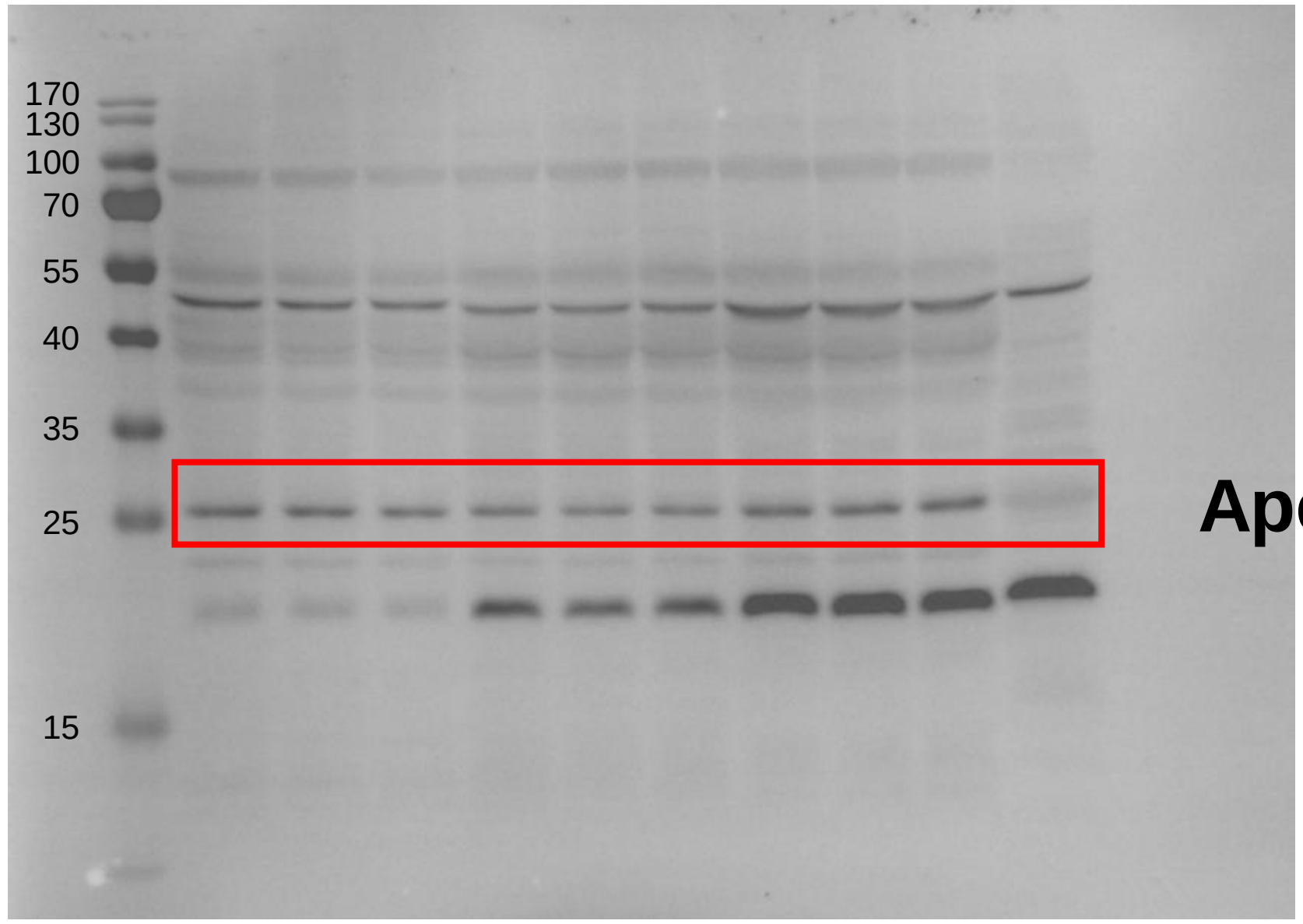

Apelin RV

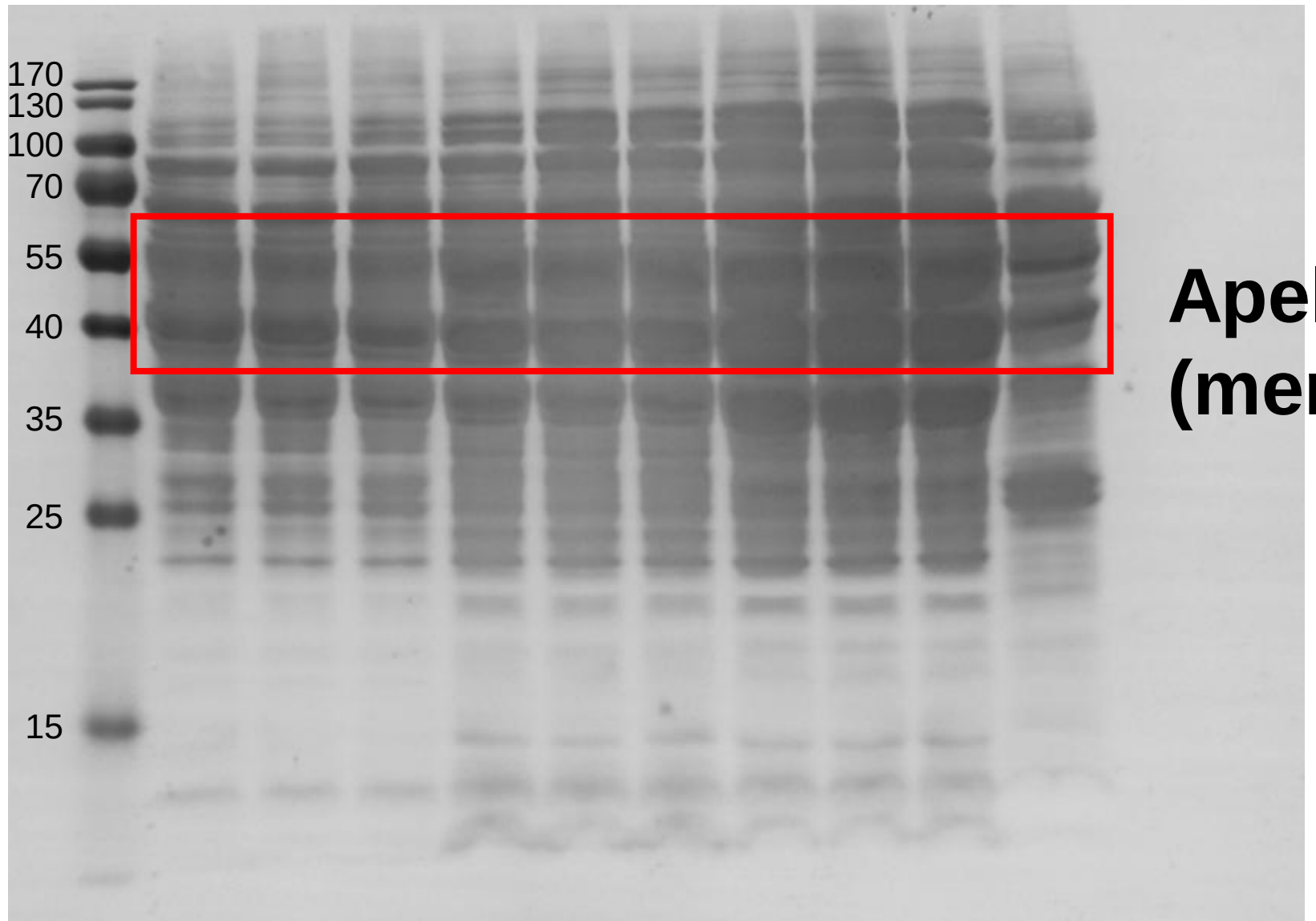

Apelin RV (mem code)

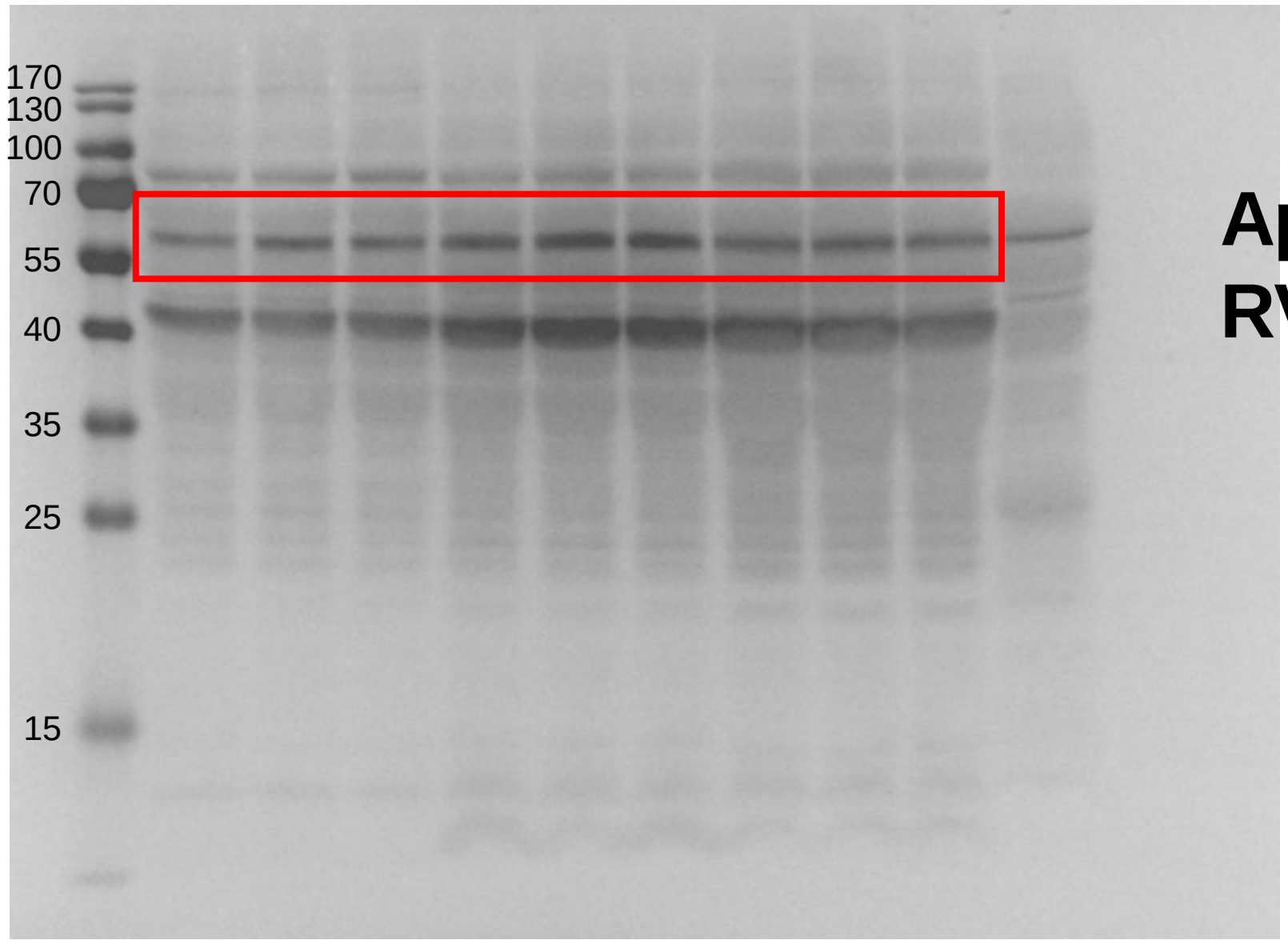

Apelin receptor RV

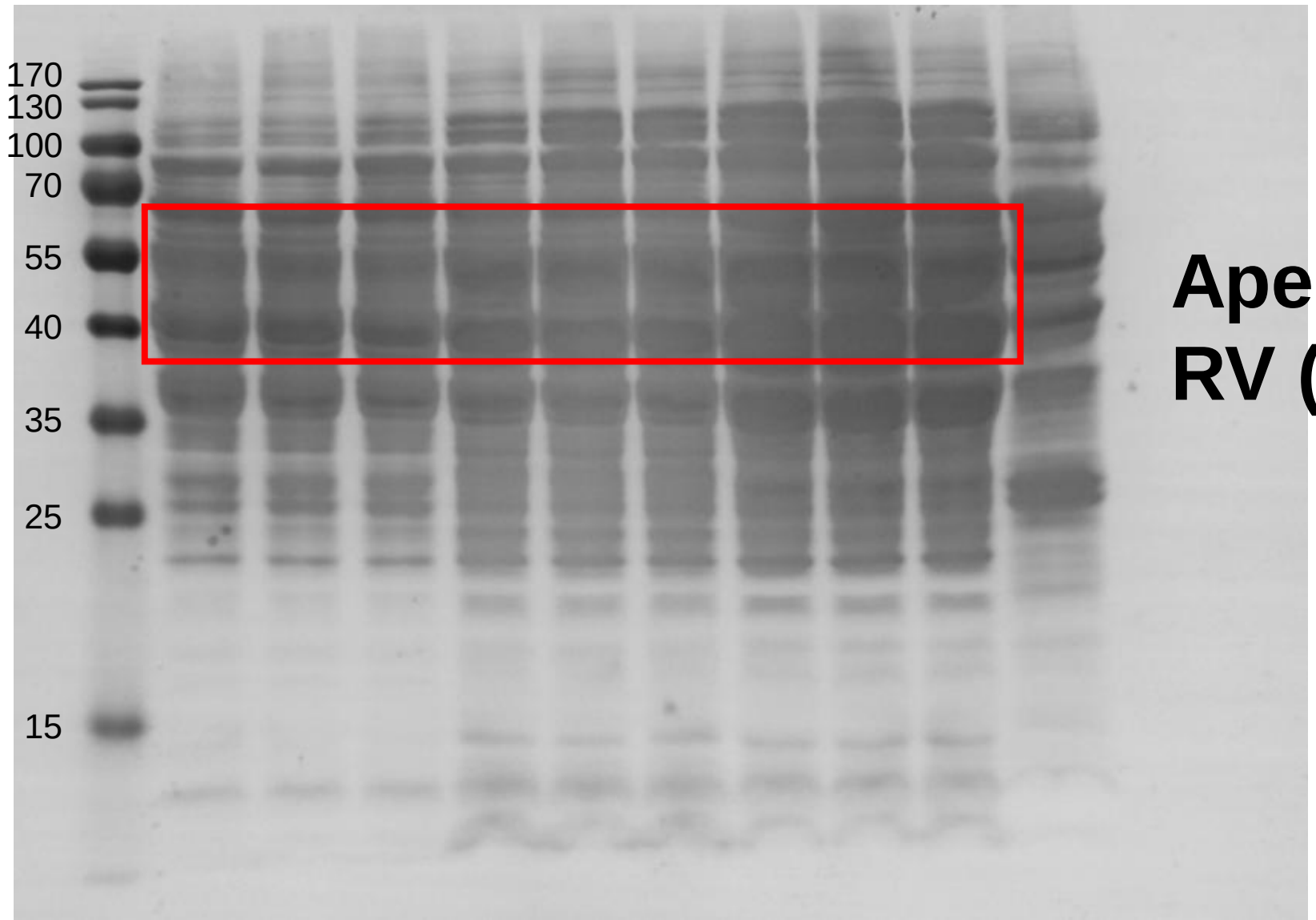

Apelin receptor RV (mem code)
